# IGF-1 and cardiometabolic diseases: a Mendelian randomisation study

**DOI:** 10.1007/s00125-020-05190-9

**Published:** 2020-06-16

**Authors:** Susanna C. Larsson, Karl Michaëlsson, Stephen Burgess

**Affiliations:** 1 Department of Surgical Sciences, Uppsala University, Epihubben, 75185 Uppsala, Sweden; 2 Unit of Cardiovascular and Nutritional Epidemiology, Institute of Environmental Medicine, Karolinska Institutet, Stockholm, Sweden; 3 Department of Public Health and Primary Care, University of Cambridge, Cambridge, UK; 4 MRC Biostatistics Unit, University of Cambridge, Cambridge, UK

**Keywords:** IGF-1, Insulin-like growth factor, Mendelian randomisation, Type 2 diabetes

## Abstract

**Aims/hypothesis:**

Abnormal serum IGF-1 levels are associated with an increased risk of type 2 diabetes and cardiovascular disease. However, the causal role of IGF-1 levels within the normal range in cardiometabolic disease remains unclear. We employed Mendelian randomisation to explore the associations between genetically predicted serum IGF-1 levels and cardiometabolic diseases.

**Methods:**

Serum IGF-1 levels were predicted using 416 SNPs associated with IGF-1 levels among 358,072 individuals in UK Biobank. Genetic association estimates for the outcomes were obtained from consortia of type 2 diabetes (74,124 cases, 824,006 controls), coronary artery disease (60,801 cases, 123,504 controls), heart failure (47,309 cases, 930,014 controls), atrial fibrillation (65,446 cases, 522,744 controls), and ischaemic stroke (60,341 cases, 454,450 controls).

**Results:**

Genetic predisposition to elevated serum IGF-1 levels was associated with higher risk of type 2 diabetes and coronary artery disease. The OR (95% CI) per SD increment in IGF-1 level was 1.14 (1.05, 1.24) for type 2 diabetes and 1.09 (1.02, 1.16) for coronary artery disease. The association between IGF-1 and coronary artery disease was attenuated after adjustment for type 2 diabetes (OR 1.06 [95% CI 1.00, 1.13]), suggesting that the association may be partly mediated via type 2 diabetes. There was limited evidence of associations between IGF-1 levels and heart failure, atrial fibrillation and ischaemic stroke.

**Conclusions/interpretation:**

This study found evidence that increased IGF-1 levels may be causally associated with higher risk of type 2 diabetes.

**Figure:**
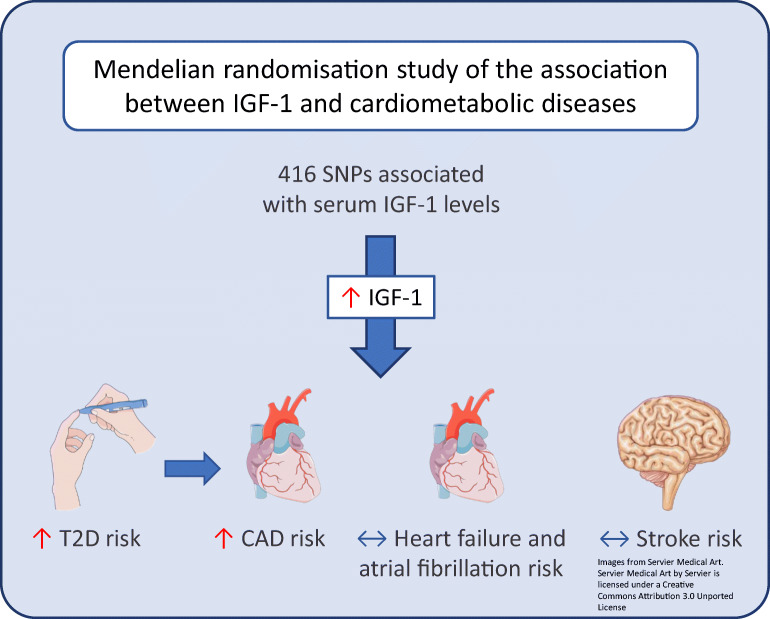
Graphical abstract

**Electronic supplementary material:**

The online version of this article (10.1007/s00125-020-05190-9) contains peer-reviewed but unedited supplementary material, which is available to authorised users.

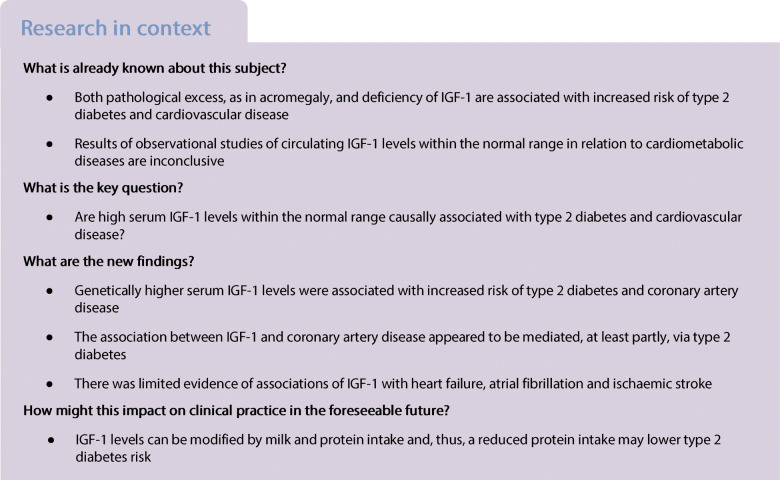



## Introduction

IGF-1 is a polypeptide hormone that is structurally similar to proinsulin. IGF-1 is synthesised primarily in the liver upon stimulation by growth hormone and is a key mediator of growth hormone-stimulated growth and other anabolic activities in many cells and tissues [1]. Both pathological excess, as in acromegaly, and deficiency of IGF-1 are associated with glucose intolerance, insulin resistance and increased risk of type 2 diabetes and cardiovascular morbidity and mortality [1, 2]. However, the effects of high serum IGF-1 levels within the normal range on cardiometabolic diseases remains unclear. Several observational studies [3–15], although not all [16–19], have reported the association of either low or high circulating total IGF-1 levels with type 2 diabetes and different cardiovascular diseases. Nevertheless, because underlying disease may influence IGF-1 levels, and observational studies are vulnerable to confounding, causality cannot be inferred based on available data.

Mendelian randomisation (MR) is a method to address causality in observational studies using one or multiple genetic variants affecting the risk factor as a genetic instrument for the effect of the risk factor on disease. Here, we used the MR design to investigate the associations of long-term increased IGF-1 levels with type 2 diabetes and major cardiovascular diseases. In secondary analyses, we explored the associations of genetically predicted IGF-1 levels with components of the metabolic syndrome. Given the important role of IGF-1 in growth, we also assessed the association of genetically predicted IGF-1 levels with height, as a positive control.

## Methods

### Genetic instrument for IGF-1

Instrumental variables for serum IGF-1 levels were selected from a genome-wide association study (GWAS) of 358,072 European-descent individuals in UK Biobank [20]. Among the genome-wide significant (*p* < 5 × 10^−8^) SNPs identified in that GWAS, we selected 416 SNPs after exclusion of correlated SNPs based on a linkage disequilibrium threshold of *R*^2^ <0.01. The SNPs were estimated to explain 9.4% of the variance in IGF-1 levels, and the *F* statistic was 80.9. In UK Biobank the mean (range) age of participants is 56.5 (37–73) years, the mean (SD) IGF-1 concentration is 21.4 (5.7) nmol/l and the IGF-1 concentration in the first and ninth decile is 14.2 nmol/l and 28.4 nmol/l, respectively.

### Outcome data sources

Summary-level data for the genetic associations with the outcomes were obtained from meta-analyses of GWASs [21–33] or UK Biobank for BP. We used the largest publicly available GWAS dataset for each exposure, except for the glycaemic traits where the largest GWAS dataset [34] only included 29 of the 416 SNPs for IGF-1. Information on the data sources is provided in Table 1. In brief, for the cardiometabolic diseases, we used data from the Atrial Fibrillation Consortium (AFGen) [26, 27], the Coronary Artery Disease Genome-wide Replication and Meta-analysis plus The Coronary Artery Disease Genetics consortium [24], the Diabetes Genetics Replication and Meta-analysis (DIAGRAM) consortium [21, 22], the Heart Failure Molecular Epidemiology for Therapeutic Targets (HERMES) consortium [28] and the MEGASTROKE consortium [25]. For atrial fibrillation and type 2 diabetes, we additionally used data from the FinnGen consortium [23], which had no sample overlap with the AFGen and DIAGRAM consortia. However, some studies in the FinnGen consortium were part of the other consortia and therefore not used for other outcomes. For SNPs that were unavailable in the outcome dataset, proxy SNPs in linkage disequilibrium (*R*^2^ ≥ 0.8) with the IGF-1-associated SNPs were used when available. Most GWASs adjusted for sex and genetic principal components. Summary statistics for the SNPs related to IGF-1 levels and the corresponding statistics for the cardiometabolic diseases are presented in electronic supplementary material (ESM) Tables 1–5. All studies included in the GWASs had been approved by an ethical review committee, and participants provided informed consent. This MR study was approved by the Swedish Ethical Review Authority.

**Table 1:** Data sources for the outcomes

Outcome	No. of cases	No. of controls	Population	No. of SNPs used for IGF-1	Consortium
Cardiometabolic disease					
Type 2 diabetes	74,124	824,006	European	416	DIAGRAM [21]
Type 2 diabetes^a^	26,676	132,532	European	416	DIAGRAM [22]
Type 2 diabetes^a^	11,006	82,655	European	393	FinnGen [23]
Coronary artery disease	60,801	123,504	Mixed	408	CARDIoGRAMplusC4D [24]
Atrial fibrillation	65,446	522,744	Mixed	416	AFGen (2018 dataset) [26]
Atrial fibrillation^a^	17,931	115,142	Mixed	416	AFGen (2017 dataset) [27]
Atrial fibrillation^a^	7244	56,378	European	393	FinnGen [23]
Heart failure	47,309	930,014	European	413	HERMES [28]
Ischaemic stroke	60,341	454,450	Mixed	398	MEGASTROKE [25]
Glycaemic traits^b^					
Fasting glucose	NA	46,186	European	297	MAGIC [29]
Fasting insulin	NA	38,238	European	298	MAGIC [29]
HOMA-IR	NA	46,187	European	297	MAGIC [29]
Serum lipids^b^					
HDL-cholesterol	NA	187,167	Mixed	296	GLGC [30]
LDL-cholesterol	NA	173,083	Mixed	296	GLGC [30]
Total cholesterol	NA	187,365	Mixed	296	GLGC [30]
Triacylglycerols	NA	177,861	Mixed	296	GLGC [30]
BP^b^					
Systolic BP	NA	317,754	European	414	UK Biobank (Neale Lab)
Diastolic BP	NA	317,756	European	414	UK Biobank (Neale Lab)
Body composition^b^					
BMI	NA	339,224	Mixed	297	GIANT [31]
Waist circumference	NA	224,459	European	297	GIANT [32]
WHR	NA	224,459	European	297	GIANT [32]
Height	NA	253,288	European	296	GIANT [33]

^a^This data source did not have participant overlap with the data source for the exposure (IGF-1 levels) and was used as a supplementary analysis

^b^Summary association estimates for these outcomes were obtained through the MR-Base platform (database version 0.2.0, 17 December 2017) [40]

CARDIoGRAMplusC4D, Coronary Artery Disease Genome-wide Replication and Meta-analysis plus The Coronary Artery Disease Genetics; GIANT, Genetic Investigation of Anthropometric Traits; GLGC, Global Lipids Genetics Consortium; HERMES, Heart Failure Molecular Epidemiology for Therapeutic Targets; MAGIC, Meta-Analyses of Glucose and Insulin-related traits Consortium

### Statistical analysis

The MR estimates were obtained using the inverse-variance weighted (IVW) method under a multiplicative random-effects model. The *I*^2^ statistic was used to assess heterogeneity between the estimates obtained from individual SNPs. Sensitivity analyses using the weighted median [35], MR Pleiotropy Residual Sum and Outlier (PRESSO) [36] and MR-Egger [35] methods were conducted. The weighted median method provides a valid estimate if at least 50% of the weight originates from non-pleiotropic SNPs. The MR-PRESSO method and MR-Egger method can adjust for potential outliers and directional pleiotropy, respectively. To address possible pleiotropy with other members of the IGF axis, we conducted a sensitivity analysis excluding three SNPs in the *IGFBP3* or *IGF2* gene regions. Multivariable MR analysis was used to evaluate the direct effect of IGF-1 levels on cardiometabolic diseases not mediated by fasting insulin levels, insulin resistance or height. This analysis was carried out to assess whether any association between IGF-1 and the cardiometabolic diseases could be mediated by those factors. Multivariable MR analysis [37] was also used to estimate the direct effect of IGF-1 levels on coronary artery disease not mediated via type 2 diabetes.

Due to partial sample overlap in the data source for IGF-1 and the largest GWASs for type 2 diabetes [21] and atrial fibrillation [26], we performed a supplementary analysis using data from previous smaller GWASs for these outcomes [22, 27] and the FinnGen consortium [23] that did not include UK Biobank. The heart failure dataset also included UK Biobank participants but no other large and publicly available GWAS dataset without UK Biobank was available, and the FINRISK study from the FinnGen consortium were included in the heart failure GWAS. There was no sample overlap for the data source for IGF-1 and the datasets for coronary artery disease and ischaemic stroke.

All presented results are expressed per SD increase in IGF-1 levels (equivalent to about 5.7 nmol/l in UK Biobank). The statistical analyses were performed using the mrrobust package in Stata [38], the MendelianRandomization package in R [39] and the MR-Base platform [40].

## Results

### Cardiometabolic diseases

**Fig. 1:**
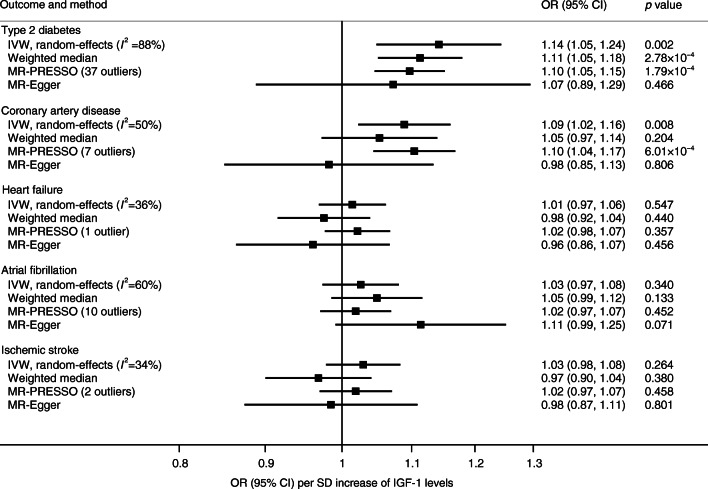
Associations between genetically predicted IGF-1 levels and type 2 diabetes and cardiovascular diseases

Genetically predicted IGF-1 levels were positively associated with type 2 diabetes and coronary artery disease in the primary (IVW) analysis (Fig. 1). The OR (95% CI) per SD increase in genetically predicted IGF-1 levels was 1.14 (1.05, 1.24) for type 2 diabetes and 1.09 (1.02, 1.16) for coronary artery disease. The association with type 2 diabetes was confirmed when using data from a smaller dataset from the DIAGRAM consortium that did not include UK Biobank (OR 1.13 [95% CI 1.03, 1.25]) and in the FinnGen consortium (OR 1.20 [95% CI 1.08, 1.34]). Furthermore, the association between genetically predicted IGF-1 levels and type 2 diabetes was consistent in sensitivity analyses based on the weighted median and MR-PRESSO methods (Fig. 1). The OR estimate for type 2 diabetes did not change after exclusion of three SNPs in the *IGFBP3* or *IGF2* gene regions. The MR-Egger analysis showed no indication of directional pleiotropy (*p* for intercept = 0.464). The association between IGF-1 and coronary artery disease persisted in the MR-PRESSO analysis but not in the weighted median and MR-Egger analyses; however, the precision was low in the MR-Egger analysis and there was no evidence of directional pleiotropy (*p* for intercept = 0.116) (Fig. 1). Exclusion of the three SNPs in the *IGFBP3* or *IGF2* gene regions did not essentially alter the results for coronary artery disease (OR 1.10 [95% CI 1.03, 1.17]).

There was no strong evidence for associations between IGF-1 levels and heart failure (OR 1.01 [95% CI 0.97, 1.06]), atrial fibrillation (OR 1.03 [95% CI 0.97, 1.08]) or ischaemic stroke (OR 1.03 [95% CI 0.98, 1.08]) (Fig. 1). For atrial fibrillation, the OR was 1.05 (95% CI 0.98, 1.13) when using a smaller GWAS dataset from the AFGen that did not include UK Biobank, and the OR was 0.92 (95% CI 0.82, 1.04) in the FinnGen consortium. For subtypes of ischaemic stroke, the ORs (95% CIs) were 0.93 (0.83, 1.04) for large artery stroke, 1.10 (0.99, 1.06) for small vessel stroke and 1.06 (0.96, 1.16) for cardioembolic stroke.

### Components of the metabolic syndrome and height

**Fig. 2:**
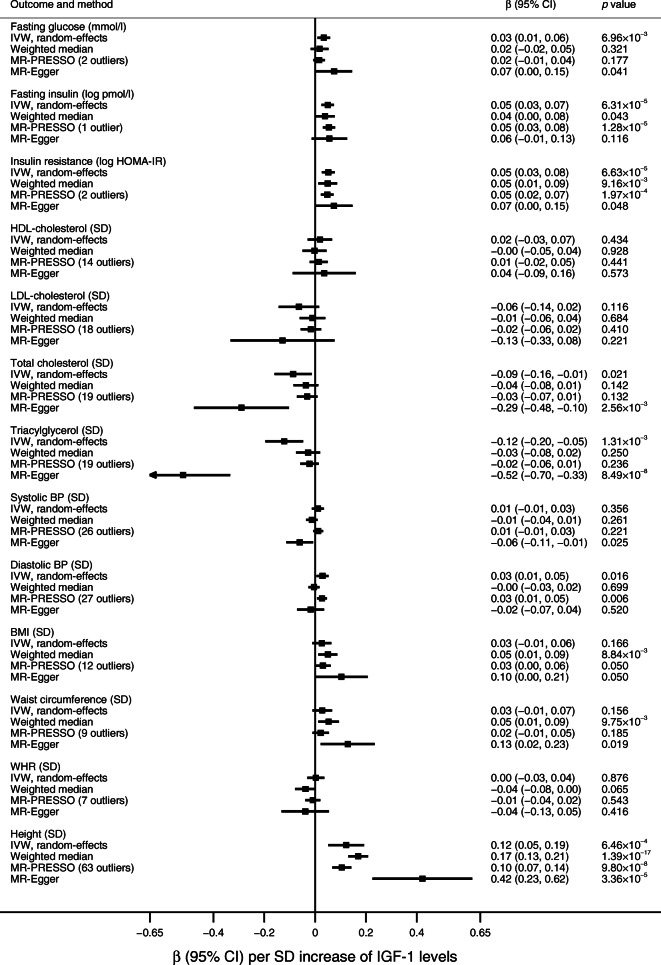
Associations between genetically predicted IGF-1 levels and components of the metabolic syndrome and height. Log indicates natural logarithmic transformed levels (log_*e*_)

There was strong or suggestive evidence that genetically higher IGF-1 levels were associated with higher fasting glucose (*p* = 6.96 × 10^−3^) and insulin (*p* = 6.31 × 10^−5^) levels, increased insulin resistance (*p* = 6.63 × 10^−5^), higher diastolic BP (*p* = 0.016), lower total cholesterol (*p* = 0.021) and triacylglycerol (*p* = 1.31 × 10^−3^) levels and higher height (*p* = 6.46 × 10^−4^) in the primary analysis (Fig. 2). The most consistent associations across sensitivity analyses were with fasting insulin levels, insulin resistance and height (Fig. 2).

### Direct effects of IGF-1

The associations of genetically predicted IGF-1 levels with type 2 diabetes and coronary artery disease were similar after adjustment for insulin levels (ESM Table 6) or insulin resistance (ESM Table 7), whereas adjustment for height resulted in somewhat stronger associations (ESM Table 8). The lack of associations with the other outcomes persisted after adjustment for insulin levels, insulin resistance and height (ESM Tables 6–8). The association between genetically predicted IGF-1 levels and coronary artery disease was attenuated after adjustment for type 2 diabetes (OR 1.06 [95% CI 1.00, 1.13], *p* = 0.063).

## Discussion

This MR study showed that genetically higher IGF-1 levels were associated with increased risk of type 2 diabetes and coronary artery disease, though results for coronary artery disease were not consistent across all sensitivity analyses. Genetically higher IGF-1 levels were additionally associated with some components of the metabolic syndrome, the most robust association being with fasting insulin and insulin resistance.

Our findings are in line with the results of two nested case–control studies, which demonstrated that high IGF-1 levels were associated with a statistically significant [10] or a suggestive [15] increased risk of type 2 diabetes. Another nested case–control study found that high levels of free IGF-1 were associated with higher risk of type 2 diabetes in individuals with insulin levels above the median, but with lower risk in individuals with insulin levels below the median [41]. However, a null association between IGF-1 levels and type 2 diabetes has also been reported [9, 18] and a cohort study of 615 participants showed that the 51 participants who developed impaired glucose tolerance or type 2 diabetes during a follow-up period of 4.5 years had lower IGF-1 levels compared with those who did not develop impaired glucose tolerance [5]. The reason for these conflicting results is unclear. Nevertheless, the relatively small sample sizes (ranging from around 50 to 800 cases) in previous studies and potential reverse causality, whereby the disease process caused changes in IGF-1 levels several years before the clinical diagnosis of type 2 diabetes, may have resulted in null or spurious findings.

Our findings for IGF-1 levels and cardiovascular disease corroborate those of some but not all observational studies. In a cohort study of 2901 Swedish men (including 589 incident cardiovascular events), both high (>80th percentile) and low (<20th percentile) IGF-1 levels were associated with increased risk of any cardiovascular event, and high but not low IGF-1 levels were associated with a statistically significant higher risk of coronary artery disease [12]. Similarly, in a nested case–control study of US women (245 myocardial infarction cases and 490 matched controls), the multivariable-adjusted RR (95% CI) of myocardial infarction was 2.09 (1.17, 3.72) and 1.46 (0.79, 2.72) for the third and fourth highest quartile of IGF-1 levels, respectively, when compared with the bottom quartile [8]. IGF-1 levels were also positively associated with coronary artery disease in a cross-sectional study of 6773 German adults [9]. In contrast, some other cross-sectional and nested case–control studies (including 57–374 cases) reported that elevated IGF-1 levels were associated with lower prevalence or incidence of coronary artery disease [3, 4, 11], heart failure [6], atrial fibrillation [13] and ischaemic stroke [7, 14]. Other cross-sectional and nested case–control studies showed a null association between IGF-1 levels and coronary artery disease (167–1013 cases) [16, 17, 19] and cerebrovascular events (273 cases) [12]. The inconsistent results might reflect small sample sizes in most previous studies, reverse causality or residual confounding.

The main strength of this study is the MR design, which reduced confounding and reverse causation bias. Another strength is the large sample sizes for both the data source (UK Biobank) used to derive the genetic association with IGF-1 levels and for the data sources used for genetic associations with the outcomes. This, along with the strong genetic instrument for serum IGF-1 levels, resulted in high precision of the results in the primary analyses. The estimates obtained from the MR-Egger analysis were imprecise and should be interpreted with caution. A further strength is that a large number of SNPs was available as instrumental variables for IGF-1 levels. We could therefore conduct several sensitivity analyses to evaluate pleiotropy. A limitation of this study is that the genetic instrument was for total IGF-1 levels rather than the free and bioavailable IGF-1 fraction, which may be more strongly associated with type 2 diabetes and cardiovascular diseases. Another shortcoming is that we could not investigate whether there is a U- or J-shaped relationship between IGF-1 levels and cardiometabolic diseases and insulin resistance, as suggested by a few observational studies [12, 42]. However, confounding by height might have resulted in non-linear associations in those studies.

In this study, we found that the association between genetically predicted serum IGF-1 levels and type 2 diabetes was partially attenuated after adjustment for fasting insulin levels or insulin resistance through multivariable MR analysis. This suggests that elevated IGF-1 levels may increase the risk of type 2 diabetes in part through insulin resistance. Further research is needed to understand other possible mechanisms underlying the association between IGF-1 and type 2 diabetes. The association between IGF-1 levels and coronary artery disease appeared to be mediated, at least partly, via type 2 diabetes.

Evidence indicates that IGF-1 levels may be modified by milk and protein intake [43–47]. A meta-analysis of eight randomised controlled trials showed a statistically significant 13.8 ng/ml (equivalent to about 1.8 nmol/l) difference in IGF-1 levels when comparing the milk intervention group with the control group [44]. Furthermore, several randomised controlled trials have demonstrated that increased dietary protein intake or whey protein (one of the two proteins in milk) supplementation increase circulating IGF-1 levels [45–47]. Intake of protein, particularly from animal sources, has been found to be positively associated with type 2 diabetes risk in observational studies [48]. Additionally, evidence from experimental, observational and MR studies indicates that high circulating levels of branched-chain amino acids, found in high levels in for example whey protein, increase the risk of type 2 diabetes [49].

### Conclusions

This MR study found evidence of a causal association between increased IGF-1 levels within the normal range and higher risk of type 2 diabetes. This finding may have public health and clinical implications as IGF-1 levels may be modified by milk and protein intake [43–47].

## Electronic supplementary material

ESM (PDF 884 kb)

## Data Availability

All data analysed in this study are based on publicly available summary statistics data provided by genetic consortia.

## Abbreviations

AFGen: Atrial Fibrillation Consortium
DIAGRAM: Diabetes Genetics Replication and Meta-analysis
GWAS: Genome-wide association study
IVW: Inverse-variance weighted
MR: Mendelian randomisation
PRESSO: Pleiotropy Residual Sum and Outlier
