# Supplementary material for: IGF-1 and cardiometabolic diseases: a Mendelian randomisation study
Source: Diabetologia. 2020 Jun 16;63(9):1775–82. doi: 10.1007/s00125-020-05190-9 (PMC7406523; doi:10.1007/s00125-020-05190-9)
Supplement: Supplementary file 1 — (PDF 884 kb) [file 125_2020_5190_MOESM1_ESM.pdf]

ESM Table 1. Summary statistics of the single-nucleotide polymorphisms associated with insulin-like growth factor-1 and their associations with type 2 diabetes

					IGF-1			Type 2 diabetes		
**SNP**	**Chr:position**	**Nearby gene**	**EA**	**OA**	**Beta**	**SE**	***p***	**Beta**	**SE**	***p***
rs112436634	1:10637709	PEX14	T	C	0,016	0,003	1.749e-09	-0,008	0,007	0,220
rs599839	1:109822166	CELSR2	G	A	0,031	0,003	9.629e-26	0,021	0,008	0,006
rs140604451	1:110216436	GSTM2	G	A	0,054	0,008	3.524e-11	-0,041	0,019	0,034
rs17037452	1:11895675	CLCN6	A	G	0,023	0,003	1.658e-11	0,020	0,009	0,023
rs1127313	1:154556425	ADAR	G	A	0,024	0,003	1.394e-21	0,003	0,006	0,660
rs7528548	1:154934665	PYGO2	C	T	0,054	0,009	7.954e-10	0,014	0,021	0,510
rs75907879	1:16170903	SPEN	T	C	0,024	0,004	1.425e-10	-0,002	0,010	0,820
rs77369503	1:163027266	RGS4	G	A	0,045	0,007	1.397e-10	0,002	0,019	0,910
rs36086195	1:16510894	ARHGEF19-AS1	C	T	0,019	0,003	1.359e-13	0,005	0,007	0,450
rs75681856	1:174916323	RABGAP1L	T	C	0,023	0,004	5.171e-09	0,003	0,010	0,740
rs12749024	1:176522365	PAPPA2	T	C	0,075	0,004	7.72e-100	0,010	0,009	0,280
rs11577063	1:179341999	AXDND1	T	G	0,020	0,003	1.511e-11	0,001	0,008	0,860
rs143885630	1:183482785	SMG7	G	A	0,030	0,004	1.358e-15	0,004	0,010	0,670
rs7545345	1:205690941	NUCKS1	C	T	0,026	0,004	1.032e-12	0,017	0,010	0,078
rs2724373	1:207999200	C1orf132	C	T	0,019	0,003	1.436e-12	0,011	0,007	0,110
rs10159299	1:208258102	PLXNA2	C	T	0,018	0,003	5.112e-12	0,000	0,006	0,980
rs10779509	1:209728370	RP1-272L16.1	C	T	0,014	0,003	2.109e-08	-0,002	0,006	0,720
rs12723255	1:21233570	EIF4G3	C	T	0,017	0,003	3.229e-11	-0,004	0,007	0,560
rs1223763	1:214334246	RP11-53A1.3	T	G	0,024	0,003	8.461e-13	0,010	0,008	0,220
rs6701954	1:22022176	USP48	T	G	0,014	0,003	3.497e-08	0,006	0,007	0,350
rs903908	1:2202967	SKI	C	T	0,016	0,003	2.176e-10	-0,011	0,007	0,091
rs12141189	1:221053545	HLX	T	C	0,045	0,003	4.135e-53	0,016	0,007	0,033
rs4306136	1:221608720	RP11-103C3.1	A	G	0,017	0,003	1.997e-11	-0,016	0,007	0,017
rs708108	1:228189855	WNT3A	T	C	0,015	0,003	5.872e-09	0,004	0,007	0,520
rs684818	1:234854779	RP4-781K5.7	T	C	0,024	0,003	9.658e-21	0,007	0,006	0,280
rs2802951	1:235014058	RN7SL668P	A	G	0,016	0,003	4.970e-09	-0,009	0,007	0,170
rs2075995	1:23847464	E2F2	C	A	0,014	0,003	9.790e-09	0,013	0,006	0,044
rs7517340	1:243710190	AKT3	C	T	0,035	0,003	2.123e-26	0,008	0,008	0,340
rs2802330	1:26466831	PDIK1L	G	A	0,031	0,003	4.632e-21	-0,037	0,009	0,000
rs6659176	1:27239920	NR0B2	C	G	0,042	0,005	1.354e-19	-0,040	0,011	0,001
rs569356	1:29136686	OPRD1	G	A	0,027	0,004	5.953e-14	0,023	0,009	0,012
rs3131646	1:40383552	MYCL	G	T	0,016	0,003	1.710e-08	-0,007	0,007	0,310
rs61780439	1:41490177	SLFNL1	G	A	0,021	0,003	5.323e-12	-0,001	0,008	0,910
rs2819336	1:44015809	PTPRF	T	C	0,027	0,003	1.550e-25	-0,004	0,007	0,510
rs7539178	1:65383002	JAK1	C	A	0,026	0,004	1.365e-12	0,007	0,009	0,450
rs1046011	1:65898996	LEPROT	T	C	0,021	0,003	3.303e-14	-0,005	0,007	0,520
rs1430753	1:68692642	WLS	A	G	0,021	0,003	7.085e-11	-0,007	0,008	0,370
rs165316	1:91533297	RPL5P6	G	A	0,073	0,003	9.66e-119	0,009	0,008	0,250
rs11165778	1:91859801	HFM1	A	G	0,016	0,003	1.492e-08	-0,008	0,007	0,290
rs17393144	1:9210262	MIR34AHG	A	G	0,016	0,003	9.319e-09	0,010	0,007	0,170

rs1825813	1:92708973	C1orf146	A	G	0,023	0,003	1.879e-13	0,020	0,008	0,013
rs73954943	2:111890432	BCL2L11	A	G	0,031	0,005	1.063e-09	0,034	0,013	0,011
rs7578633	2:113978650	PAX8	T	C	0,018	0,003	3.379e-12	-0,003	0,007	0,670
rs17050272	2:121306440	AC073257.2	G	A	0,024	0,003	3.201e-20	-0,031	0,007	0,000
rs58387407	2:152924773	CACNB4	G	A	0,018	0,003	3.502e-08	0,016	0,008	0,044
rs35135518	2:16120506	AC010145.4	T	C	0,029	0,004	1.521e-12	0,003	0,010	0,790
rs2674492	2:172422338	CYBRD1	A	G	0,014	0,003	4.835e-08	0,013	0,006	0,037
rs17400325	2:178565913	PDE11A	C	T	0,054	0,006	9.393e-17	0,004	0,017	0,810
rs12710648	2:17989500	SMC6	A	G	0,017	0,003	5.703e-12	0,009	0,006	0,170
rs6435156	2:203425475	BMPR2	C	T	0,024	0,003	2.045e-17	-0,016	0,007	0,024
rs62182127	2:219279588	VIL1	A	G	0,019	0,003	2.712e-14	-0,002	0,006	0,760
rs11678946	2:222302730	EPHA4	A	C	0,014	0,003	3.143e-08	0,007	0,006	0,310
rs4402747	2:225457173	NEU2	A	G	0,016	0,003	2.960e-10	0,004	0,006	0,510
rs17323117	2:230162971	NEU2	G	A	0,029	0,005	7.980e-10	0,016	0,011	0,170
rs1465529	2:231039037	SP110	T	C	0,019	0,003	1.386e-12	0,004	0,007	0,570
rs6437249	2:242175331	HDLBP	C	T	0,019	0,003	4.559e-12	-0,003	0,007	0,700
rs6760135	2:26088769	ASXL2	T	C	0,050	0,003	1.194e-63	0,020	0,008	0,008
rs1260326	2:27730940	GCKR	C	T	0,063	0,003	9.66e-133	0,067	0,007	0,000
rs11677980	2:30522137	LBH	G	A	0,015	0,003	3.043e-08	0,012	0,007	0,092
rs7574340	2:40621239	SLC8A1	T	C	0,017	0,003	2.971e-10	0,012	0,007	0,074
rs6544549	2:42693056	KCNG3	T	C	0,024	0,004	1.592e-10	0,013	0,010	0,150
rs62136965	2:44347953	RNU6-566P	C	T	0,037	0,006	6.429e-10	0,014	0,015	0,360
rs3791679	2:56096892	EFEMP1	G	A	0,018	0,003	4.338e-09	0,006	0,008	0,400
rs12471768	2:64928603	SERTAD2	C	T	0,022	0,003	1.639e-15	0,014	0,007	0,052
rs702878	2:65702609	AC074391.1	A	G	0,014	0,003	1.815e-08	0,042	0,006	0,000
rs35641591	2:70323994	PCBP1-AS1	C	T	0,050	0,006	1.575e-14	0,003	0,016	0,840
rs6749680	2:73685852	ALMS1	A	G	0,015	0,003	9.868e-09	-0,009	0,007	0,190
rs62280667	3:101084604	SENP7	C	T	0,028	0,003	2.477e-26	0,005	0,007	0,480
rs62263345	3:107252190	BBX	A	G	0,028	0,004	1.640e-14	-0,012	0,010	0,220
rs7625680	3:11378069	ATG7	A	G	0,015	0,003	7.693e-09	0,015	0,007	0,025
rs13069961	3:124358715	KALRN	G	A	0,018	0,003	3.680e-09	0,010	0,008	0,220
rs1822825	3:12449963	PPARG	G	A	0,014	0,003	2.886e-08	0,003	0,006	0,590
rs687339	3:135932359	KRT18P35	T	C	0,040	0,003	2.666e-40	0,035	0,008	0,000
rs811332	3:138078348	MRAS	C	T	0,019	0,003	2.011e-09	-0,017	0,008	0,036
rs55717031	3:138848505	MRPS22	G	T	0,032	0,003	2.474e-31	-0,010	0,007	0,150
rs6440008	3:141154542	ZBTB38	T	C	0,035	0,003	1.113e-41	0,014	0,007	0,039
rs2607748	3:14158725	CHCHD4	C	T	0,017	0,003	3.289e-11	-0,001	0,006	0,880
rs73238159	3:142078759	XRN1	C	T	0,025	0,004	1.059e-11	0,017	0,010	0,079
rs13073970	3:170630520	EIF5A2	T	G	0,025	0,003	2.016e-15	-0,057	0,008	0,000
rs504603	3:172142232	BZW1P1	C	T	0,029	0,005	2.402e-09	0,002	0,012	0,890
rs56062334	3:172299226	LINC02068	T	C	0,017	0,003	1.839e-11	0,001	0,007	0,840
rs9819762	3:178914879	PIK3CA	T	C	0,019	0,003	1.212e-08	0,001	0,008	0,950
rs2268829	3:185989567	DGKG	G	A	0,018	0,003	3.038e-09	0,008	0,008	0,280
rs66707192	3:186382065	HRG	G	A	0,018	0,003	4.415e-10	-0,007	0,007	0,320
rs11717397	3:23368583	UBE2E2	G	A	0,015	0,003	2.572e-09	0,000	0,006	0,960

rs4678497	3:33213119	SUSD5	C	T	0,017	0,003	1.959e-10	-0,003	0,007	0,680
rs11928797	3:33457493	UBP1	A	C	0,030	0,004	2.428e-14	-0,002	0,010	0,840
rs12491473	3:46989904	CCDC12	G	A	0,020	0,003	5.913e-15	0,036	0,006	0,000
rs112893170	3:57211863	FEZF2	T	C	0,020	0,003	1.527e-10	-0,006	0,008	0,510
rs7628689	3:88216647	C3orf38	G	A	0,029	0,003	6.825e-17	0,030	0,009	0,001
rs3772102	3:98502628	ST3GAL6	G	T	0,020	0,003	5.954e-16	-0,019	0,006	0,003
rs6532798	4:100054827	ADH4	T	C	0,037	0,003	1.364e-41	-0,001	0,007	0,930
rs1229984	4:100239319	ADH1B	T	C	0,104	0,009	1.219e-34	-0,068	0,021	0,001
rs62342064	4:104665972	RP11-119H12.3	C	T	0,022	0,004	1.836e-09	0,005	0,010	0,610
rs17429745	4:106038169	RP11-556I14.1	G	T	0,026	0,003	2.045e-21	-0,003	0,007	0,700
rs3804173	4:121719923	PRDM5	G	A	0,020	0,003	4.484e-13	0,012	0,007	0,072
rs111443396	4:124773202	LINC01091	C	T	0,026	0,004	2.015e-10	0,013	0,010	0,200
rs7667562	4:129133826	LARP1B	C	A	0,016	0,003	3.770e-09	0,018	0,007	0,013
rs6827641	4:145653694	HHIP	T	C	0,014	0,003	2.623e-08	0,003	0,006	0,690
rs6853741	4:148982559	ARHGAP10	A	G	0,024	0,003	1.676e-16	-0,009	0,007	0,210
rs62334147	4:169345005	DDX60L	C	T	0,019	0,003	4.261e-09	0,003	0,008	0,720
rs4394044	4:186607420	SORBS2	T	C	0,014	0,003	4.708e-08	0,005	0,006	0,420
rs13108218	4:3443931	HGFAC	G	A	0,017	0,003	5.920e-11	-0,010	0,007	0,150
rs1055582	4:39700173	UBE2K	C	T	0,027	0,003	1.578e-27	-0,001	0,006	0,870
rs62302688	4:46448465	GABRA2	G	A	0,039	0,004	4.057e-20	-0,001	0,011	0,920
rs976002	4:69343287	TMPRSS11E	A	G	0,036	0,003	4.672e-35	0,006	0,008	0,420
rs1902023	4:69536084	UGT2B15	A	C	0,025	0,003	1.951e-23	-0,009	0,007	0,190
rs2280099	4:90035549	TIGD2	G	A	0,025	0,003	1.923e-14	0,008	0,008	0,340
rs35036084	4:97552791	RP11-145G20.1	T	C	0,017	0,003	8.218e-11	0,011	0,007	0,110
rs26822	5:102518795	PPIP5K2	G	A	0,017	0,003	9.845e-11	0,045	0,007	0,000
rs73271090	5:132313550	CTB-1I21.1	G	A	0,044	0,003	7.844e-39	0,018	0,009	0,043
rs329122	5:133864599	JADE2	A	G	0,018	0,003	1.312e-12	0,037	0,006	0,000
rs11242236	5:134586980	C5orf66	G	A	0,025	0,003	1.957e-22	-0,013	0,006	0,049
rs3734166	5:137665323	CDC25C	A	G	0,028	0,003	8.063e-22	0,020	0,007	0,007
rs258775	5:142564823	ARHGAP26	A	C	0,025	0,003	3.394e-14	0,023	0,008	0,007
rs2042253	5:143059433	MIR5197	T	C	0,023	0,003	1.017e-14	0,004	0,007	0,600
rs35668185	5:168256455	SLIT3	T	C	0,056	0,003	3.402e-73	-0,005	0,008	0,530
rs13168379	5:173382761	CPEB4	A	G	0,031	0,005	6.148e-10	0,008	0,013	0,530
rs17714046	5:180661980	TRIM41	C	T	0,042	0,006	1.197e-12	-0,004	0,015	0,790
rs9292578	5:35230075	PRLR	C	A	0,040	0,006	3.195e-10	-0,006	0,016	0,710
rs6895953	5:39084471	RP11-357F12.1	G	A	0,024	0,003	5.167e-21	0,005	0,006	0,400
rs72758321	5:41464841	PLCXD3	G	A	0,047	0,006	3.926e-15	-0,007	0,016	0,680
rs6180	5:42719239	GHR	A	C	0,035	0,003	6.019e-43	0,006	0,006	0,380
rs12520263	5:44122508	RP11-357F12.1	T	G	0,017	0,003	2.579e-09	-0,013	0,007	0,068
rs7719168	5:53292390	15ARL	C	A	0,030	0,004	2.429e-14	0,038	0,010	0,000
rs28650790	5:55861464	C5orf67	T	C	0,018	0,003	4.108e-08	0,069	0,008	0,000
rs1498603	5:58333125	PDE4D	T	G	0,031	0,005	1.170e-09	-0,006	0,013	0,670
rs11954036	5:59028853	PDE4D	T	C	0,037	0,003	1.936e-44	0,014	0,007	0,035
rs80170948	5:64020316	SREK1IP1	G	T	0,039	0,006	2.221e-09	-0,060	0,017	0,000
rs2227819	5:76012745	F2R	T	C	0,022	0,004	4.322e-08	-0,010	0,010	0,320

rs12108803	5:77158507	TBCA	G	T	0,033	0,006	1.015e-08	-0,002	0,015	0,870
rs840809	5:87173927	CTD-2232E5.2	A	C	0,016	0,003	1.183e-08	0,018	0,007	0,015
rs13178887	5:88355993	MEF2C-AS1	T	C	0,023	0,003	2.712e-19	0,014	0,007	0,029
rs2366398	5:89437963	CTD-2151A2.3	T	G	0,018	0,003	1.613e-09	0,011	0,008	0,140
rs670049	6:100087024	Y_RNA	A	C	0,019	0,003	6.417e-13	-0,002	0,007	0,720
rs9322822	6:105369598	LIN28B-AS1	C	T	0,015	0,003	1.349e-08	-0,005	0,007	0,470
rs9398171	6:108983527	FOXO3	T	C	0,050	0,003	9.510e-74	0,012	0,007	0,086
rs113127944	6:126656627	CENPW	A	G	0,051	0,008	6.115e-11	0,051	0,021	0,015
rs41285260	6:126661502	CENPW	T	G	0,039	0,004	5.123e-18	0,056	0,012	0,000
rs9321106	6:128355316	PTPRK	A	G	0,018	0,003	2.408e-08	-0,008	0,009	0,380
rs9398891	6:129314749	LAMA2	T	C	0,017	0,003	1.108e-10	0,000	0,007	0,980
rs3890746	6:130371055	L3MBTL3	C	T	0,020	0,003	1.060e-15	-0,001	0,006	0,860
rs7774230	6:152164239	ESR1	T	C	0,026	0,003	5.464e-25	-0,006	0,007	0,350
rs790513	6:154420368	OPRM1	C	A	0,025	0,003	1.575e-18	0,018	0,007	0,017
rs7758644	6:156583467	RP1-155D22.1	A	C	0,019	0,003	1.514e-08	-0,009	0,009	0,300
rs3127579	6:160674632	SLC22A2	A	G	0,033	0,004	1.230e-19	0,017	0,009	0,069
rs12110787	6:161474966	MAP3K4	A	C	0,022	0,004	1.857e-08	-0,004	0,010	0,720
rs3008051	6:166064315	PDE10A	C	T	0,014	0,003	1.946e-08	0,001	0,007	0,830
rs9364815	6:166197239	PDE10A	A	G	0,015	0,003	2.829e-08	-0,014	0,007	0,033
rs4709995	6:166313447	SDIM1	T	C	0,042	0,003	7.369e-60	0,002	0,007	0,820
rs2296198	6:18399750	RNF144B	C	T	0,016	0,003	1.937e-08	-0,015	0,007	0,041
rs73382439	6:20404420	E2F3	C	T	0,019	0,003	1.800e-08	-0,031	0,009	0,000
rs1165196	6:25813150	SLC17A1	G	A	0,029	0,003	6.414e-30	-0,011	0,006	0,070
rs1130838	6:31237124	HLA-C	C	T	0,026	0,003	1.848e-23	0,022	0,007	0,001
rs28362677	6:32362741	BTNL2	T	C	0,032	0,004	1.763e-19	-0,007	0,009	0,460
rs1042335	6:33052958	HLA-DPB1	T	C	0,015	0,003	3.568e-08	0,015	0,007	0,030
rs1150781	6:34214322	C6orf1	G	C	0,026	0,004	2.337e-09	-0,022	0,011	0,053
rs12194618	6:38091030	ZFAND3	A	G	0,017	0,003	2.686e-11	0,009	0,007	0,190
rs7740433	6:42908013	CNPY3	A	G	0,017	0,003	1.504e-08	0,001	0,007	0,910
rs998584	6:43757896	VEGFA	A	C	0,020	0,003	1.211e-15	0,041	0,007	0,000
rs6924225	6:45584732	RUNX2	G	A	0,019	0,003	1.442e-08	0,003	0,009	0,760
rs2397112	6:52684333	RP11-228O6.2	A	G	0,019	0,003	3.313e-13	0,019	0,006	0,003
rs584955	6:7097141	TMEM14C	A	G	0,036	0,006	2.579e-09	-0,031	0,016	0,051
rs6916994	6:87991236	GJB7	C	T	0,029	0,003	1.519e-31	0,003	0,006	0,690
rs7783012	7:114116881	FOXP2	G	A	0,016	0,003	1.561e-10	-0,010	0,006	0,120
rs12666306	7:115082406	RP11-222O23.1	G	A	0,017	0,003	1.702e-11	-0,009	0,006	0,170
rs7802508	7:1191689	ZFAND2A	A	G	0,021	0,003	9.477e-17	0,011	0,007	0,092
rs2896395	7:127511705	SND1	C	T	0,015	0,003	2.446e-08	-0,003	0,007	0,680
rs11556924	7:129663496	ZC3HC1	C	T	0,016	0,003	9.798e-10	0,014	0,007	0,028
rs207212	7:130547217	LINC00513	C	T	0,028	0,004	1.659e-11	-0,007	0,011	0,500
rs2048672	7:130653851	LINC-PINT	C	A	0,018	0,003	6.405e-11	0,013	0,007	0,065
rs1986692	7:133743393	EXOC4	G	A	0,015	0,003	8.640e-09	-0,006	0,007	0,380
rs273956	7:137603188	CREB3L2	A	G	0,021	0,003	5.369e-16	-0,018	0,006	0,004
rs4719393	7:14219213	DGKB	T	G	0,027	0,003	1.290e-22	0,009	0,007	0,210
rs114949263	7:150498245	TMEM176B	T	C	0,027	0,004	1.283e-11	0,015	0,010	0,150

rs10246481	7:156184748	AC073133.2	G	A	0,015	0,003	1.499e-08	-0,003	0,007	0,610
rs12699547	7:2015970	MAD1L1	C	T	0,021	0,003	2.438e-16	-0,008	0,007	0,230
rs1182174	7:2875420	GNA12	A	G	0,021	0,003	4.808e-14	-0,004	0,007	0,540
rs2228078	7:31018852	GHRHR	C	T	0,057	0,010	4.076e-08	-0,018	0,029	0,550
rs10252510	7:31023108	GHRHR	G	A	0,020	0,003	8.179e-14	0,007	0,007	0,330
rs1050327	7:44808017	ZMIZ2	A	G	0,017	0,003	2.907e-11	0,022	0,006	0,001
rs870796	7:45426435	ELK1P1	G	A	0,017	0,003	3.274e-11	0,015	0,006	0,018
rs79881512	7:45529127	AC073325.1	C	T	0,059	0,011	4.185e-08	0,044	0,028	0,110
rs2270628	7:45949570	IGFBP3	T	C	0,033	0,003	3.737e-25	0,002	0,008	0,790
rs145188037	7:45954465	IGFBP3	A	G	0,120	0,011	1.749e-27	-0,002	0,027	0,950
rs74657816	7:46670682	HMGN1P19	T	G	0,047	0,005	3.006e-18	0,015	0,014	0,280
rs2250243	7:6690240	ZNF316	C	T	0,024	0,003	1.694e-15	-0,008	0,007	0,310
rs35862187	7:69625029	AUTS2	A	G	0,031	0,006	3.484e-08	-0,036	0,014	0,010
rs17145738	7:72982874	TBL2	T	C	0,034	0,004	8.750e-19	0,036	0,010	0,000
rs411717	7:94033031	COL1A2	T	C	0,015	0,003	2.144e-09	-0,006	0,006	0,350
rs34312198	7:99674870	ZNF3	A	C	0,024	0,004	1.345e-09	-0,002	0,010	0,850
rs1786342	8:101676363	SNX31	T	C	0,017	0,003	1.241e-11	-0,002	0,007	0,750
rs9657541	8:10643164	CTD-2135J3.4	C	T	0,020	0,003	3.846e-10	0,002	0,008	0,770
rs60862542	8:109275071	EIF3E	G	A	0,017	0,003	1.648e-08	-0,005	0,007	0,490
rs2737205	8:116610180	TRPS1	T	C	0,023	0,003	4.711e-20	0,030	0,006	0,000
rs2978062	8:134571618	ST3GAL1	G	T	0,019	0,003	3.225e-08	0,022	0,009	0,010
rs716100	8:135661278	ZFAT	A	G	0,019	0,003	7.196e-13	0,019	0,007	0,006
rs12549853	8:145020636	PLEC	A	G	0,016	0,003	1.654e-09	-0,015	0,007	0,025
rs76393968	8:16282937	MSR1	G	A	0,060	0,010	2.566e-09	0,007	0,028	0,810
rs1495741	8:18272881	NAT2	G	A	0,026	0,003	5.034e-18	0,024	0,008	0,002
rs11782452	8:26361601	BNIP3L	G	A	0,015	0,003	1.258e-08	0,007	0,007	0,320
rs56352849	8:73769173	KCNB2	A	G	0,016	0,003	1.497e-08	0,007	0,007	0,310
rs1431015	8:77131580	RNU2-54P	C	T	0,020	0,003	2.655e-14	0,004	0,006	0,590
rs6473015	8:78178485	AC105242.1	C	A	0,019	0,003	2.994e-12	-0,011	0,007	0,120
rs445036	8:81408409	ZBTB10	T	C	0,019	0,003	5.794e-12	0,003	0,007	0,670
rs7034716	9:101858382	TGFBR1	C	T	0,015	0,003	2.595e-08	-0,007	0,008	0,330
rs7041137	9:110044572	RAD23B	T	C	0,017	0,003	7.298e-10	-0,013	0,007	0,074
rs7872812	9:119341544	ASTN2	T	C	0,026	0,004	3.162e-13	0,009	0,009	0,330
rs13301073	9:128284378	MAPKAP1	G	A	0,022	0,003	4.056e-17	0,012	0,007	0,069
rs10757291	9:22161884	CDKN2B-AS1	G	A	0,019	0,003	1.487e-14	0,001	0,006	0,910
rs10811787	9:22871816	RP11-370B11.4	C	T	0,015	0,003	7.833e-09	-0,013	0,006	0,036
rs11557154	9:34107505	DCAF12	T	C	0,024	0,004	3.100e-10	0,048	0,010	0,000
rs10869022	9:74057313	TRPM3	C	T	0,021	0,003	1.633e-11	-0,004	0,008	0,610
rs2378662	9:86707289	RP11-158D2.2	G	A	0,017	0,003	4.195e-11	-0,007	0,006	0,260
rs10908903	9:92228559	GADD45G	T	G	0,015	0,003	1.288e-09	-0,013	0,006	0,037
rs1055710	9:96214928	FAM120AOS	G	A	0,018	0,003	7.149e-12	0,003	0,007	0,630
rs28831479	9:98254526	PTCH1	C	A	0,022	0,003	1.196e-14	0,018	0,007	0,011
rs10509746	10:102656897	Y_RNA	C	T	0,027	0,003	1.943e-26	0,003	0,007	0,680
rs4917962	10:103931931	NOLC1	T	G	0,024	0,004	5.216e-10	0,014	0,010	0,140
rs12244851	10:114773926	TCF7L2	T	C	0,015	0,003	1.450e-08	0,270	0,007	0,000

rs3858325	10:117988795	GFRA1	T	C	0,019	0,003	1.713e-13	-0,004	0,006	0,520
rs2801482	10:12459773	CAMK1D	G	A	0,050	0,008	1.130e-09	0,007	0,020	0,720
rs7921105	10:13535398	BEND7	C	T	0,016	0,003	1.962e-10	0,019	0,006	0,004
rs11012712	10:21760015		C	T	0,022	0,003	3.752e-12	0,007	0,008	0,360
rs10047326	10:22839463	PIP4K2A	A	C	0,017	0,003	1.263e-10	0,003	0,007	0,660
rs1832007	10:5254847	AKR1C4	G	A	0,057	0,003	6.779e-60	-0,002	0,009	0,850
rs293275	10:53215020	PRKG1	C	T	0,014	0,003	1.603e-08	-0,005	0,006	0,430
rs10821713	10:62055781	ANK3	T	C	0,017	0,003	5.068e-11	-0,003	0,006	0,640
rs7910087	10:77209145	C10orf11	T	C	0,017	0,003	6.229e-12	0,017	0,006	0,007
rs4418728	10:94839724	CYP26A1	G	T	0,024	0,003	5.991e-21	-0,004	0,006	0,510
rs9630085	10:95333063	FFAR4	G	A	0,022	0,003	9.144e-13	-0,003	0,008	0,730
rs116454156	10:95347041	FFAR4	A	G	0,078	0,010	1.615e-14	0,046	0,025	0,068
rs2274224	10:96039597	PLCE1	G	C	0,024	0,003	1.017e-20	0,002	0,006	0,760
rs35023999	11:113266411	ANKK1	C	A	0,015	0,003	1.160e-09	-0,018	0,006	0,005
rs10892564	11:120224650	ARHGEF12	G	A	0,017	0,003	4.611e-11	0,003	0,007	0,670
rs4936759	11:122763516	C11orf63	C	T	0,016	0,003	1.949e-10	-0,013	0,006	0,039
rs10893499	11:126241979	ST3GAL4	G	A	0,022	0,004	3.842e-09	0,016	0,009	0,085
rs7947951	11:13356030	ARNTL	G	A	0,020	0,003	1.025e-13	0,018	0,007	0,010
rs61867536	11:1513700	MOB2	T	C	0,018	0,003	1.132e-12	0,005	0,006	0,440
rs72858776	11:15772953	RP11-396O20.2	G	T	0,030	0,005	1.479e-10	-0,006	0,012	0,580
rs11024614	11:18326758	HPS5	C	T	0,023	0,003	3.409e-18	0,006	0,007	0,400
rs7115466	11:2044150	H19	A	G	0,015	0,003	2.286e-08	-0,015	0,008	0,074
rs3213223	11:2156930	IGF2	A	G	0,076	0,003	9.35e-144	0,026	0,008	0,001
rs117600498	11:2297593	ASCL2	C	T	0,038	0,007	9.548e-09	-0,035	0,018	0,048
rs34452566	11:27793470	RP11-587D21.4	T	G	0,018	0,003	1.032e-08	0,004	0,008	0,590
rs11031058	11:30375889	RPL12P30	T	C	0,022	0,003	5.389e-11	-0,017	0,009	0,043
rs10767874	11:30776952	DCDC1	A	G	0,015	0,003	3.973e-08	-0,004	0,007	0,520
rs11029620	11:3771924	NUP98	C	T	0,022	0,003	4.620e-13	0,016	0,008	0,044
rs6485702	11:46898771	LRP4	T	C	0,017	0,003	1.415e-10	0,005	0,007	0,440
rs1039481	11:48182237	PTPRJ	G	A	0,042	0,003	4.113e-48	-0,006	0,007	0,430
rs146345029	11:59596007	GIF	A	G	0,034	0,006	1.886e-08	-0,006	0,016	0,720
rs174554	11:61579463	FADS1	A	G	0,022	0,003	7.202e-17	0,027	0,007	0,000
rs117104648	11:65543736	AP5B1	C	T	0,036	0,005	3.240e-12	-0,020	0,013	0,150
rs12790261	11:66988048	KDM2A	A	C	0,031	0,005	1.211e-11	-0,004	0,013	0,730
rs4980661	11:69306579	AP000439.3	A	G	0,014	0,003	8.689e-09	0,000	0,006	0,960
rs2512525	11:77923019	USP35	T	C	0,024	0,003	1.427e-12	0,013	0,009	0,130
rs67257872	11:8530218	STK33	A	G	0,014	0,003	2.315e-08	0,020	0,006	0,002
rs61904289	11:85994731	EED	T	C	0,016	0,003	1.392e-09	0,013	0,007	0,045
rs625245	11:94192103	MRE11	G	T	0,016	0,003	4.364e-09	-0,004	0,007	0,560
rs11111274	12:102838128	IGF1	G	A	0,080	0,003	7.59e-175	-0,021	0,007	0,004
rs10745954	12:103483094	RP11-328J6.1	G	A	0,015	0,003	1.690e-09	0,015	0,006	0,020
rs7314285	12:111522026	CUX2	G	T	0,052	0,005	2.043e-25	0,021	0,013	0,093
rs2460488	12:116187660	RP11-110L15.1	G	A	0,026	0,003	1.096e-14	-0,007	0,009	0,460
rs75938105	12:116261411	RP11-110L15.2	T	C	0,047	0,007	7.468e-12	-0,027	0,017	0,120
rs2856321	12:11855773	ETV6	G	A	0,026	0,003	1.583e-23	-0,002	0,007	0,790

rs1800574	12:121416864	HNF1A	T	C	0,145	0,007	3.641e-84	0,160	0,019	0,000
rs10841649	12:20954879	SLCO1B3	C	T	0,021	0,004	9.254e-09	-0,008	0,010	0,420
rs9738365	12:31997635	RP11-428G5.4	A	C	0,058	0,003	1.905e-92	0,016	0,007	0,028
rs3759302	12:32135186	KIAA1551	A	T	0,021	0,003	6.949e-11	0,004	0,008	0,600
rs12231073	12:38526901	RNA5SP358	T	G	0,017	0,003	5.421e-12	-0,010	0,006	0,130
rs11175935	12:40693806	LRRK2	G	T	0,020	0,003	5.189e-10	-0,010	0,008	0,200
rs247917	12:46265916	ARID2	T	C	0,015	0,003	1.322e-09	-0,021	0,006	0,001
rs117564283	12:52300110	ACVRL1	T	C	0,029	0,005	5.046e-09	-0,004	0,013	0,750
rs773116	12:56486159	ERBB3	G	A	0,016	0,003	1.265e-10	0,009	0,006	0,150
rs2657879	12:56865338	GLS2	A	G	0,020	0,003	9.621e-10	0,007	0,008	0,420
rs78607331	12:57648644	R3HDM2	C	T	0,037	0,006	6.059e-10	0,036	0,017	0,030
rs4547160	12:63503650	AVPR1A	T	G	0,018	0,003	3.168e-11	-0,010	0,007	0,120
rs1351394	12:66351826	HMGA2	C	T	0,024	0,003	6.924e-21	0,052	0,006	0,000
rs2230281	12:89917518	GALNT4	A	G	0,016	0,003	4.010e-09	-0,008	0,007	0,270
rs11064536	12:905582	WNK1	T	C	0,020	0,003	2.941e-09	0,008	0,009	0,330
rs12425869	12:93961353	SOCS2	A	G	0,018	0,003	3.957e-09	-0,020	0,008	0,009
rs10777540	12:94150321	CRADD	G	T	0,018	0,003	2.236e-12	0,003	0,006	0,660
rs10860237	12:98157010	RP11-1016B18.1	A	G	0,030	0,003	2.391e-28	0,006	0,007	0,370
rs71432868	13:106559402	SNORA25	C	T	0,028	0,005	3.105e-08	-0,014	0,014	0,300
rs9583151	13:107666257	AL354741.1	C	T	0,014	0,003	2.708e-08	0,011	0,006	0,100
rs7323205	13:110365525	LINC00676	C	T	0,015	0,003	9.354e-09	-0,008	0,007	0,220
rs6602909	13:114551993	GAS6	C	T	0,020	0,003	4.354e-14	-0,005	0,007	0,500
rs9532512	13:40769897	LINC00598	A	G	0,043	0,003	8.024e-40	-0,006	0,008	0,480
rs1170158	13:42701941	DGKH	T	G	0,021	0,003	1.041e-10	-0,021	0,008	0,009
rs1535793	13:47154966	LRCH1	A	G	0,024	0,003	7.422e-17	0,014	0,007	0,059
rs118081390	13:49671053	FNDC3A	G	A	0,028	0,005	1.353e-08	0,018	0,012	0,160
rs9573360	13:74771429	LINC00402	A	C	0,014	0,003	1.597e-08	0,007	0,006	0,300
rs10136874	14:101202022	DLK1	G	T	0,023	0,003	2.350e-20	-0,008	0,006	0,200
rs8017377	14:24883887	NYNRIN	G	A	0,017	0,003	3.353e-11	-0,013	0,006	0,047
rs28396553	14:36673392	AL162511.1	T	C	0,015	0,003	2.962e-09	-0,008	0,007	0,250
rs33912345	14:60976537	SIX6	C	A	0,023	0,003	2.363e-19	-0,018	0,007	0,006
rs79936318	14:64315556	SYNE2	A	G	0,017	0,003	3.875e-08	-0,013	0,008	0,130
rs36215895	14:64676751	SYNE2	C	T	0,082	0,013	2.121e-10	-0,051	0,036	0,150
rs168961	14:69282930	ZFP36L1	G	A	0,018	0,003	2.889e-13	0,002	0,006	0,810
rs17106640	14:69419751	ACTN1	G	A	0,017	0,003	8.431e-11	-0,011	0,007	0,092
rs13379043	14:74250126	ELMSAN1	T	C	0,025	0,003	5.776e-18	-0,004	0,007	0,570
rs175043	14:75471803	EIF2B2	G	A	0,018	0,003	1.834e-12	0,009	0,006	0,180
rs1061638	14:77928525	AHSA1	G	A	0,018	0,003	7.484e-11	0,028	0,007	0,000
rs10145154	14:79939525	NRXN3	T	C	0,018	0,003	9.924e-10	0,057	0,008	0,000
rs78598185	14:92791479	SLC24A4	G	A	0,029	0,004	7.090e-11	0,008	0,011	0,470
rs1115897	14:93910816	UNC79	A	C	0,021	0,003	1.540e-14	-0,003	0,007	0,660
rs28929474	14:94844947	SERPINA1	C	T	0,063	0,009	1.294e-12	0,110	0,025	0,000
rs17747633	15:40916237	KNL1	G	A	0,015	0,003	3.080e-09	0,000	0,006	0,990
rs55707100	15:43820717	MAP1A	C	T	0,151	0,008	1.406e-76	0,051	0,020	0,012
rs4545755	15:51549044	CYP19A1	G	A	0,016	0,003	3.865e-10	0,009	0,006	0,170

rs12442867	15:62489128	RP11-299H22.1	A	C	0,017	0,003	9.329e-11	0,010	0,007	0,150
rs2004839	15:62539992	RP11-299H22.3	G	A	0,021	0,003	4.336e-10	-0,012	0,009	0,180
rs79076440	15:63803863	USP3	A	G	0,019	0,003	1.164e-08	-0,030	0,008	0,000
rs8033075	15:68353652	PIAS1	A	G	0,045	0,005	1.061e-17	-0,007	0,014	0,620
rs5742915	15:74336633	PML	C	T	0,025	0,003	1.723e-22	-0,022	0,007	0,001
rs2930313	15:74609378	CCDC33	G	A	0,028	0,005	4.141e-09	-0,010	0,012	0,400
rs12593755	15:89111712	RP11-97O12.3	T	G	0,016	0,003	1.846e-09	0,005	0,006	0,470
rs11856160	15:93452846	CHD2	A	G	0,021	0,003	2.843e-09	0,008	0,009	0,370
rs12912439	15:95828705	LINC01197	T	C	0,022	0,003	1.246e-15	0,005	0,007	0,500
rs2311313	15:99178274	RP11-35O15.1	G	T	0,019	0,003	1.691e-08	0,024	0,009	0,006
rs142354201	15:99524022	PGPEP1L	G	A	0,034	0,006	3.677e-09	0,004	0,015	0,810
rs1532824	16:10532211	ATF7IP2	A	C	0,017	0,003	4.521e-09	-0,001	0,007	0,860
rs4988483	16:1129010	SSTR5	C	A	0,172	0,006	1.87e-203	-0,079	0,016	0,000
rs8054054	16:1488646	CCDC154	G	A	0,015	0,003	1.026e-09	0,013	0,007	0,054
rs4786350	16:1638000	IFT140	C	G	0,036	0,006	6.048e-09	0,005	0,017	0,760
rs12935465	16:17476853	XYLT1	T	C	0,016	0,003	5.769e-11	-0,013	0,006	0,037
rs72761177	16:1833508	NUBP2	A	G	0,077	0,004	7.205e-70	-0,028	0,012	0,017
rs80253441	16:1842815	IGFALS	T	C	0,131	0,011	8.227e-36	-0,001	0,027	0,970
rs1657125	16:1912021	MEIOB	T	G	0,032	0,004	2.007e-18	0,004	0,009	0,670
rs2023762	16:19276597	SYT17	T	C	0,015	0,003	5.991e-09	0,000	0,006	0,960
rs12927172	16:27325021	IL4R	A	G	0,015	0,003	3.066e-09	-0,007	0,007	0,320
rs7498665	16:28883241	SH2B1	G	A	0,019	0,003	7.173e-14	0,029	0,007	0,000
rs4788220	16:30063780	FAM57B	G	A	0,017	0,003	5.263e-12	0,035	0,006	0,000
rs750952	16:31093954	ZNF646	C	T	0,032	0,003	1.105e-34	-0,010	0,007	0,140
rs11077337	16:3492048	ZNF597	T	G	0,015	0,003	1.454e-09	0,003	0,006	0,630
rs61731445	16:4016377		C	T	0,028	0,005	1.958e-08	-0,028	0,013	0,035
rs8182173	16:4420787	CORO7-PAM16	T	C	0,018	0,003	1.610e-09	0,024	0,007	0,002
rs116971887	16:51170026	SALL1	G	T	0,036	0,006	4.895e-09	-0,013	0,016	0,430
rs12597502	16:53170069	CHD9	G	A	0,015	0,003	4.528e-08	-0,009	0,007	0,200
rs1548917	16:56109333	RP11-461O7.1	T	C	0,015	0,003	2.361e-09	-0,021	0,006	0,001
rs74774288	16:5922263	RP11-420N3.3	G	T	0,027	0,003	2.955e-17	0,005	0,008	0,570
rs111792934	16:69131293	HAS3	C	T	0,022	0,003	1.430e-10	0,008	0,008	0,330
rs17299478	16:69775500	NOB1	C	T	0,032	0,003	2.497e-21	-0,037	0,009	0,000
rs12935091	16:71525208	ZNF19	G	A	0,035	0,006	2.235e-09	-0,005	0,015	0,760
rs147491123	16:72567795	LINC01572	C	T	0,036	0,007	3.286e-08	-0,036	0,017	0,031
rs8059803	16:81603001	CMIP	A	G	0,031	0,003	4.415e-29	0,023	0,007	0,001
rs753108	16:81728981	CMIP	A	G	0,025	0,003	1.606e-17	-0,026	0,007	0,000
rs11149612	16:83980965	RP11-505K9.4	C	T	0,027	0,003	1.131e-26	-0,026	0,007	0,000
rs8054322	16:85201405	GSE1	A	G	0,015	0,003	4.723e-09	0,008	0,007	0,210
rs4985062	16:8996636	USP7	T	C	0,015	0,003	2.040e-09	0,014	0,007	0,038
rs143076454	16:921179	LMF1	G	A	0,072	0,009	1.245e-14	0,003	0,027	0,930
rs7204824	16:969631	LMF1	C	T	0,024	0,003	1.168e-16	0,025	0,007	0,001
rs6416868	17:15924370	TTC19	A	G	0,019	0,003	3.115e-14	-0,008	0,006	0,210
rs7502910	17:1638718	WDR81	A	G	0,016	0,003	1.528e-10	0,001	0,007	0,940
rs8075153	17:17622666	RAI1	C	T	0,021	0,003	1.831e-17	0,024	0,006	0,000

rs8079923	17:19869544	AKAP10	C	T	0,016	0,003	3.664e-08	0,015	0,007	0,035
rs56030650	17:38131187	GSDMA	C	A	0,022	0,003	2.417e-18	-0,004	0,006	0,520
rs668799	17:40716235	COASY	C	T	0,018	0,003	4.019e-10	-0,048	0,007	0,000
rs199525	17:44847834	WNT3	G	T	0,020	0,003	5.613e-11	0,013	0,008	0,100
rs2309401	17:5471902	NLRP1	T	G	0,015	0,003	6.785e-09	0,010	0,007	0,140
rs35819807	17:61623052	KCNH6	T	C	0,017	0,003	2.387e-09	-0,035	0,007	0,000
rs142377191	17:61649170	DCAF7	A	G	0,125	0,009	2.048e-46	0,031	0,024	0,190
rs3760237	17:62051110	SCN4A	C	T	0,024	0,003	1.441e-21	-0,004	0,006	0,560
rs76708468	17:62206299	1ERN	C	T	0,087	0,006	3.084e-41	0,008	0,019	0,670
rs1801689	17:64210580	APOH	C	A	0,089	0,007	1.248e-33	0,017	0,020	0,400
rs77542162	17:67081278	ABCA6	G	A	0,054	0,009	2.412e-10	-0,055	0,024	0,019
rs6501601	17:71124903	POLR3KP2	G	A	0,015	0,003	4.071e-09	-0,019	0,007	0,004
rs4789227	17:73794354	UNK	T	C	0,015	0,003	4.335e-09	0,007	0,007	0,280
rs9892862	17:7439014	Y_RNA	G	A	0,022	0,003	9.705e-14	-0,028	0,008	0,000
rs4075483	17:79074817	BAIAP2	C	T	0,017	0,003	1.312e-10	0,014	0,007	0,038
rs8095538	18:1616505	-	G	T	0,020	0,003	8.447e-14	-0,011	0,007	0,130
rs8084351	18:50726559	DCC	G	A	0,015	0,003	8.537e-10	-0,005	0,006	0,410
rs11152071	18:56087417	RP11-1151B14.2	C	T	0,020	0,003	8.957e-12	-0,010	0,007	0,160
rs190102446	18:57048571	RP11-27G24.1	C	T	0,041	0,007	2.452e-09	0,037	0,017	0,030
rs585187	18:58177124	MRPS5P4	T	G	0,015	0,003	3.712e-09	-0,013	0,006	0,033
rs12454712	18:60845884	BCL2	T	C	0,018	0,003	4.051e-12	0,049	0,007	0,000
rs8097893	18:74983055	GALR1	A	G	0,058	0,006	1.723e-20	0,006	0,015	0,690
rs57551555	18:75209278	RP11-176N18.2	T	G	0,019	0,003	5.817e-13	0,000	0,007	0,960
rs8105174	19:10347032	DNMT1	C	T	0,050	0,003	7.971e-54	-0,008	0,009	0,340
rs34536443	19:10463118	TYK2	G	C	0,045	0,006	9.964e-14	0,035	0,017	0,039
rs8113618	19:10816055	QTRT1	T	C	0,031	0,003	1.185e-33	0,000	0,006	0,980
rs6510033	19:30710785	AC005597.1	A	G	0,020	0,003	1.147e-12	-0,001	0,007	0,860
rs58658292	19:30938425	ZNF536	G	A	0,030	0,006	4.618e-08	0,004	0,014	0,800
rs6510177	19:31211647	ZNF536	T	C	0,023	0,003	1.598e-12	-0,005	0,008	0,520
rs62102136	19:34700561	LSM14A	C	T	0,016	0,003	6.527e-09	-0,001	0,007	0,840
rs7254601	19:36147315	COX6B1	G	A	0,016	0,003	2.551e-08	0,003	0,008	0,710
rs58560372	19:38758752	SPINT2	C	T	0,020	0,003	1.331e-08	-0,003	0,009	0,770
rs67868323	19:4048561	ZBTB7A	T	G	0,016	0,003	2.205e-08	0,004	0,007	0,600
rs11671304	19:47564643	ZC3H4	C	T	0,018	0,003	6.884e-11	-0,044	0,007	0,000
rs3760954	19:4772343	MIR7-3HG	T	C	0,024	0,004	2.909e-10	-0,010	0,010	0,310
rs296361	19:48389363	SULT2A1	A	G	0,025	0,003	3.421e-13	0,005	0,009	0,580
rs2287922	19:49232226	RASIP1	G	A	0,030	0,003	1.833e-33	-0,004	0,006	0,560
rs6510832	19:4967954	KDM4B	G	T	0,033	0,005	2.638e-10	-0,003	0,013	0,840
rs7256521	19:53837110	ZNF845	G	A	0,015	0,003	9.761e-10	0,004	0,007	0,510
rs12975366	19:54759361	LILRB5	T	C	0,020	0,003	2.253e-15	0,000	0,007	0,980
rs8112883	19:7179320	INSR	G	T	0,017	0,003	1.166e-09	0,007	0,007	0,330
rs7267595	20:10643850	JAG1	A	C	0,015	0,003	9.437e-10	0,005	0,006	0,480
rs7508949	20:20033367	CRNKL1	C	G	0,025	0,003	1.490e-22	0,014	0,006	0,031
rs6046825	20:20368925	RALGAPA2	A	C	0,024	0,003	2.108e-17	-0,007	0,007	0,370
rs75989562	20:21012077	APMAP	A	G	0,030	0,005	8.093e-09	0,024	0,014	0,088

rs2424396	20:21630280	LINC01726	G	A	0,033	0,004	2.245e-14	-0,005	0,011	0,640
rs6037508	20:3217989	SLC4A11	G	T	0,017	0,003	8.740e-09	0,007	0,007	0,310
rs6088579	20:33284624	NCOA6	G	A	0,027	0,003	8.509e-17	0,013	0,008	0,120
rs2207132	20:39142516	LINC01728	G	A	0,048	0,007	1.064e-11	-0,017	0,018	0,340
rs17265513	20:39832628	ZHX3	T	C	0,022	0,003	3.454e-12	-0,024	0,008	0,002
rs16995311	20:49201102	PTPN1	A	C	0,040	0,005	5.559e-18	-0,011	0,012	0,340
rs2738787	20:62328375	TNFRSF6B	A	G	0,037	0,005	1.251e-15	-0,021	0,012	0,088
rs4809401	20:62737568	NPBWR2	T	C	0,023	0,004	6.488e-10	-0,029	0,010	0,004
rs9978775	21:40694526	BRWD1-AS1	G	A	0,019	0,003	2.351e-13	0,014	0,006	0,033
rs8138950	22:29448643	ZNRF3	C	T	0,015	0,003	1.323e-09	-0,003	0,006	0,660
rs2412973	22:30529631	HORMAD2	A	C	0,014	0,003	3.334e-08	0,001	0,006	0,940
rs12106594	22:31885316	EIF4ENIF1	T	C	0,036	0,006	1.588e-09	-0,004	0,014	0,780
rs5755948	22:36179095	RBFOX2	A	G	0,028	0,004	4.375e-14	0,002	0,010	0,870
rs6519133	22:39096602	JOSD1	T	C	0,029	0,003	7.731e-30	0,008	0,007	0,210
rs9611565	22:41767486	TEF	T	C	0,029	0,003	3.825e-23	-0,019	0,007	0,013
rs4823324	22:46238123	ATXN10	T	C	0,016	0,003	1.938e-10	-0,006	0,007	0,340

Chr, chromosome; EA, effect allele; OA, other allele; SE, standard error.

ESM Table 2. Summary statistics of the single-nucleotide polymorphisms associated with insulin-like growth factor-1 and their associations with coronary artery disease

					IGF-1			Coronary artery disease		
SNP	Chr	Nearby gene	EA	OA	Beta	SE	*p*	Beta	SE	*p*
rs10159299	1	PLXNA2	C	T	0,018	0,003	5.112e-12	0,016	0,009	0,085
rs1046011	1	LEPROT	T	C	0,021	0,003	3.303e-14	-0,002	0,010	0,864
rs10779509	1	RP1-272L16.1	C	T	0,014	0,003	2.109e-08	-0,001	0,009	0,887
rs11165778	1	HFM1	A	G	0,016	0,003	1.492e-08	-0,007	0,012	0,534
rs112436634	1	PEX14	T	C	0,016	0,003	1.749e-09	0,007	0,010	0,471
rs1127313	1	ADAR	G	A	0,024	0,003	1.394e-21	0,025	0,009	0,007
rs11577063	1	AXDND1	T	G	0,020	0,003	1.511e-11	-0,008	0,011	0,449
rs12141189	1	HLX	T	C	0,045	0,003	4.135e-53	0,009	0,011	0,425
rs1223763	1	RP11-53A1.3	T	G	0,024	0,003	8.461e-13	0,008	0,012	0,499
rs12723255	1	EIF4G3	C	T	0,017	0,003	3.229e-11	-0,002	0,009	0,827
rs12749024	1	PAPPA2	T	C	0,075	0,004	7.72e-100	-0,007	0,014	0,627
rs140604451	1	GSTM2	G	A	0,054	0,008	3.524e-11	0,029	0,033	0,388
rs1430753	1	WLS	A	G	0,021	0,003	7.085e-11	-0,020	0,012	0,101
rs143885630	1	SMG7	G	A	0,030	0,004	1.358e-15	0,006	0,014	0,660
rs165316	1	RPL5P6	G	A	0,073	0,003	9.66e-119	0,022	0,012	0,073
rs17037452	1	CLCN6	A	G	0,023	0,003	1.658e-11	0,024	0,013	0,061
rs17393144	1	MIR34AHG	A	G	0,016	0,003	9.319e-09	0,014	0,011	0,188
rs1825813	1	C1orf146	A	G	0,023	0,003	1.879e-13	0,026	0,013	0,050
rs2075995	1	E2F2	C	A	0,014	0,003	9.790e-09	0,004	0,009	0,662
rs2724373	1	C1orf132	C	T	0,019	0,003	1.436e-12	-0,011	0,010	0,277
rs2802330	1	PDIK1L	G	A	0,031	0,003	4.632e-21	-0,006	0,013	0,659
rs2802951	1	RN7SL668P	A	G	0,016	0,003	4.970e-09	-0,011	0,011	0,297
rs2819336	1	PTPRF	T	C	0,027	0,003	1.550e-25	-0,001	0,010	0,894
rs3131646	1	MYCL	G	T	0,016	0,003	1.710e-08	0,007	0,010	0,486
rs36086195	1	ARHGEF19-AS1	C	T	0,019	0,003	1.359e-13	-0,026	0,010	0,007
rs4306136	1	RP11-103C3.1	A	G	0,017	0,003	1.997e-11	-0,015	0,010	0,130
rs569356	1	OPRD1	G	A	0,027	0,004	5.953e-14	-0,002	0,015	0,918
rs599839	1	CELSR2	G	A	0,031	0,003	9.629e-26	-0,096	0,011	0,000
rs61780439	1	SLFNL1	G	A	0,021	0,003	5.323e-12	-0,011	0,012	0,367
rs6659176	1	NR0B2	C	G	0,042	0,005	1.354e-19	-0,015	0,019	0,415
rs6701954	1	USP48	T	G	0,014	0,003	3.497e-08	-0,009	0,010	0,380
rs684818	1	RP4-781K5.7	T	C	0,024	0,003	9.658e-21	0,021	0,009	0,028
rs708108	1	WNT3A	T	C	0,015	0,003	5.872e-09	0,010	0,010	0,327
rs7517340	1	AKT3	C	T	0,035	0,003	2.123e-26	0,005	0,012	0,682
rs7528548	1	PYGO2	C	T	0,054	0,009	7.954e-10	-0,006	0,026	0,811
rs7539178	1	JAK1	C	A	0,026	0,004	1.365e-12	0,010	0,012	0,403
rs7545345	1	NUCKS1	C	T	0,026	0,004	1.032e-12	-0,009	0,015	0,528
rs75681856	1	RABGAP1L	T	C	0,023	0,004	5.171e-09	0,004	0,014	0,749
rs75907879	1	SPEN	T	C	0,024	0,004	1.425e-10	0,012	0,022	0,592
rs77369503	1	RGS4	G	A	0,045	0,007	1.397e-10	0,020	0,036	0,584

rs903908	1	SKI	C	T	0,016	0,003	2.176e-10	-0,021	0,010	0,032
rs11677980	2	LBH	G	A	0,015	0,003	3.043e-08	-0,002	0,011	0,840
rs11678946	2	EPHA4	A	C	0,014	0,003	3.143e-08	-0,008	0,010	0,422
rs12471768	2	SERTAD2	C	T	0,022	0,003	1.639e-15	0,003	0,011	0,812
rs1260326	2	GCKR	C	T	0,063	0,003	9.66e-133	0,003	0,010	0,735
rs12710648	2	SMC6	A	G	0,017	0,003	5.703e-12	-0,009	0,010	0,387
rs1465529	2	SP110	T	C	0,019	0,003	1.386e-12	-0,009	0,011	0,395
rs17050272	2	AC073257.2	G	A	0,024	0,003	3.201e-20	0,006	0,010	0,560
rs17323117	2	NEU2	G	A	0,029	0,005	7.980e-10	0,001	0,020	0,963
rs17400325	2	PDE11A	C	T	0,054	0,006	9.393e-17	-0,021	0,024	0,397
rs2674492	2	CYBRD1	A	G	0,014	0,003	4.835e-08	0,013	0,010	0,206
rs35135518	2	AC010145.4	T	C	0,029	0,004	1.521e-12	0,003	0,016	0,851
rs35641591	2	PCBP1-AS1	C	T	0,050	0,006	1.575e-14	0,036	0,041	0,383
rs3791679	2	EFEMP1	G	A	0,018	0,003	4.338e-09	0,001	0,011	0,960
rs4402747	2	NEU2	A	G	0,016	0,003	2.960e-10	-0,004	0,009	0,681
rs58387407	2	CACNB4	G	A	0,018	0,003	3.502e-08	-0,005	0,011	0,654
rs62136965	2	RNU6-566P	C	T	0,037	0,006	6.429e-10	0,019	0,024	0,428
rs62182127	2	VIL1	A	G	0,019	0,003	2.712e-14	0,003	0,010	0,800
rs6435156	2	BMPR2	C	T	0,024	0,003	2.045e-17	0,010	0,011	0,345
rs6437249	2	HDLBP	C	T	0,019	0,003	4.559e-12	0,015	0,011	0,164
rs6544549	2	KCNG3	T	C	0,024	0,004	1.592e-10	-0,006	0,014	0,682
rs6749680	2	ALMS1	A	G	0,015	0,003	9.868e-09	-0,014	0,010	0,147
rs6760135	2	ASXL2	T	C	0,050	0,003	1.194e-63	0,018	0,012	0,130
rs702878	2	AC074391.1	A	G	0,014	0,003	1.815e-08	0,011	0,010	0,274
rs73954943	2	BCL2L11	A	G	0,031	0,005	1.063e-09	0,045	0,019	0,017
rs7574340	2	SLC8A1	T	C	0,017	0,003	2.971e-10	0,000	0,010	0,979
rs7578633	2	PAX8	T	C	0,018	0,003	3.379e-12	0,007	0,010	0,468
rs112893170	3	FEZF2	T	C	0,020	0,003	1.527e-10	0,004	0,013	0,777
rs11717397	3	UBE2E2	G	A	0,015	0,003	2.572e-09	-0,003	0,009	0,756
rs11928797	3	UBP1	A	C	0,030	0,004	2.428e-14	-0,007	0,014	0,617
rs12491473	3	CCDC12	G	A	0,020	0,003	5.913e-15	-0,001	0,009	0,953
rs13069961	3	KALRN	G	A	0,018	0,003	3.680e-09	-0,005	0,012	0,693
rs13073970	3	EIF5A2	T	G	0,025	0,003	2.016e-15	-0,021	0,012	0,073
rs1822825	3	PPARG	G	A	0,014	0,003	2.886e-08	0,015	0,009	0,098
rs2268829	3	DGKG	G	A	0,018	0,003	3.038e-09	0,005	0,011	0,644
rs2607748	3	CHCHD4	C	T	0,017	0,003	3.289e-11	0,016	0,009	0,082
rs3772102	3	ST3GAL6	G	T	0,020	0,003	5.954e-16	0,000	0,009	0,969
rs4678497	3	SUSD5	C	T	0,017	0,003	1.959e-10	0,010	0,009	0,268
rs504603	3	BZW1P1	C	T	0,029	0,005	2.402e-09	-0,030	0,017	0,079
rs55717031	3	MRPS22	G	T	0,032	0,003	2.474e-31	-0,015	0,011	0,170
rs56062334	3	LINC02068	T	C	0,017	0,003	1.839e-11	0,007	0,010	0,465
rs62263345	3	BBX	A	G	0,028	0,004	1.640e-14	-0,008	0,016	0,630
rs62280667	3	SENP7	C	T	0,028	0,003	2.477e-26	-0,011	0,010	0,257
rs6440008	3	ZBTB38	T	C	0,035	0,003	1.113e-41	0,017	0,010	0,094
rs66707192	3	HRG	G	A	0,018	0,003	4.415e-10	0,010	0,010	0,308

rs687339	3	KRT18P35	T	C	0,040	0,003	2.666e-40	0,038	0,011	0,001
rs73238159	3	XRN1	C	T	0,025	0,004	1.059e-11	-0,012	0,014	0,401
rs7625680	3	ATG7	A	G	0,015	0,003	7.693e-09	0,016	0,009	0,093
rs7628689	3	C3orf38	G	A	0,029	0,003	6.825e-17	0,010	0,013	0,469
rs811332	3	MRAS	C	T	0,019	0,003	2.011e-09	-0,001	0,012	0,964
rs9819762	3	PIK3CA	T	C	0,019	0,003	1.212e-08	-0,011	0,012	0,388
rs1055582	4	UBE2K	C	T	0,027	0,003	1.578e-27	-0,005	0,009	0,600
rs111443396	4	LINC01091	C	T	0,026	0,004	2.015e-10	0,014	0,015	0,379
rs1229984	4	ADH1B	T	C	0,104	0,009	1.219e-34	-0,033	0,019	0,075
rs13108218	4	HGFAC	G	A	0,017	0,003	5.920e-11	-0,010	0,010	0,322
rs17429745	4	RP11-556I14.1	G	T	0,026	0,003	2.045e-21	0,003	0,010	0,732
rs2280099	4	TIGD2	G	A	0,025	0,003	1.923e-14	0,002	0,012	0,880
rs35036084	4	RP11-145G20.1	T	C	0,017	0,003	8.218e-11	0,002	0,010	0,819
rs3804173	4	PRDM5	G	A	0,020	0,003	4.484e-13	-0,019	0,010	0,057
rs4394044	4	SORBS2	T	C	0,014	0,003	4.708e-08	-0,010	0,009	0,278
rs62302688	4	GABRA2	G	A	0,039	0,004	4.057e-20	0,029	0,020	0,163
rs62334147	4	DDX60L	C	T	0,019	0,003	4.261e-09	0,012	0,012	0,323
rs62342064	4	RP11-119H12.3	C	T	0,022	0,004	1.836e-09	0,004	0,017	0,820
rs6532798	4	ADH4	T	C	0,037	0,003	1.364e-41	0,002	0,011	0,821
rs6827641	4	HHIP	T	C	0,014	0,003	2.623e-08	-0,005	0,009	0,617
rs6853741	4	ARHGAP10	A	G	0,024	0,003	1.676e-16	0,011	0,011	0,282
rs7667562	4	LARP1B	C	A	0,016	0,003	3.770e-09	-0,018	0,010	0,087
rs11242236	5	C5orf66	G	A	0,025	0,003	1.957e-22	0,001	0,009	0,923
rs11954036	5	PDE4D	T	C	0,037	0,003	1.936e-44	0,013	0,010	0,182
rs12108803	5	TBCA	G	T	0,033	0,006	1.015e-08	-0,010	0,022	0,646
rs12520263	5	RP11-357F12.1	T	G	0,017	0,003	2.579e-09	-0,008	0,010	0,434
rs13168379	5	CPEB4	A	G	0,031	0,005	6.148e-10	-0,006	0,015	0,662
rs13178887	5	MEF2C-AS1	T	C	0,023	0,003	2.712e-19	-0,001	0,010	0,957
rs1498603	5	PDE4D	T	G	0,031	0,005	1.170e-09	-0,004	0,019	0,851
rs17714046	5	TRIM41	C	T	0,042	0,006	1.197e-12	0,040	0,027	0,139
rs2042253	5	MIR5197	T	C	0,023	0,003	1.017e-14	-0,026	0,010	0,013
rs2227819	5	F2R	T	C	0,022	0,004	4.322e-08	0,001	0,017	0,945
rs2366398	5	CTD-2151A2.3	T	G	0,018	0,003	1.613e-09	0,018	0,012	0,128
rs258775	5	ARHGAP26	A	C	0,025	0,003	3.394e-14	0,004	0,012	0,761
rs26822	5	PPIP5K2	G	A	0,017	0,003	9.845e-11	-0,008	0,011	0,460
rs28650790	5	C5orf67	T	C	0,018	0,003	4.108e-08	0,045	0,012	0,000
rs329122	5	JADE2	A	G	0,018	0,003	1.312e-12	0,021	0,009	0,027
rs35668185	5	SLIT3	T	C	0,056	0,003	3.402e-73	0,007	0,011	0,540
rs3734166	5	CDC25C	A	G	0,028	0,003	8.063e-22	0,000	0,010	0,977
rs6180	5	GHR	A	C	0,035	0,003	6.019e-43	-0,014	0,009	0,130
rs6895953	5	RP11-357F12.1	G	A	0,024	0,003	5.167e-21	0,010	0,009	0,283
rs72758321	5	PLCXD3	G	A	0,047	0,006	3.926e-15	-0,084	0,032	0,008
rs73271090	5	CTB-1I21.1	G	A	0,044	0,003	7.844e-39	-0,002	0,013	0,894
rs7719168	5	15ARL	C	A	0,030	0,004	2.429e-14	0,055	0,016	0,001
rs80170948	5	SREK1IP1	G	T	0,039	0,006	2.221e-09	0,012	0,030	0,675

rs840809	5	CTD-2232E5.2	A	C	0,016	0,003	1.183e-08	0,021	0,010	0,047
rs9292578	5	PRLR	C	A	0,040	0,006	3.195e-10	0,008	0,024	0,732
rs113127944	6	CENPW	A	G	0,051	0,008	6.115e-11	-0,026	0,038	0,503
rs1150781	6	C6orf1	G	C	0,026	0,004	2.337e-09	-0,010	0,017	0,546
rs1165196	6	SLC17A1	G	A	0,029	0,003	6.414e-30	-0,014	0,009	0,138
rs12110787	6	MAP3K4	A	C	0,022	0,004	1.857e-08	-0,019	0,013	0,136
rs12194618	6	ZFAND3	A	G	0,017	0,003	2.686e-11	0,004	0,010	0,659
rs2296198	6	RNF144B	C	T	0,016	0,003	1.937e-08	0,016	0,011	0,132
rs2397112	6	RP11-228O6.2	A	G	0,019	0,003	3.313e-13	0,000	0,010	0,978
rs3008051	6	PDE10A	C	T	0,014	0,003	1.946e-08	0,007	0,010	0,475
rs3127579	6	SLC22A2	A	G	0,033	0,004	1.230e-19	0,048	0,014	0,001
rs3890746	6	L3MBTL3	C	T	0,020	0,003	1.060e-15	-0,005	0,009	0,586
rs41285260	6	CENPW	T	G	0,039	0,004	5.123e-18	-0,006	0,019	0,738
rs4709995	6	SDIM1	T	C	0,042	0,003	7.369e-60	-0,015	0,009	0,106
rs584955	6	TMEM14C	A	G	0,036	0,006	2.579e-09	0,004	0,023	0,849
rs670049	6	Y_RNA	A	C	0,019	0,003	6.417e-13	0,011	0,010	0,284
rs6916994	6	GJB7	C	T	0,029	0,003	1.519e-31	0,006	0,009	0,486
rs6924225	6	RUNX2	G	A	0,019	0,003	1.442e-08	0,014	0,013	0,256
rs73382439	6	E2F3	C	T	0,019	0,003	1.800e-08	-0,001	0,011	0,933
rs7740433	6	CNPY3	A	G	0,017	0,003	1.504e-08	0,007	0,011	0,547
rs7758644	6	RP1-155D22.1	A	C	0,019	0,003	1.514e-08	0,008	0,013	0,549
rs7774230	6	ESR1	T	C	0,026	0,003	5.464e-25	0,009	0,009	0,343
rs790513	6	OPRM1	C	A	0,025	0,003	1.575e-18	0,012	0,011	0,274
rs9321106	6	PTPRK	A	G	0,018	0,003	2.408e-08	0,012	0,014	0,386
rs9322822	6	LIN28B-AS1	C	T	0,015	0,003	1.349e-08	0,012	0,010	0,235
rs9364815	6	PDE10A	A	G	0,015	0,003	2.829e-08	-0,007	0,010	0,479
rs9398171	6	FOXO3	T	C	0,050	0,003	9.510e-74	0,005	0,010	0,596
rs9398891	6	LAMA2	T	C	0,017	0,003	1.108e-10	0,007	0,010	0,488
rs998584	6	VEGFA	A	C	0,020	0,003	1.211e-15	0,042	0,010	0,000
rs10246481	7	AC073133.2	G	A	0,015	0,003	1.499e-08	0,022	0,009	0,018
rs10252510	7	GHRHR	G	A	0,020	0,003	8.179e-14	0,005	0,011	0,648
rs1050327	7	ZMIZ2	A	G	0,017	0,003	2.907e-11	0,010	0,009	0,301
rs114949263	7	TMEM176B	T	C	0,027	0,004	1.283e-11	0,025	0,017	0,158
rs11556924	7	ZC3HC1	C	T	0,016	0,003	9.798e-10	0,073	0,011	0,000
rs1182174	7	GNA12	A	G	0,021	0,003	4.808e-14	-0,005	0,011	0,658
rs12666306	7	RP11-222O23.1	G	A	0,017	0,003	1.702e-11	-0,005	0,009	0,604
rs12699547	7	MAD1L1	C	T	0,021	0,003	2.438e-16	-0,024	0,010	0,015
rs145188037	7	IGFBP3	A	G	0,120	0,011	1.749e-27	-0,005	0,049	0,925
rs17145738	7	TBL2	T	C	0,034	0,004	8.750e-19	0,008	0,015	0,591
rs1986692	7	EXOC4	G	A	0,015	0,003	8.640e-09	-0,002	0,010	0,831
rs2048672	7	LINC-PINT	C	A	0,018	0,003	6.405e-11	-0,004	0,010	0,650
rs207212	7	LINC00513	C	T	0,028	0,004	1.659e-11	-0,011	0,022	0,628
rs2228078	7	GHRHR	C	T	0,057	0,010	4.076e-08	0,036	0,045	0,425
rs2250243	7	ZNF316	C	T	0,024	0,003	1.694e-15	0,000	0,010	0,999
rs2270628	7	IGFBP3	T	C	0,033	0,003	3.737e-25	-0,016	0,012	0,194

rs273956	7	CREB3L2	A	G	0,021	0,003	5.369e-16	0,003	0,010	0,746
rs2896395	7	SND1	C	T	0,015	0,003	2.446e-08	0,011	0,010	0,257
rs34312198	7	ZNF3	A	C	0,024	0,004	1.345e-09	-0,002	0,017	0,891
rs35862187	7	AUTS2	A	G	0,031	0,006	3.484e-08	-0,016	0,023	0,500
rs411717	7	COL1A2	T	C	0,015	0,003	2.144e-09	0,000	0,009	0,960
rs4719393	7	DGKB	T	G	0,027	0,003	1.290e-22	0,002	0,010	0,830
rs74657816	7	HMGN1P19	T	G	0,047	0,005	3.006e-18	-0,015	0,022	0,488
rs7783012	7	FOXP2	G	A	0,016	0,003	1.561e-10	-0,020	0,009	0,031
rs7802508	7	ZFAND2A	A	G	0,021	0,003	9.477e-17	-0,006	0,010	0,507
rs79881512	7	AC073325.1	C	T	0,059	0,011	4.185e-08	-0,028	0,034	0,405
rs870796	7	ELK1P1	G	A	0,017	0,003	3.274e-11	0,019	0,010	0,052
rs11782452	8	BNIP3L	G	A	0,015	0,003	1.258e-08	-0,007	0,009	0,458
rs12549853	8	PLEC	A	G	0,016	0,003	1.654e-09	-0,002	0,010	0,845
rs1431015	8	RNU2-54P	C	T	0,020	0,003	2.655e-14	0,007	0,009	0,468
rs1495741	8	NAT2	G	A	0,026	0,003	5.034e-18	0,030	0,011	0,007
rs1786342	8	SNX31	T	C	0,017	0,003	1.241e-11	-0,003	0,009	0,750
rs2737205	8	TRPS1	T	C	0,023	0,003	4.711e-20	0,007	0,010	0,445
rs2978062	8	ST3GAL1	G	T	0,019	0,003	3.225e-08	-0,014	0,013	0,290
rs445036	8	ZBTB10	T	C	0,019	0,003	5.794e-12	0,008	0,010	0,440
rs56352849	8	KCNB2	A	G	0,016	0,003	1.497e-08	-0,005	0,011	0,619
rs60862542	8	EIF3E	G	A	0,017	0,003	1.648e-08	-0,018	0,011	0,104
rs6473015	8	AC105242.1	C	A	0,019	0,003	2.994e-12	-0,030	0,010	0,003
rs716100	8	ZFAT	A	G	0,019	0,003	7.196e-13	0,014	0,010	0,131
rs76393968	8	MSR1	G	A	0,060	0,010	2.566e-09	0,034	0,034	0,319
rs9657541	8	CTD-2135J3.4	C	T	0,020	0,003	3.846e-10	0,008	0,013	0,513
rs1055710	9	FAM120AOS	G	A	0,018	0,003	7.149e-12	-0,019	0,010	0,059
rs10757291	9	CDKN2B-AS1	G	A	0,019	0,003	1.487e-14	-0,016	0,010	0,106
rs10811787	9	RP11-370B11.4	C	T	0,015	0,003	7.833e-09	-0,001	0,009	0,915
rs10869022	9	TRPM3	C	T	0,021	0,003	1.633e-11	0,000	0,011	0,965
rs10908903	9	GADD45G	T	G	0,015	0,003	1.288e-09	-0,025	0,009	0,008
rs11557154	9	DCAF12	T	C	0,024	0,004	3.100e-10	0,003	0,013	0,792
rs13301073	9	MAPKAP1	G	A	0,022	0,003	4.056e-17	-0,024	0,010	0,015
rs2378662	9	RP11-158D2.2	G	A	0,017	0,003	4.195e-11	-0,021	0,009	0,029
rs28831479	9	PTCH1	C	A	0,022	0,003	1.196e-14	0,009	0,011	0,425
rs7034716	9	TGFBR1	C	T	0,015	0,003	2.595e-08	0,001	0,011	0,950
rs7041137	9	RAD23B	T	C	0,017	0,003	7.298e-10	0,008	0,010	0,443
rs7872812	9	ASTN2	T	C	0,026	0,004	3.162e-13	0,008	0,014	0,538
rs10047326	10	PIP4K2A	A	C	0,017	0,003	1.263e-10	0,015	0,010	0,117
rs10509746	10	Y_RNA	C	T	0,027	0,003	1.943e-26	0,007	0,009	0,485
rs10821713	10	ANK3	T	C	0,017	0,003	5.068e-11	0,020	0,009	0,034
rs11012712	10		C	T	0,022	0,003	3.752e-12	-0,007	0,012	0,587
rs116454156	10	FFAR4	A	G	0,078	0,010	1.615e-14	-0,013	0,063	0,835
rs12244851	10	TCF7L2	T	C	0,015	0,003	1.450e-08	0,031	0,010	0,003
rs1832007	10	AKR1C4	G	A	0,057	0,003	6.779e-60	0,003	0,014	0,832
rs2274224	10	PLCE1	G	C	0,024	0,003	1.017e-20	-0,024	0,009	0,010

rs2801482	10	CAMK1D	G	A	0,050	0,008	1.130e-09	-0,053	0,026	0,045
rs293275	10	PRKG1	C	T	0,014	0,003	1.603e-08	0,006	0,009	0,530
rs3858325	10	GFRA1	T	C	0,019	0,003	1.713e-13	-0,009	0,009	0,334
rs4418728	10	CYP26A1	G	T	0,024	0,003	5.991e-21	0,038	0,010	0,000
rs4917962	10	NOLC1	T	G	0,024	0,004	5.216e-10	-0,005	0,013	0,710
rs7910087	10	C10orf11	T	C	0,017	0,003	6.229e-12	-0,006	0,009	0,530
rs7921105	10	BEND7	C	T	0,016	0,003	1.962e-10	0,012	0,009	0,201
rs9630085	10	FFAR4	G	A	0,022	0,003	9.144e-13	-0,003	0,011	0,780
rs1039481	11	PTPRJ	G	A	0,042	0,003	4.113e-48	-0,039	0,011	0,000
rs10767874	11	DCDC1	A	G	0,015	0,003	3.973e-08	-0,006	0,010	0,559
rs10892564	11	ARHGEF12	G	A	0,017	0,003	4.611e-11	0,012	0,010	0,208
rs10893499	11	ST3GAL4	G	A	0,022	0,004	3.842e-09	-0,035	0,013	0,006
rs11024614	11	HPS5	C	T	0,023	0,003	3.409e-18	0,010	0,009	0,276
rs11029620	11	NUP98	C	T	0,022	0,003	4.620e-13	-0,004	0,012	0,728
rs11031058	11	RPL12P30	T	C	0,022	0,003	5.389e-11	-0,007	0,013	0,614
rs117104648	11	AP5B1	C	T	0,036	0,005	3.240e-12	-0,011	0,023	0,652
rs117600498	11	ASCL2	C	T	0,038	0,007	9.548e-09	-0,023	0,028	0,404
rs12790261	11	KDM2A	A	C	0,031	0,005	1.211e-11	0,062	0,030	0,041
rs146345029	11	GIF	A	G	0,034	0,006	1.886e-08	-0,020	0,026	0,431
rs174554	11	FADS1	A	G	0,022	0,003	7.202e-17	0,022	0,010	0,027
rs2512525	11	USP35	T	C	0,024	0,003	1.427e-12	0,031	0,012	0,007
rs3213223	11	IGF2	A	G	0,076	0,003	9.35e-144	-0,015	0,015	0,296
rs34452566	11	RP11-587D21.4	T	G	0,018	0,003	1.032e-08	0,001	0,014	0,958
rs35023999	11	ANKK1	C	A	0,015	0,003	1.160e-09	-0,005	0,010	0,613
rs4936759	11	C11orf63	C	T	0,016	0,003	1.949e-10	0,002	0,010	0,840
rs4980661	11	AP000439.3	A	G	0,014	0,003	8.689e-09	0,017	0,010	0,078
rs61867536	11	MOB2	T	C	0,018	0,003	1.132e-12	0,004	0,010	0,681
rs61904289	11	EED	T	C	0,016	0,003	1.392e-09	0,017	0,010	0,100
rs625245	11	MRE11	G	T	0,016	0,003	4.364e-09	0,003	0,010	0,739
rs6485702	11	LRP4	T	C	0,017	0,003	1.415e-10	0,003	0,010	0,738
rs67257872	11	STK33	A	G	0,014	0,003	2.315e-08	-0,002	0,009	0,817
rs72858776	11	RP11-396O20.2	G	T	0,030	0,005	1.479e-10	0,012	0,016	0,443
rs7947951	11	ARNTL	G	A	0,020	0,003	1.025e-13	0,034	0,010	0,000
rs10745954	12	RP11-328J6.1	G	A	0,015	0,003	1.690e-09	0,020	0,009	0,030
rs10777540	12	CRADD	G	T	0,018	0,003	2.236e-12	-0,002	0,010	0,845
rs10841649	12	SLCO1B3	C	T	0,021	0,004	9.254e-09	0,031	0,015	0,033
rs10860237	12	RP11-1016B18.1	A	G	0,030	0,003	2.391e-28	-0,009	0,010	0,364
rs11064536	12	WNK1	T	C	0,020	0,003	2.941e-09	0,015	0,014	0,285
rs11111274	12	IGF1	G	A	0,080	0,003	7.59e-175	0,000	0,010	0,969
rs11175935	12	LRRK2	G	T	0,020	0,003	5.189e-10	-0,008	0,012	0,518
rs117564283	12	ACVRL1	T	C	0,029	0,005	5.046e-09	-0,005	0,022	0,835
rs12231073	12	RNA5SP358	T	G	0,017	0,003	5.421e-12	-0,009	0,010	0,334
rs12425869	12	SOCS2	A	G	0,018	0,003	3.957e-09	0,005	0,011	0,673
rs1351394	12	HMGA2	C	T	0,024	0,003	6.924e-21	0,020	0,010	0,034
rs1800574	12	HNF1A	T	C	0,145	0,007	3.641e-84	0,026	0,028	0,359

rs2230281	12	GALNT4	A	G	0,016	0,003	4.010e-09	-0,020	0,011	0,061
rs2460488	12	RP11-110L15.1	G	A	0,026	0,003	1.096e-14	0,004	0,014	0,762
rs247917	12	ARID2	T	C	0,015	0,003	1.322e-09	0,016	0,009	0,092
rs2657879	12	GLS2	A	G	0,020	0,003	9.621e-10	0,029	0,012	0,018
rs2856321	12	ETV6	G	A	0,026	0,003	1.583e-23	0,009	0,010	0,333
rs3759302	12	KIAA1551	A	T	0,021	0,003	6.949e-11	0,005	0,012	0,636
rs4547160	12	AVPR1A	T	G	0,018	0,003	3.168e-11	-0,014	0,010	0,173
rs7314285	12	CUX2	G	T	0,052	0,005	2.043e-25	-0,016	0,018	0,372
rs75938105	12	RP11-110L15.2	T	C	0,047	0,007	7.468e-12	-0,003	0,030	0,916
rs773116	12	ERBB3	G	A	0,016	0,003	1.265e-10	0,013	0,010	0,186
rs78607331	12	R3HDM2	C	T	0,037	0,006	6.059e-10	0,015	0,028	0,590
rs9738365	12	RP11-428G5.4	A	C	0,058	0,003	1.905e-92	-0,010	0,011	0,362
rs1170158	13	DGKH	T	G	0,021	0,003	1.041e-10	0,002	0,011	0,888
rs118081390	13	FNDC3A	G	A	0,028	0,005	1.353e-08	-0,018	0,020	0,351
rs1535793	13	LRCH1	A	G	0,024	0,003	7.422e-17	0,020	0,011	0,063
rs6602909	13	GAS6	C	T	0,020	0,003	4.354e-14	0,015	0,012	0,205
rs71432868	13	SNORA25	C	T	0,028	0,005	3.105e-08	-0,023	0,035	0,508
rs7323205	13	LINC00676	C	T	0,015	0,003	9.354e-09	0,011	0,010	0,233
rs9532512	13	LINC00598	A	G	0,043	0,003	8.024e-40	-0,011	0,012	0,371
rs9573360	13	LINC00402	A	C	0,014	0,003	1.597e-08	0,001	0,009	0,907
rs9583151	13	AL354741.1	C	T	0,014	0,003	2.708e-08	-0,012	0,010	0,216
rs10136874	14	DLK1	G	T	0,023	0,003	2.350e-20	-0,009	0,010	0,360
rs10145154	14	NRXN3	T	C	0,018	0,003	9.924e-10	0,021	0,012	0,083
rs1061638	14	AHSA1	G	A	0,018	0,003	7.484e-11	0,022	0,010	0,027
rs1115897	14	UNC79	A	C	0,021	0,003	1.540e-14	0,021	0,010	0,033
rs13379043	14	ELMSAN1	T	C	0,025	0,003	5.776e-18	0,009	0,011	0,386
rs168961	14	ZFP36L1	G	A	0,018	0,003	2.889e-13	0,010	0,009	0,311
rs17106640	14	ACTN1	G	A	0,017	0,003	8.431e-11	0,009	0,010	0,377
rs175043	14	EIF2B2	G	A	0,018	0,003	1.834e-12	0,040	0,009	0,000
rs28396553	14	AL162511.1	T	C	0,015	0,003	2.962e-09	0,013	0,010	0,198
rs28929474	14	SERPINA1	C	T	0,063	0,009	1.294e-12	0,147	0,045	0,001
rs33912345	14	SIX6	C	A	0,023	0,003	2.363e-19	-0,014	0,010	0,133
rs36215895	14	SYNE2	C	T	0,082	0,013	2.121e-10	0,115	0,071	0,102
rs78598185	14	SLC24A4	G	A	0,029	0,004	7.090e-11	-0,015	0,018	0,397
rs79936318	14	SYNE2	A	G	0,017	0,003	3.875e-08	-0,022	0,016	0,158
rs8017377	14	NYNRIN	G	A	0,017	0,003	3.353e-11	-0,008	0,010	0,460
rs11856160	15	CHD2	A	G	0,021	0,003	2.843e-09	-0,006	0,014	0,647
rs12442867	15	RP11-299H22.1	A	C	0,017	0,003	9.329e-11	-0,005	0,009	0,571
rs12593755	15	RP11-97O12.3	T	G	0,016	0,003	1.846e-09	-0,001	0,009	0,940
rs12912439	15	LINC01197	T	C	0,022	0,003	1.246e-15	0,016	0,011	0,140
rs142354201	15	PGPEP1L	G	A	0,034	0,006	3.677e-09	-0,004	0,031	0,900
rs17747633	15	KNL1	G	A	0,015	0,003	3.080e-09	-0,001	0,010	0,912
rs2004839	15	RP11-299H22.3	G	A	0,021	0,003	4.336e-10	-0,010	0,013	0,450
rs2311313	15	RP11-35O15.1	G	T	0,019	0,003	1.691e-08	0,021	0,013	0,096
rs2930313	15	CCDC33	G	A	0,028	0,005	4.141e-09	0,016	0,015	0,289

rs4545755	15	CYP19A1	G	A	0,016	0,003	3.865e-10	-0,008	0,010	0,376
rs55707100	15	MAP1A	C	T	0,151	0,008	1.406e-76	0,001	0,031	0,973
rs5742915	15	PML	C	T	0,025	0,003	1.723e-22	-0,006	0,010	0,540
rs79076440	15	USP3	A	G	0,019	0,003	1.164e-08	0,001	0,012	0,918
rs8033075	15	PIAS1	A	G	0,045	0,005	1.061e-17	-0,022	0,023	0,344
rs11077337	16	ZNF597	T	G	0,015	0,003	1.454e-09	0,002	0,009	0,854
rs11149612	16	RP11-505K9.4	C	T	0,027	0,003	1.131e-26	-0,014	0,011	0,189
rs111792934	16	HAS3	C	T	0,022	0,003	1.430e-10	0,011	0,013	0,370
rs116971887	16	SALL1	G	T	0,036	0,006	4.895e-09	-0,006	0,030	0,849
rs12597502	16	CHD9	G	A	0,015	0,003	4.528e-08	-0,001	0,010	0,918
rs12927172	16	IL4R	A	G	0,015	0,003	3.066e-09	0,013	0,009	0,180
rs12935091	16	ZNF19	G	A	0,035	0,006	2.235e-09	-0,022	0,028	0,430
rs12935465	16	XYLT1	T	C	0,016	0,003	5.769e-11	-0,002	0,010	0,795
rs147491123	16	LINC01572	C	T	0,036	0,007	3.286e-08	0,034	0,031	0,270
rs1532824	16	ATF7IP2	A	C	0,017	0,003	4.521e-09	-0,007	0,011	0,530
rs1548917	16	RP11-461O7.1	T	C	0,015	0,003	2.361e-09	0,000	0,009	0,989
rs1657125	16	MEIOB	T	G	0,032	0,004	2.007e-18	-0,008	0,013	0,534
rs17299478	16	NOB1	C	T	0,032	0,003	2.497e-21	-0,008	0,013	0,570
rs2023762	16	SYT17	T	C	0,015	0,003	5.991e-09	0,008	0,010	0,400
rs4786350	16	IFT140	C	G	0,036	0,006	6.048e-09	-0,001	0,025	0,956
rs4788220	16	FAM57B	G	A	0,017	0,003	5.263e-12	-0,003	0,010	0,761
rs4985062	16	USP7	T	C	0,015	0,003	2.040e-09	0,007	0,010	0,472
rs61731445	16		C	T	0,028	0,005	1.958e-08	-0,003	0,023	0,902
rs7204824	16	LMF1	C	T	0,024	0,003	1.168e-16	-0,016	0,010	0,132
rs72761177	16	NUBP2	A	G	0,077	0,004	7.205e-70	0,009	0,016	0,549
rs74774288	16	RP11-420N3.3	G	T	0,027	0,003	2.955e-17	-0,009	0,013	0,477
rs7498665	16	SH2B1	G	A	0,019	0,003	7.173e-14	0,019	0,010	0,060
rs750952	16	ZNF646	C	T	0,032	0,003	1.105e-34	-0,002	0,010	0,851
rs753108	16	CMIP	A	G	0,025	0,003	1.606e-17	0,010	0,011	0,398
rs80253441	16	IGFALS	T	C	0,131	0,011	8.227e-36	0,047	0,040	0,240
rs8054054	16	CCDC154	G	A	0,015	0,003	1.026e-09	-0,003	0,011	0,810
rs8054322	16	GSE1	A	G	0,015	0,003	4.723e-09	0,010	0,010	0,331
rs8059803	16	CMIP	A	G	0,031	0,003	4.415e-29	0,012	0,011	0,257
rs8182173	16	CORO7-PAM16	T	C	0,018	0,003	1.610e-09	0,016	0,011	0,142
rs142377191	17	DCAF7	A	G	0,125	0,009	2.048e-46	0,021	0,050	0,671
rs1801689	17	APOH	C	A	0,089	0,007	1.248e-33	0,043	0,036	0,238
rs199525	17	WNT3	G	T	0,020	0,003	5.613e-11	0,039	0,013	0,003
rs2309401	17	NLRP1	T	G	0,015	0,003	6.785e-09	-0,002	0,010	0,845
rs35819807	17	KCNH6	T	C	0,017	0,003	2.387e-09	-0,002	0,012	0,866
rs3760237	17	SCN4A	C	T	0,024	0,003	1.441e-21	0,003	0,009	0,782
rs4075483	17	BAIAP2	C	T	0,017	0,003	1.312e-10	0,010	0,011	0,355
rs4789227	17	UNK	T	C	0,015	0,003	4.335e-09	0,037	0,010	0,000
rs56030650	17	GSDMA	C	A	0,022	0,003	2.417e-18	-0,009	0,009	0,321
rs6416868	17	TTC19	A	G	0,019	0,003	3.115e-14	-0,005	0,009	0,604
rs6501601	17	POLR3KP2	G	A	0,015	0,003	4.071e-09	-0,014	0,011	0,204

rs668799	17	COASY	C	T	0,018	0,003	4.019e-10	-0,037	0,010	0,000
rs7502910	17	WDR81	A	G	0,016	0,003	1.528e-10	-0,002	0,010	0,804
rs76708468	17	1ERN	C	T	0,087	0,006	3.084e-41	0,029	0,041	0,478
rs77542162	17	ABCA6	G	A	0,054	0,009	2.412e-10	0,016	0,044	0,719
rs8075153	17	RAI1	C	T	0,021	0,003	1.831e-17	0,036	0,010	0,000
rs8079923	17	AKAP10	C	T	0,016	0,003	3.664e-08	-0,010	0,011	0,375
rs9892862	17	Y_RNA	G	A	0,022	0,003	9.705e-14	-0,002	0,011	0,849
rs11152071	18	RP11-1151B14.2	C	T	0,020	0,003	8.957e-12	-0,001	0,011	0,929
rs12454712	18	BCL2	T	C	0,018	0,003	4.051e-12	0,030	0,011	0,005
rs190102446	18	RP11-27G24.1	C	T	0,041	0,007	2.452e-09	0,037	0,027	0,175
rs57551555	18	RP11-176N18.2	T	G	0,019	0,003	5.817e-13	-0,012	0,010	0,266
rs585187	18	MRPS5P4	T	G	0,015	0,003	3.712e-09	-0,014	0,009	0,129
rs8084351	18	DCC	G	A	0,015	0,003	8.537e-10	-0,014	0,010	0,137
rs8095538	18	-	G	T	0,020	0,003	8.447e-14	0,002	0,010	0,865
rs8097893	18	GALR1	A	G	0,058	0,006	1.723e-20	0,006	0,022	0,772
rs11671304	19	ZC3H4	C	T	0,018	0,003	6.884e-11	-0,024	0,011	0,027
rs12975366	19	LILRB5	T	C	0,020	0,003	2.253e-15	0,014	0,011	0,233
rs2287922	19	RASIP1	G	A	0,030	0,003	1.833e-33	-0,003	0,010	0,785
rs296361	19	SULT2A1	A	G	0,025	0,003	3.421e-13	-0,014	0,013	0,285
rs34536443	19	TYK2	G	C	0,045	0,006	9.964e-14	0,066	0,036	0,068
rs3760954	19	MIR7-3HG	T	C	0,024	0,004	2.909e-10	0,032	0,013	0,016
rs58560372	19	SPINT2	C	T	0,020	0,003	1.331e-08	0,002	0,013	0,885
rs58658292	19	ZNF536	G	A	0,030	0,006	4.618e-08	0,006	0,022	0,793
rs62102136	19	LSM14A	C	T	0,016	0,003	6.527e-09	-0,012	0,011	0,296
rs6510033	19	AC005597.1	A	G	0,020	0,003	1.147e-12	-0,008	0,011	0,461
rs6510177	19	ZNF536	T	C	0,023	0,003	1.598e-12	0,020	0,014	0,157
rs6510832	19	KDM4B	G	T	0,033	0,005	2.638e-10	-0,013	0,023	0,564
rs67868323	19	ZBTB7A	T	G	0,016	0,003	2.205e-08	0,001	0,012	0,934
rs7254601	19	COX6B1	G	A	0,016	0,003	2.551e-08	0,019	0,011	0,099
rs7256521	19	ZNF845	G	A	0,015	0,003	9.761e-10	-0,002	0,010	0,859
rs8105174	19	DNMT1	C	T	0,050	0,003	7.971e-54	0,020	0,014	0,139
rs8112883	19	INSR	G	T	0,017	0,003	1.166e-09	0,004	0,010	0,722
rs8113618	19	QTRT1	T	C	0,031	0,003	1.185e-33	0,030	0,010	0,002
rs16995311	20	PTPN1	A	C	0,040	0,005	5.559e-18	0,019	0,020	0,349
rs17265513	20	ZHX3	T	C	0,022	0,003	3.454e-12	0,024	0,014	0,084
rs2207132	20	LINC01728	G	A	0,048	0,007	1.064e-11	-0,136	0,035	0,000
rs2424396	20	LINC01726	G	A	0,033	0,004	2.245e-14	-0,001	0,015	0,964
rs2738787	20	TNFRSF6B	A	G	0,037	0,005	1.251e-15	0,000	0,019	0,989
rs4809401	20	NPBWR2	T	C	0,023	0,004	6.488e-10	-0,016	0,016	0,326
rs6037508	20	SLC4A11	G	T	0,017	0,003	8.740e-09	-0,005	0,012	0,676
rs6046825	20	RALGAPA2	A	C	0,024	0,003	2.108e-17	0,011	0,010	0,279
rs6088579	20	NCOA6	G	A	0,027	0,003	8.509e-17	0,025	0,012	0,038
rs7267595	20	JAG1	A	C	0,015	0,003	9.437e-10	-0,003	0,009	0,710
rs7508949	20	CRNKL1	C	G	0,025	0,003	1.490e-22	0,005	0,009	0,615
rs75989562	20	APMAP	A	G	0,030	0,005	8.093e-09	-0,019	0,028	0,491

rs9978775	21	BRWD1-AS1	G	A	0,019	0,003	2.351e-13	0,015	0,009	0,112
rs12106594	22	EIF4ENIF1	T	C	0,036	0,006	1.588e-09	0,026	0,020	0,202
rs2412973	22	HORMAD2	A	C	0,014	0,003	3.334e-08	0,004	0,009	0,659
rs4823324	22	ATXN10	T	C	0,016	0,003	1.938e-10	-0,003	0,009	0,725
rs5755948	22	RBFOX2	A	G	0,028	0,004	4.375e-14	0,010	0,014	0,473
rs6519133	22	JOSD1	T	C	0,029	0,003	7.731e-30	0,026	0,010	0,009
rs8138950	22	ZNRF3	C	T	0,015	0,003	1.323e-09	0,013	0,009	0,150
rs9611565	22	TEF	T	C	0,029	0,003	3.825e-23	0,002	0,011	0,878

Chr, chromosome; EA, effect allele; OA, other allele; SE, standard error.

ESM Table 3. Summary statistics of the single-nucleotide polymorphisms associated with insulin-like growth factor-1 and their associations with heart failure

					IGF-1			Heart failure		
**SNP**	**Chr**	**Nearby gene**	**EA**	**OA**	**Beta**	**SE**	***p***	**Beta**	**SE**	***p***
rs10159299	1	PLXNA2	C	T	0,018	0,003	5.112e-12	0,003	0,008	0,672
rs1046011	1	LEPROT	T	C	0,021	0,003	3.303e-14	0,000	0,009	0,971
rs10779509	1	RP1-272L16.1	C	T	0,014	0,003	2.109e-08	-0,002	0,008	0,819
rs11165778	1	HFM1	A	G	0,016	0,003	1.492e-08	0,016	0,009	0,071
rs112436634	1	PEX14	T	C	0,016	0,003	1.749e-09	0,010	0,008	0,213
rs1127313	1	ADAR	G	A	0,024	0,003	1.394e-21	0,019	0,008	0,015
rs11577063	1	AXDND1	T	G	0,020	0,003	1.511e-11	0,001	0,010	0,920
rs12141189	1	HLX	T	C	0,045	0,003	4.135e-53	0,022	0,009	0,015
rs1223763	1	RP11-53A1.3	T	G	0,024	0,003	8.461e-13	-0,010	0,010	0,337
rs12723255	1	EIF4G3	C	T	0,017	0,003	3.229e-11	-0,002	0,008	0,820
rs12749024	1	PAPPA2	T	C	0,075	0,004	7.72e-100	-0,004	0,012	0,732
rs140604451	1	GSTM2	G	A	0,054	0,008	3.524e-11	-0,027	0,023	0,237
rs1430753	1	WLS	A	G	0,021	0,003	7.085e-11	0,004	0,010	0,662
rs143885630	1	SMG7	G	A	0,030	0,004	1.358e-15	0,025	0,012	0,036
rs165316	1	RPL5P6	G	A	0,073	0,003	9.66e-119	-0,010	0,010	0,306
rs17037452	1	CLCN6	A	G	0,023	0,003	1.658e-11	-0,017	0,011	0,113
rs17393144	1	MIR34AHG	A	G	0,016	0,003	9.319e-09	0,014	0,009	0,096
rs1825813	1	C1orf146	A	G	0,023	0,003	1.879e-13	0,013	0,010	0,192
rs2075995	1	E2F2	C	A	0,014	0,003	9.790e-09	-0,011	0,008	0,183
rs2724373	1	C1orf132	C	T	0,019	0,003	1.436e-12	-0,006	0,008	0,470
rs2802330	1	PDIK1L	G	A	0,031	0,003	4.632e-21	-0,011	0,011	0,317
rs2802951	1	RN7SL668P	A	G	0,016	0,003	4.970e-09	-0,006	0,009	0,508
rs2819336	1	PTPRF	T	C	0,027	0,003	1.550e-25	-0,016	0,008	0,050
rs3131646	1	MYCL	G	T	0,016	0,003	1.710e-08	-0,006	0,009	0,463
rs36086195	1	ARHGEF19-AS1	C	T	0,019	0,003	1.359e-13	0,007	0,008	0,367
rs4306136	1	RP11-103C3.1	A	G	0,017	0,003	1.997e-11	-0,006	0,008	0,496
rs569356	1	OPRD1	G	A	0,027	0,004	5.953e-14	0,006	0,012	0,616
rs599839	1	CELSR2	G	A	0,031	0,003	9.629e-26	-0,057	0,010	0,000
rs61780439	1	SLFNL1	G	A	0,021	0,003	5.323e-12	-0,012	0,009	0,188
rs6659176	1	NR0B2	C	G	0,042	0,005	1.354e-19	-0,017	0,014	0,238
rs6701954	1	USP48	T	G	0,014	0,003	3.497e-08	-0,001	0,009	0,902
rs684818	1	RP4-781K5.7	T	C	0,024	0,003	9.658e-21	0,002	0,008	0,823
rs708108	1	WNT3A	T	C	0,015	0,003	5.872e-09	0,001	0,008	0,929
rs7517340	1	AKT3	C	T	0,035	0,003	2.123e-26	0,000	0,010	0,994
rs7528548	1	PYGO2	C	T	0,054	0,009	7.954e-10	-0,006	0,025	0,824
rs7539178	1	JAK1	C	A	0,026	0,004	1.365e-12	0,008	0,011	0,477
rs7545345	1	NUCKS1	C	T	0,026	0,004	1.032e-12	-0,002	0,012	0,858
rs75681856	1	RABGAP1L	T	C	0,023	0,004	5.171e-09	0,001	0,013	0,950
rs75907879	1	SPEN	T	C	0,024	0,004	1.425e-10	0,008	0,016	0,610
rs77369503	1	RGS4	G	A	0,045	0,007	1.397e-10	-0,029	0,025	0,243

rs903908	1	SKI	C	T	0,016	0,003	2.176e-10	-0,009	0,008	0,238
rs11677980	2	LBH	G	A	0,015	0,003	3.043e-08	0,008	0,009	0,387
rs11678946	2	EPHA4	A	C	0,014	0,003	3.143e-08	-0,001	0,008	0,867
rs12471768	2	SERTAD2	C	T	0,022	0,003	1.639e-15	-0,003	0,009	0,722
rs1260326	2	GCKR	C	T	0,063	0,003	9.66e-133	-0,009	0,008	0,280
rs12710648	2	SMC6	A	G	0,017	0,003	5.703e-12	0,010	0,008	0,197
rs1465529	2	SP110	T	C	0,019	0,003	1.386e-12	-0,007	0,008	0,420
rs17050272	2	AC073257.2	G	A	0,024	0,003	3.201e-20	0,021	0,008	0,010
rs17323117	2	NEU2	G	A	0,029	0,005	7.980e-10	-0,010	0,014	0,480
rs17400325	2	PDE11A	C	T	0,054	0,006	9.393e-17	-0,002	0,022	0,937
rs2674492	2	CYBRD1	A	G	0,014	0,003	4.835e-08	-0,001	0,008	0,898
rs35135518	2	AC010145.4	T	C	0,029	0,004	1.521e-12	0,007	0,013	0,613
rs35641591	2	PCBP1-AS1	C	T	0,050	0,006	1.575e-14	-0,007	0,021	0,736
rs3791679	2	EFEMP1	G	A	0,018	0,003	4.338e-09	0,012	0,010	0,206
rs4402747	2	NEU2	A	G	0,016	0,003	2.960e-10	-0,003	0,008	0,670
rs58387407	2	CACNB4	G	A	0,018	0,003	3.502e-08	-0,020	0,010	0,039
rs62136965	2	RNU6-566P	C	T	0,037	0,006	6.429e-10	0,006	0,019	0,762
rs62182127	2	VIL1	A	G	0,019	0,003	2.712e-14	-0,006	0,009	0,548
rs6435156	2	BMPR2	C	T	0,024	0,003	2.045e-17	-0,005	0,009	0,548
rs6437249	2	HDLBP	C	T	0,019	0,003	4.559e-12	0,012	0,009	0,178
rs6544549	2	KCNG3	T	C	0,024	0,004	1.592e-10	0,029	0,012	0,014
rs6749680	2	ALMS1	A	G	0,015	0,003	9.868e-09	-0,015	0,008	0,055
rs6760135	2	ASXL2	T	C	0,050	0,003	1.194e-63	-0,010	0,010	0,289
rs702878	2	AC074391.1	A	G	0,014	0,003	1.815e-08	0,003	0,008	0,723
rs73954943	2	BCL2L11	A	G	0,031	0,005	1.063e-09	0,022	0,017	0,202
rs7574340	2	SLC8A1	T	C	0,017	0,003	2.971e-10	0,007	0,009	0,443
rs7578633	2	PAX8	T	C	0,018	0,003	3.379e-12	-0,002	0,008	0,829
rs112893170	3	FEZF2	T	C	0,020	0,003	1.527e-10	0,004	0,010	0,678
rs11717397	3	UBE2E2	G	A	0,015	0,003	2.572e-09	0,008	0,008	0,332
rs11928797	3	UBP1	A	C	0,030	0,004	2.428e-14	0,003	0,013	0,789
rs12491473	3	CCDC12	G	A	0,020	0,003	5.913e-15	-0,003	0,008	0,726
rs13069961	3	KALRN	G	A	0,018	0,003	3.680e-09	0,012	0,010	0,208
rs13073970	3	EIF5A2	T	G	0,025	0,003	2.016e-15	-0,006	0,010	0,560
rs1822825	3	PPARG	G	A	0,014	0,003	2.886e-08	0,006	0,008	0,418
rs2268829	3	DGKG	G	A	0,018	0,003	3.038e-09	-0,001	0,010	0,889
rs2607748	3	CHCHD4	C	T	0,017	0,003	3.289e-11	0,007	0,008	0,379
rs3772102	3	ST3GAL6	G	T	0,020	0,003	5.954e-16	0,008	0,008	0,321
rs4678497	3	SUSD5	C	T	0,017	0,003	1.959e-10	0,002	0,008	0,843
rs504603	3	BZW1P1	C	T	0,029	0,005	2.402e-09	0,012	0,016	0,459
rs55717031	3	MRPS22	G	T	0,032	0,003	2.474e-31	0,016	0,009	0,060
rs56062334	3	LINC02068	T	C	0,017	0,003	1.839e-11	0,003	0,008	0,699
rs62263345	3	BBX	A	G	0,028	0,004	1.640e-14	-0,007	0,012	0,557
rs62280667	3	SENP7	C	T	0,028	0,003	2.477e-26	-0,011	0,008	0,199
rs6440008	3	ZBTB38	T	C	0,035	0,003	1.113e-41	-0,007	0,008	0,360
rs66707192	3	HRG	G	A	0,018	0,003	4.415e-10	-0,016	0,009	0,078

rs687339	3	KRT18P35	T	C	0,040	0,003	2.666e-40	0,023	0,010	0,015
rs73238159	3	XRN1	C	T	0,025	0,004	1.059e-11	-0,008	0,012	0,487
rs7625680	3	ATG7	A	G	0,015	0,003	7.693e-09	0,011	0,008	0,194
rs7628689	3	C3orf38	G	A	0,029	0,003	6.825e-17	0,008	0,011	0,473
rs811332	3	MRAS	C	T	0,019	0,003	2.011e-09	-0,020	0,010	0,049
rs9819762	3	PIK3CA	T	C	0,019	0,003	1.212e-08	-0,018	0,010	0,089
rs1055582	4	UBE2K	C	T	0,027	0,003	1.578e-27	0,013	0,008	0,112
rs111443396	4	LINC01091	C	T	0,026	0,004	2.015e-10	0,015	0,012	0,239
rs1229984	4	ADH1B	T	C	0,104	0,009	1.219e-34	-0,039	0,028	0,162
rs13108218	4	HGFAC	G	A	0,017	0,003	5.920e-11	-0,016	0,008	0,059
rs17429745	4	RP11-556I14.1	G	T	0,026	0,003	2.045e-21	-0,002	0,008	0,818
rs1902023	4	UGT2B15	A	C	0,025	0,003	1.951e-23	0,027	0,009	0,002
rs2280099	4	TIGD2	G	A	0,025	0,003	1.923e-14	-0,015	0,011	0,156
rs35036084	4	RP11-145G20.1	T	C	0,017	0,003	8.218e-11	0,003	0,008	0,691
rs3804173	4	PRDM5	G	A	0,020	0,003	4.484e-13	0,009	0,008	0,311
rs4394044	4	SORBS2	T	C	0,014	0,003	4.708e-08	0,003	0,008	0,740
rs62302688	4	GABRA2	G	A	0,039	0,004	4.057e-20	0,008	0,015	0,579
rs62334147	4	DDX60L	C	T	0,019	0,003	4.261e-09	0,008	0,010	0,447
rs62342064	4	RP11-119H12.3	C	T	0,022	0,004	1.836e-09	0,001	0,013	0,950
rs6532798	4	ADH4	T	C	0,037	0,003	1.364e-41	-0,009	0,009	0,305
rs6827641	4	HHIP	T	C	0,014	0,003	2.623e-08	0,001	0,008	0,865
rs6853741	4	ARHGAP10	A	G	0,024	0,003	1.676e-16	-0,005	0,009	0,603
rs7667562	4	LARP1B	C	A	0,016	0,003	3.770e-09	0,010	0,009	0,234
rs976002	4	TMPRSS11E	A	G	0,036	0,003	4.672e-35	-0,012	0,010	0,216
rs11242236	5	C5orf66	G	A	0,025	0,003	1.957e-22	-0,004	0,008	0,585
rs11954036	5	PDE4D	T	C	0,037	0,003	1.936e-44	0,005	0,008	0,563
rs12108803	5	TBCA	G	T	0,033	0,006	1.015e-08	-0,005	0,018	0,799
rs12520263	5	RP11-357F12.1	T	G	0,017	0,003	2.579e-09	-0,005	0,009	0,572
rs13168379	5	CPEB4	A	G	0,031	0,005	6.148e-10	0,037	0,015	0,012
rs13178887	5	MEF2C-AS1	T	C	0,023	0,003	2.712e-19	0,012	0,008	0,137
rs1498603	5	PDE4D	T	G	0,031	0,005	1.170e-09	-0,010	0,016	0,554
rs17714046	5	TRIM41	C	T	0,042	0,006	1.197e-12	0,018	0,018	0,337
rs2042253	5	MIR5197	T	C	0,023	0,003	1.017e-14	-0,009	0,009	0,302
rs2227819	5	F2R	T	C	0,022	0,004	4.322e-08	0,025	0,013	0,056
rs2366398	5	CTD-2151A2.3	T	G	0,018	0,003	1.613e-09	-0,005	0,010	0,629
rs258775	5	ARHGAP26	A	C	0,025	0,003	3.394e-14	0,015	0,010	0,149
rs26822	5	PPIP5K2	G	A	0,017	0,003	9.845e-11	-0,010	0,009	0,230
rs28650790	5	C5orf67	T	C	0,018	0,003	4.108e-08	0,028	0,010	0,006
rs329122	5	JADE2	A	G	0,018	0,003	1.312e-12	0,009	0,008	0,268
rs35668185	5	SLIT3	T	C	0,056	0,003	3.402e-73	0,001	0,010	0,953
rs3734166	5	CDC25C	A	G	0,028	0,003	8.063e-22	0,017	0,009	0,053
rs6180	5	GHR	A	C	0,035	0,003	6.019e-43	-0,006	0,008	0,488
rs6895953	5	RP11-357F12.1	G	A	0,024	0,003	5.167e-21	0,000	0,008	0,994
rs72758321	5	PLCXD3	G	A	0,047	0,006	3.926e-15	0,001	0,021	0,979
rs73271090	5	CTB-1I21.1	G	A	0,044	0,003	7.844e-39	-0,014	0,011	0,186

rs7719168	5	15ARL	C	A	0,030	0,004	2.429e-14	0,018	0,013	0,167
rs80170948	5	SREK1IP1	G	T	0,039	0,006	2.221e-09	-0,016	0,022	0,450
rs840809	5	CTD-2232E5.2	A	C	0,016	0,003	1.183e-08	0,001	0,009	0,890
rs9292578	5	PRLR	C	A	0,040	0,006	3.195e-10	-0,023	0,021	0,253
rs1042335	6	HLA-DPB1	T	C	0,015	0,003	3.568e-08	-0,011	0,009	0,209
rs1130838	6	HLA-C	C	T	0,026	0,003	1.848e-23	-0,013	0,009	0,178
rs113127944	6	CENPW	A	G	0,051	0,008	6.115e-11	-0,022	0,025	0,374
rs1150781	6	C6orf1	G	C	0,026	0,004	2.337e-09	-0,035	0,015	0,016
rs1165196	6	SLC17A1	G	A	0,029	0,003	6.414e-30	-0,001	0,008	0,888
rs12110787	6	MAP3K4	A	C	0,022	0,004	1.857e-08	0,013	0,013	0,291
rs12194618	6	ZFAND3	A	G	0,017	0,003	2.686e-11	0,004	0,008	0,620
rs2296198	6	RNF144B	C	T	0,016	0,003	1.937e-08	0,001	0,009	0,884
rs2397112	6	RP11-228O6.2	A	G	0,019	0,003	3.313e-13	-0,018	0,008	0,026
rs28362677	6	BTNL2	T	C	0,032	0,004	1.763e-19	0,009	0,011	0,446
rs3008051	6	PDE10A	C	T	0,014	0,003	1.946e-08	0,001	0,008	0,928
rs3127579	6	SLC22A2	A	G	0,033	0,004	1.230e-19	-0,002	0,012	0,838
rs3890746	6	L3MBTL3	C	T	0,020	0,003	1.060e-15	0,016	0,008	0,042
rs41285260	6	CENPW	T	G	0,039	0,004	5.123e-18	-0,019	0,015	0,198
rs4709995	6	SDIM1	T	C	0,042	0,003	7.369e-60	0,005	0,008	0,569
rs584955	6	TMEM14C	A	G	0,036	0,006	2.579e-09	-0,029	0,020	0,153
rs670049	6	Y_RNA	A	C	0,019	0,003	6.417e-13	-0,002	0,008	0,859
rs6916994	6	GJB7	C	T	0,029	0,003	1.519e-31	0,027	0,008	0,001
rs6924225	6	RUNX2	G	A	0,019	0,003	1.442e-08	0,001	0,011	0,935
rs73382439	6	E2F3	C	T	0,019	0,003	1.800e-08	-0,002	0,012	0,868
rs7740433	6	CNPY3	A	G	0,017	0,003	1.504e-08	0,002	0,009	0,815
rs7758644	6	RP1-155D22.1	A	C	0,019	0,003	1.514e-08	-0,003	0,010	0,754
rs7774230	6	ESR1	T	C	0,026	0,003	5.464e-25	-0,001	0,008	0,902
rs790513	6	OPRM1	C	A	0,025	0,003	1.575e-18	0,000	0,009	0,981
rs9321106	6	PTPRK	A	G	0,018	0,003	2.408e-08	-0,011	0,010	0,297
rs9322822	6	LIN28B-AS1	C	T	0,015	0,003	1.349e-08	0,016	0,008	0,052
rs9364815	6	PDE10A	A	G	0,015	0,003	2.829e-08	-0,001	0,008	0,954
rs9398171	6	FOXO3	T	C	0,050	0,003	9.510e-74	0,005	0,009	0,584
rs9398891	6	LAMA2	T	C	0,017	0,003	1.108e-10	-0,027	0,009	0,002
rs998584	6	VEGFA	A	C	0,020	0,003	1.211e-15	0,003	0,008	0,674
rs10246481	7	AC073133.2	G	A	0,015	0,003	1.499e-08	-0,001	0,008	0,918
rs10252510	7	GHRHR	G	A	0,020	0,003	8.179e-14	-0,001	0,009	0,896
rs1050327	7	ZMIZ2	A	G	0,017	0,003	2.907e-11	0,012	0,008	0,139
rs114949263	7	TMEM176B	T	C	0,027	0,004	1.283e-11	-0,008	0,013	0,522
rs11556924	7	ZC3HC1	C	T	0,016	0,003	9.798e-10	0,022	0,008	0,007
rs1182174	7	GNA12	A	G	0,021	0,003	4.808e-14	-0,012	0,009	0,163
rs12666306	7	RP11-222O23.1	G	A	0,017	0,003	1.702e-11	0,010	0,008	0,223
rs12699547	7	MAD1L1	C	T	0,021	0,003	2.438e-16	-0,001	0,008	0,951
rs145188037	7	IGFBP3	A	G	0,120	0,011	1.749e-27	0,052	0,032	0,102
rs17145738	7	TBL2	T	C	0,034	0,004	8.750e-19	-0,010	0,012	0,406
rs1986692	7	EXOC4	G	A	0,015	0,003	8.640e-09	0,001	0,008	0,866

rs2048672	7	LINC-PINT	C	A	0,018	0,003	6.405e-11	0,000	0,008	0,961
rs207212	7	LINC00513	C	T	0,028	0,004	1.659e-11	-0,001	0,015	0,922
rs2228078	7	GHRHR	C	T	0,057	0,010	4.076e-08	0,037	0,037	0,310
rs2250243	7	ZNF316	C	T	0,024	0,003	1.694e-15	-0,004	0,009	0,630
rs2270628	7	IGFBP3	T	C	0,033	0,003	3.737e-25	-0,016	0,010	0,105
rs273956	7	CREB3L2	A	G	0,021	0,003	5.369e-16	-0,004	0,008	0,641
rs2896395	7	SND1	C	T	0,015	0,003	2.446e-08	0,013	0,009	0,133
rs34312198	7	ZNF3	A	C	0,024	0,004	1.345e-09	0,004	0,013	0,759
rs35862187	7	AUTS2	A	G	0,031	0,006	3.484e-08	-0,026	0,017	0,128
rs411717	7	COL1A2	T	C	0,015	0,003	2.144e-09	0,001	0,008	0,953
rs4719393	7	DGKB	T	G	0,027	0,003	1.290e-22	0,006	0,009	0,495
rs74657816	7	HMGN1P19	T	G	0,047	0,005	3.006e-18	-0,007	0,018	0,688
rs7783012	7	FOXP2	G	A	0,016	0,003	1.561e-10	0,001	0,008	0,916
rs7802508	7	ZFAND2A	A	G	0,021	0,003	9.477e-17	-0,006	0,008	0,479
rs79881512	7	AC073325.1	C	T	0,059	0,011	4.185e-08	0,064	0,035	0,066
rs870796	7	ELK1P1	G	A	0,017	0,003	3.274e-11	0,012	0,008	0,137
rs11782452	8	BNIP3L	G	A	0,015	0,003	1.258e-08	-0,001	0,008	0,947
rs12549853	8	PLEC	A	G	0,016	0,003	1.654e-09	-0,012	0,008	0,146
rs1431015	8	RNU2-54P	C	T	0,020	0,003	2.655e-14	-0,001	0,008	0,883
rs1495741	8	NAT2	G	A	0,026	0,003	5.034e-18	0,019	0,010	0,051
rs1786342	8	SNX31	T	C	0,017	0,003	1.241e-11	-0,009	0,008	0,265
rs2737205	8	TRPS1	T	C	0,023	0,003	4.711e-20	0,006	0,008	0,491
rs2978062	8	ST3GAL1	G	T	0,019	0,003	3.225e-08	0,002	0,011	0,841
rs445036	8	ZBTB10	T	C	0,019	0,003	5.794e-12	-0,008	0,009	0,380
rs56352849	8	KCNB2	A	G	0,016	0,003	1.497e-08	-0,021	0,009	0,014
rs60862542	8	EIF3E	G	A	0,017	0,003	1.648e-08	-0,011	0,009	0,247
rs6473015	8	AC105242.1	C	A	0,019	0,003	2.994e-12	0,028	0,009	0,001
rs716100	8	ZFAT	A	G	0,019	0,003	7.196e-13	0,001	0,009	0,868
rs76393968	8	MSR1	G	A	0,060	0,010	2.566e-09	0,027	0,045	0,541
rs9657541	8	CTD-2135J3.4	C	T	0,020	0,003	3.846e-10	0,020	0,010	0,040
rs1055710	9	FAM120AOS	G	A	0,018	0,003	7.149e-12	-0,016	0,008	0,047
rs10757291	9	CDKN2B-AS1	G	A	0,019	0,003	1.487e-14	0,014	0,008	0,073
rs10811787	9	RP11-370B11.4	C	T	0,015	0,003	7.833e-09	0,008	0,008	0,343
rs10869022	9	TRPM3	C	T	0,021	0,003	1.633e-11	-0,012	0,010	0,221
rs10908903	9	GADD45G	T	G	0,015	0,003	1.288e-09	-0,009	0,008	0,242
rs11557154	9	DCAF12	T	C	0,024	0,004	3.100e-10	0,012	0,012	0,306
rs13301073	9	MAPKAP1	G	A	0,022	0,003	4.056e-17	-0,001	0,008	0,880
rs2378662	9	RP11-158D2.2	G	A	0,017	0,003	4.195e-11	-0,014	0,008	0,072
rs28831479	9	PTCH1	C	A	0,022	0,003	1.196e-14	-0,010	0,009	0,271
rs7034716	9	TGFBR1	C	T	0,015	0,003	2.595e-08	-0,013	0,010	0,204
rs7041137	9	RAD23B	T	C	0,017	0,003	7.298e-10	-0,017	0,009	0,048
rs7872812	9	ASTN2	T	C	0,026	0,004	3.162e-13	0,001	0,011	0,930
rs10047326	10	PIP4K2A	A	C	0,017	0,003	1.263e-10	-0,002	0,008	0,804
rs10509746	10	Y_RNA	C	T	0,027	0,003	1.943e-26	0,005	0,008	0,510
rs10821713	10	ANK3	T	C	0,017	0,003	5.068e-11	0,012	0,008	0,142

rs11012712	10		C	T	0,022	0,003	3.752e-12	-0,010	0,010	0,313
rs116454156	10	FFAR4	A	G	0,078	0,010	1.615e-14	0,000	0,035	0,995
rs12244851	10	TCF7L2	T	C	0,015	0,003	1.450e-08	0,002	0,009	0,811
rs1832007	10	AKR1C4	G	A	0,057	0,003	6.779e-60	-0,006	0,011	0,577
rs2274224	10	PLCE1	G	C	0,024	0,003	1.017e-20	-0,018	0,008	0,023
rs2801482	10	CAMK1D	G	A	0,050	0,008	1.130e-09	0,033	0,025	0,193
rs293275	10	PRKG1	C	T	0,014	0,003	1.603e-08	-0,001	0,008	0,920
rs3858325	10	GFRA1	T	C	0,019	0,003	1.713e-13	0,009	0,008	0,277
rs4418728	10	CYP26A1	G	T	0,024	0,003	5.991e-21	0,009	0,008	0,273
rs4917962	10	NOLC1	T	G	0,024	0,004	5.216e-10	-0,018	0,012	0,130
rs7910087	10	C10orf11	T	C	0,017	0,003	6.229e-12	0,008	0,008	0,316
rs7921105	10	BEND7	C	T	0,016	0,003	1.962e-10	-0,004	0,008	0,587
rs9630085	10	FFAR4	G	A	0,022	0,003	9.144e-13	-0,011	0,010	0,283
rs1039481	11	PTPRJ	G	A	0,042	0,003	4.113e-48	-0,014	0,009	0,119
rs10767874	11	DCDC1	A	G	0,015	0,003	3.973e-08	0,022	0,008	0,008
rs10892564	11	ARHGEF12	G	A	0,017	0,003	4.611e-11	0,010	0,008	0,210
rs10893499	11	ST3GAL4	G	A	0,022	0,004	3.842e-09	0,005	0,012	0,655
rs11024614	11	HPS5	C	T	0,023	0,003	3.409e-18	0,000	0,008	0,967
rs11029620	11	NUP98	C	T	0,022	0,003	4.620e-13	-0,019	0,010	0,053
rs11031058	11	RPL12P30	T	C	0,022	0,003	5.389e-11	-0,010	0,011	0,347
rs117104648	11	AP5B1	C	T	0,036	0,005	3.240e-12	0,021	0,017	0,221
rs117600498	11	ASCL2	C	T	0,038	0,007	9.548e-09	-0,009	0,021	0,673
rs12790261	11	KDM2A	A	C	0,031	0,005	1.211e-11	-0,030	0,017	0,071
rs146345029	11	GIF	A	G	0,034	0,006	1.886e-08	0,001	0,019	0,979
rs174554	11	FADS1	A	G	0,022	0,003	7.202e-17	0,019	0,008	0,020
rs2512525	11	USP35	T	C	0,024	0,003	1.427e-12	-0,003	0,011	0,772
rs3213223	11	IGF2	A	G	0,076	0,003	9.35e-144	0,007	0,010	0,467
rs34452566	11	RP11-587D21.4	T	G	0,018	0,003	1.032e-08	0,011	0,010	0,260
rs35023999	11	ANKK1	C	A	0,015	0,003	1.160e-09	-0,010	0,008	0,189
rs4936759	11	C11orf63	C	T	0,016	0,003	1.949e-10	-0,017	0,008	0,033
rs4980661	11	AP000439.3	A	G	0,014	0,003	8.689e-09	-0,020	0,008	0,011
rs61867536	11	MOB2	T	C	0,018	0,003	1.132e-12	-0,001	0,008	0,873
rs61904289	11	EED	T	C	0,016	0,003	1.392e-09	0,007	0,008	0,432
rs625245	11	MRE11	G	T	0,016	0,003	4.364e-09	0,002	0,008	0,859
rs6485702	11	LRP4	T	C	0,017	0,003	1.415e-10	0,008	0,008	0,354
rs67257872	11	STK33	A	G	0,014	0,003	2.315e-08	0,004	0,008	0,642
rs7115466	11	H19	A	G	0,015	0,003	2.286e-08	0,004	0,010	0,660
rs72858776	11	RP11-396O20.2	G	T	0,030	0,005	1.479e-10	-0,027	0,014	0,059
rs7947951	11	ARNTL	G	A	0,020	0,003	1.025e-13	0,013	0,008	0,119
rs10745954	12	RP11-328J6.1	G	A	0,015	0,003	1.690e-09	0,016	0,008	0,039
rs10777540	12	CRADD	G	T	0,018	0,003	2.236e-12	-0,003	0,008	0,704
rs10841649	12	SLCO1B3	C	T	0,021	0,004	9.254e-09	-0,011	0,012	0,347
rs10860237	12	RP11-1016B18.1	A	G	0,030	0,003	2.391e-28	0,009	0,008	0,307
rs11064536	12	WNK1	T	C	0,020	0,003	2.941e-09	-0,002	0,011	0,891
rs11111274	12	IGF1	G	A	0,080	0,003	7.59e-175	-0,017	0,009	0,064

rs11175935	12	LRRK2	G	T	0,020	0,003	5.189e-10	0,011	0,010	0,272
rs117564283	12	ACVRL1	T	C	0,029	0,005	5.046e-09	-0,002	0,015	0,901
rs12231073	12	RNA5SP358	T	G	0,017	0,003	5.421e-12	0,014	0,008	0,072
rs12425869	12	SOCS2	A	G	0,018	0,003	3.957e-09	-0,011	0,010	0,262
rs1351394	12	HMGA2	C	T	0,024	0,003	6.924e-21	0,008	0,008	0,344
rs1800574	12	HNF1A	T	C	0,145	0,007	3.641e-84	0,024	0,025	0,322
rs2230281	12	GALNT4	A	G	0,016	0,003	4.010e-09	0,000	0,009	0,967
rs2460488	12	RP11-110L15.1	G	A	0,026	0,003	1.096e-14	-0,017	0,012	0,146
rs247917	12	ARID2	T	C	0,015	0,003	1.322e-09	-0,001	0,008	0,934
rs2657879	12	GLS2	A	G	0,020	0,003	9.621e-10	0,024	0,011	0,021
rs2856321	12	ETV6	G	A	0,026	0,003	1.583e-23	0,003	0,008	0,734
rs3759302	12	KIAA1551	A	T	0,021	0,003	6.949e-11	0,006	0,010	0,519
rs4547160	12	AVPR1A	T	G	0,018	0,003	3.168e-11	-0,012	0,008	0,148
rs7314285	12	CUX2	G	T	0,052	0,005	2.043e-25	-0,012	0,016	0,468
rs75938105	12	RP11-110L15.2	T	C	0,047	0,007	7.468e-12	-0,004	0,021	0,846
rs773116	12	ERBB3	G	A	0,016	0,003	1.265e-10	0,011	0,008	0,184
rs78607331	12	R3HDM2	C	T	0,037	0,006	6.059e-10	-0,002	0,021	0,933
rs9738365	12	RP11-428G5.4	A	C	0,058	0,003	1.905e-92	-0,007	0,009	0,473
rs1170158	13	DGKH	T	G	0,021	0,003	1.041e-10	-0,012	0,010	0,247
rs118081390	13	FNDC3A	G	A	0,028	0,005	1.353e-08	0,018	0,015	0,239
rs1535793	13	LRCH1	A	G	0,024	0,003	7.422e-17	0,004	0,009	0,635
rs6602909	13	GAS6	C	T	0,020	0,003	4.354e-14	0,003	0,009	0,728
rs71432868	13	SNORA25	C	T	0,028	0,005	3.105e-08	-0,013	0,019	0,477
rs7323205	13	LINC00676	C	T	0,015	0,003	9.354e-09	0,002	0,008	0,854
rs9532512	13	LINC00598	A	G	0,043	0,003	8.024e-40	0,000	0,010	0,975
rs9573360	13	LINC00402	A	C	0,014	0,003	1.597e-08	-0,002	0,008	0,771
rs9583151	13	AL354741.1	C	T	0,014	0,003	2.708e-08	0,000	0,008	0,963
rs10136874	14	DLK1	G	T	0,023	0,003	2.350e-20	-0,003	0,008	0,688
rs10145154	14	NRXN3	T	C	0,018	0,003	9.924e-10	0,020	0,009	0,029
rs1061638	14	AHSA1	G	A	0,018	0,003	7.484e-11	0,020	0,009	0,018
rs1115897	14	UNC79	A	C	0,021	0,003	1.540e-14	0,003	0,009	0,767
rs13379043	14	ELMSAN1	T	C	0,025	0,003	5.776e-18	0,001	0,009	0,881
rs168961	14	ZFP36L1	G	A	0,018	0,003	2.889e-13	0,014	0,008	0,082
rs17106640	14	ACTN1	G	A	0,017	0,003	8.431e-11	0,005	0,008	0,551
rs175043	14	EIF2B2	G	A	0,018	0,003	1.834e-12	-0,002	0,008	0,838
rs28396553	14	AL162511.1	T	C	0,015	0,003	2.962e-09	-0,004	0,008	0,626
rs28929474	14	SERPINA1	C	T	0,063	0,009	1.294e-12	-0,026	0,034	0,449
rs33912345	14	SIX6	C	A	0,023	0,003	2.363e-19	0,006	0,008	0,475
rs78598185	14	SLC24A4	G	A	0,029	0,004	7.090e-11	-0,023	0,015	0,120
rs79936318	14	SYNE2	A	G	0,017	0,003	3.875e-08	-0,031	0,011	0,006
rs8017377	14	NYNRIN	G	A	0,017	0,003	3.353e-11	0,000	0,008	0,975
rs11856160	15	CHD2	A	G	0,021	0,003	2.843e-09	0,028	0,010	0,007
rs12442867	15	RP11-299H22.1	A	C	0,017	0,003	9.329e-11	-0,001	0,008	0,920
rs12593755	15	RP11-97O12.3	T	G	0,016	0,003	1.846e-09	-0,009	0,008	0,242
rs12912439	15	LINC01197	T	C	0,022	0,003	1.246e-15	0,009	0,009	0,313

rs142354201	15	PGPEP1L	G	A	0,034	0,006	3.677e-09	0,012	0,019	0,519
rs17747633	15	KNL1	G	A	0,015	0,003	3.080e-09	-0,008	0,008	0,292
rs2004839	15	RP11-299H22.3	G	A	0,021	0,003	4.336e-10	-0,004	0,011	0,727
rs2311313	15	RP11-35O15.1	G	T	0,019	0,003	1.691e-08	0,005	0,011	0,660
rs2930313	15	CCDC33	G	A	0,028	0,005	4.141e-09	-0,002	0,015	0,871
rs4545755	15	CYP19A1	G	A	0,016	0,003	3.865e-10	0,006	0,008	0,439
rs55707100	15	MAP1A	C	T	0,151	0,008	1.406e-76	0,021	0,025	0,404
rs5742915	15	PML	C	T	0,025	0,003	1.723e-22	-0,002	0,008	0,821
rs79076440	15	USP3	A	G	0,019	0,003	1.164e-08	-0,012	0,010	0,249
rs8033075	15	PIAS1	A	G	0,045	0,005	1.061e-17	0,013	0,017	0,445
rs11077337	16	ZNF597	T	G	0,015	0,003	1.454e-09	-0,018	0,008	0,026
rs11149612	16	RP11-505K9.4	C	T	0,027	0,003	1.131e-26	-0,006	0,008	0,499
rs111792934	16	HAS3	C	T	0,022	0,003	1.430e-10	-0,010	0,010	0,332
rs116971887	16	SALL1	G	T	0,036	0,006	4.895e-09	-0,028	0,020	0,148
rs12597502	16	CHD9	G	A	0,015	0,003	4.528e-08	-0,007	0,009	0,400
rs12927172	16	IL4R	A	G	0,015	0,003	3.066e-09	0,020	0,009	0,034
rs12935091	16	ZNF19	G	A	0,035	0,006	2.235e-09	-0,013	0,019	0,509
rs12935465	16	XYLT1	T	C	0,016	0,003	5.769e-11	-0,015	0,008	0,056
rs147491123	16	LINC01572	C	T	0,036	0,007	3.286e-08	-0,036	0,021	0,094
rs1532824	16	ATF7IP2	A	C	0,017	0,003	4.521e-09	0,006	0,009	0,485
rs1548917	16	RP11-461O7.1	T	C	0,015	0,003	2.361e-09	-0,001	0,008	0,952
rs1657125	16	MEIOB	T	G	0,032	0,004	2.007e-18	-0,006	0,012	0,621
rs17299478	16	NOB1	C	T	0,032	0,003	2.497e-21	-0,017	0,011	0,105
rs2023762	16	SYT17	T	C	0,015	0,003	5.991e-09	0,001	0,008	0,947
rs4786350	16	IFT140	C	G	0,036	0,006	6.048e-09	0,030	0,019	0,127
rs4788220	16	FAM57B	G	A	0,017	0,003	5.263e-12	0,010	0,008	0,225
rs4985062	16	USP7	T	C	0,015	0,003	2.040e-09	-0,002	0,008	0,808
rs61731445	16		C	T	0,028	0,005	1.958e-08	0,005	0,016	0,785
rs7204824	16	LMF1	C	T	0,024	0,003	1.168e-16	-0,018	0,009	0,058
rs72761177	16	NUBP2	A	G	0,077	0,004	7.205e-70	0,015	0,014	0,283
rs74774288	16	RP11-420N3.3	G	T	0,027	0,003	2.955e-17	0,002	0,010	0,805
rs7498665	16	SH2B1	G	A	0,019	0,003	7.173e-14	0,013	0,008	0,102
rs750952	16	ZNF646	C	T	0,032	0,003	1.105e-34	-0,006	0,008	0,457
rs753108	16	CMIP	A	G	0,025	0,003	1.606e-17	0,000	0,009	0,980
rs80253441	16	IGFALS	T	C	0,131	0,011	8.227e-36	-0,037	0,034	0,273
rs8054054	16	CCDC154	G	A	0,015	0,003	1.026e-09	0,006	0,008	0,489
rs8054322	16	GSE1	A	G	0,015	0,003	4.723e-09	-0,003	0,008	0,682
rs8059803	16	CMIP	A	G	0,031	0,003	4.415e-29	0,010	0,009	0,263
rs8182173	16	CORO7-PAM16	T	C	0,018	0,003	1.610e-09	0,005	0,009	0,604
rs142377191	17	DCAF7	A	G	0,125	0,009	2.048e-46	-0,017	0,033	0,604
rs1801689	17	APOH	C	A	0,089	0,007	1.248e-33	0,078	0,027	0,004
rs199525	17	WNT3	G	T	0,020	0,003	5.613e-11	0,015	0,010	0,141
rs2309401	17	NLRP1	T	G	0,015	0,003	6.785e-09	0,011	0,008	0,163
rs35819807	17	KCNH6	T	C	0,017	0,003	2.387e-09	0,011	0,009	0,220
rs3760237	17	SCN4A	C	T	0,024	0,003	1.441e-21	-0,006	0,008	0,455

rs4075483	17	BAIAP2	C	T	0,017	0,003	1.312e-10	0,019	0,008	0,023
rs4789227	17	UNK	T	C	0,015	0,003	4.335e-09	0,017	0,008	0,045
rs56030650	17	GSDMA	C	A	0,022	0,003	2.417e-18	-0,012	0,008	0,147
rs6416868	17	TTC19	A	G	0,019	0,003	3.115e-14	-0,018	0,008	0,024
rs6501601	17	POLR3KP2	G	A	0,015	0,003	4.071e-09	-0,001	0,008	0,901
rs668799	17	COASY	C	T	0,018	0,003	4.019e-10	0,005	0,009	0,599
rs7502910	17	WDR81	A	G	0,016	0,003	1.528e-10	0,007	0,008	0,372
rs76708468	17	1ERN	C	T	0,087	0,006	3.084e-41	-0,063	0,028	0,021
rs77542162	17	ABCA6	G	A	0,054	0,009	2.412e-10	-0,008	0,030	0,790
rs8075153	17	RAI1	C	T	0,021	0,003	1.831e-17	0,017	0,008	0,028
rs8079923	17	AKAP10	C	T	0,016	0,003	3.664e-08	-0,011	0,009	0,251
rs9892862	17	Y_RNA	G	A	0,022	0,003	9.705e-14	0,023	0,011	0,041
rs11152071	18	RP11-1151B14.2	C	T	0,020	0,003	8.957e-12	-0,011	0,009	0,240
rs12454712	18	BCL2	T	C	0,018	0,003	4.051e-12	0,011	0,009	0,217
rs190102446	18	RP11-27G24.1	C	T	0,041	0,007	2.452e-09	0,011	0,021	0,591
rs57551555	18	RP11-176N18.2	T	G	0,019	0,003	5.817e-13	0,013	0,008	0,116
rs585187	18	MRPS5P4	T	G	0,015	0,003	3.712e-09	-0,012	0,008	0,147
rs8084351	18	DCC	G	A	0,015	0,003	8.537e-10	-0,005	0,008	0,551
rs8095538	18	-	G	T	0,020	0,003	8.447e-14	-0,004	0,009	0,617
rs8097893	18	GALR1	A	G	0,058	0,006	1.723e-20	0,002	0,019	0,920
rs11671304	19	ZC3H4	C	T	0,018	0,003	6.884e-11	-0,018	0,009	0,040
rs12975366	19	LILRB5	T	C	0,020	0,003	2.253e-15	-0,012	0,009	0,163
rs2287922	19	RASIP1	G	A	0,030	0,003	1.833e-33	-0,017	0,008	0,031
rs296361	19	SULT2A1	A	G	0,025	0,003	3.421e-13	0,018	0,011	0,100
rs34536443	19	TYK2	G	C	0,045	0,006	9.964e-14	0,020	0,021	0,344
rs3760954	19	MIR7-3HG	T	C	0,024	0,004	2.909e-10	-0,009	0,012	0,461
rs58560372	19	SPINT2	C	T	0,020	0,003	1.331e-08	-0,006	0,011	0,615
rs58658292	19	ZNF536	G	A	0,030	0,006	4.618e-08	0,017	0,018	0,361
rs62102136	19	LSM14A	C	T	0,016	0,003	6.527e-09	0,003	0,008	0,691
rs6510033	19	AC005597.1	A	G	0,020	0,003	1.147e-12	0,005	0,009	0,576
rs6510177	19	ZNF536	T	C	0,023	0,003	1.598e-12	0,004	0,011	0,697
rs6510832	19	KDM4B	G	T	0,033	0,005	2.638e-10	0,005	0,015	0,738
rs67868323	19	ZBTB7A	T	G	0,016	0,003	2.205e-08	0,004	0,009	0,675
rs7254601	19	COX6B1	G	A	0,016	0,003	2.551e-08	0,004	0,009	0,699
rs7256521	19	ZNF845	G	A	0,015	0,003	9.761e-10	-0,014	0,008	0,084
rs8105174	19	DNMT1	C	T	0,050	0,003	7.971e-54	0,016	0,011	0,138
rs8112883	19	INSR	G	T	0,017	0,003	1.166e-09	-0,015	0,009	0,076
rs8113618	19	QTRT1	T	C	0,031	0,003	1.185e-33	0,002	0,008	0,826
rs16995311	20	PTPN1	A	C	0,040	0,005	5.559e-18	0,020	0,015	0,175
rs17265513	20	ZHX3	T	C	0,022	0,003	3.454e-12	0,004	0,010	0,673
rs2207132	20	LINC01728	G	A	0,048	0,007	1.064e-11	-0,012	0,024	0,602
rs2424396	20	LINC01726	G	A	0,033	0,004	2.245e-14	0,008	0,013	0,523
rs2738787	20	TNFRSF6B	A	G	0,037	0,005	1.251e-15	0,023	0,015	0,119
rs4809401	20	NPBWR2	T	C	0,023	0,004	6.488e-10	-0,010	0,012	0,409
rs6037508	20	SLC4A11	G	T	0,017	0,003	8.740e-09	0,013	0,011	0,210

rs6046825	20	RALGAPA2	A	C	0,024	0,003	2.108e-17	0,003	0,009	0,724
rs6088579	20	NCOA6	G	A	0,027	0,003	8.509e-17	0,011	0,011	0,307
rs7267595	20	JAG1	A	C	0,015	0,003	9.437e-10	0,011	0,008	0,159
rs7508949	20	CRNKL1	C	G	0,025	0,003	1.490e-22	0,016	0,008	0,048
rs75989562	20	APMAP	A	G	0,030	0,005	8.093e-09	0,034	0,018	0,061
rs9978775	21	BRWD1-AS1	G	A	0,019	0,003	2.351e-13	-0,012	0,008	0,117
rs12106594	22	EIF4ENIF1	T	C	0,036	0,006	1.588e-09	0,033	0,018	0,059
rs2412973	22	HORMAD2	A	C	0,014	0,003	3.334e-08	0,003	0,008	0,684
rs4823324	22	ATXN10	T	C	0,016	0,003	1.938e-10	0,007	0,008	0,401
rs5755948	22	RBFOX2	A	G	0,028	0,004	4.375e-14	0,013	0,011	0,258
rs6519133	22	JOSD1	T	C	0,029	0,003	7.731e-30	-0,004	0,008	0,612
rs8138950	22	ZNRF3	C	T	0,015	0,003	1.323e-09	0,006	0,008	0,435
rs9611565	22	TEF	T	C	0,029	0,003	3.825e-23	-0,010	0,009	0,304

Chr, chromosome; EA, effect allele; OA, other allele; SE, standard error.

ESM Table 4. Summary statistics of the single-nucleotide polymorphisms associated with insulin-like growth factor-1 and their associations with atrial fibrillation

					**IGF-1**			**Atrial fibrillation**		
**SNP**	**Chr**	**Nearby gene**	**EA**	**OA**	**Beta**	**SE**	***p***	**Beta**	**SE**	***p***
rs10159299	1	PLXNA2	C	T	0,018	0,003	5.112e-12	-0,007	0,007	0,322
rs1046011	1	LEPROT	T	C	0,021	0,003	3.303e-14	0,005	0,008	0,517
rs10779509	1	RP1-272L16.1	C	T	0,014	0,003	2.109e-08	0,001	0,007	0,933
rs11165778	1	HFM1	A	G	0,016	0,003	1.492e-08	-0,004	0,008	0,678
rs112436634	1	PEX14	T	C	0,016	0,003	1.749e-09	0,022	0,008	0,004
rs1127313	1	ADAR	G	A	0,024	0,003	1.394e-21	0,011	0,007	0,129
rs11577063	1	AXDND1	T	G	0,020	0,003	1.511e-11	0,003	0,009	0,744
rs12141189	1	HLX	T	C	0,045	0,003	4.135e-53	0,005	0,009	0,522
rs1223763	1	RP11-53A1.3	T	G	0,024	0,003	8.461e-13	0,017	0,009	0,064
rs12723255	1	EIF4G3	C	T	0,017	0,003	3.229e-11	-0,001	0,007	0,887
rs12749024	1	PAPPA2	T	C	0,075	0,004	7.72e-100	-0,004	0,011	0,685
rs140604451	1	GSTM2	G	A	0,054	0,008	3.524e-11	-0,023	0,024	0,340
rs1430753	1	WLS	A	G	0,021	0,003	7.085e-11	0,001	0,009	0,944
rs143885630	1	SMG7	G	A	0,030	0,004	1.358e-15	-0,014	0,011	0,204
rs165316	1	RPL5P6	G	A	0,073	0,003	9.66e-119	0,009	0,009	0,334
rs17037452	1	CLCN6	A	G	0,023	0,003	1.658e-11	-0,028	0,010	0,004
rs17393144	1	MIR34AHG	A	G	0,016	0,003	9.319e-09	0,000	0,008	0,960
rs1825813	1	C1orf146	A	G	0,023	0,003	1.879e-13	0,005	0,009	0,608
rs2075995	1	E2F2	C	A	0,014	0,003	9.790e-09	-0,016	0,007	0,021
rs2724373	1	C1orf132	C	T	0,019	0,003	1.436e-12	0,012	0,008	0,138
rs2802330	1	PDIK1L	G	A	0,031	0,003	4.632e-21	-0,009	0,009	0,318
rs2802951	1	RN7SL668P	A	G	0,016	0,003	4.970e-09	-0,011	0,008	0,184
rs2819336	1	PTPRF	T	C	0,027	0,003	1.550e-25	-0,001	0,008	0,848
rs3131646	1	MYCL	G	T	0,016	0,003	1.710e-08	0,011	0,008	0,167
rs36086195	1	ARHGEF19-AS1	C	T	0,019	0,003	1.359e-13	0,004	0,007	0,572
rs4306136	1	RP11-103C3.1	A	G	0,017	0,003	1.997e-11	-0,001	0,007	0,895
rs569356	1	OPRD1	G	A	0,027	0,004	5.953e-14	0,003	0,011	0,753
rs599839	1	CELSR2	G	A	0,031	0,003	9.629e-26	-0,016	0,009	0,060
rs61780439	1	SLFNL1	G	A	0,021	0,003	5.323e-12	-0,034	0,009	0,000
rs6659176	1	NR0B2	C	G	0,042	0,005	1.354e-19	-0,002	0,014	0,900
rs6701954	1	USP48	T	G	0,014	0,003	3.497e-08	0,001	0,007	0,920
rs684818	1	RP4-781K5.7	T	C	0,024	0,003	9.658e-21	-0,004	0,007	0,600
rs708108	1	WNT3A	T	C	0,015	0,003	5.872e-09	-0,015	0,008	0,053
rs7517340	1	AKT3	C	T	0,035	0,003	2.123e-26	-0,030	0,009	0,001
rs7528548	1	PYGO2	C	T	0,054	0,009	7.954e-10	0,020	0,025	0,415
rs7539178	1	JAK1	C	A	0,026	0,004	1.365e-12	-0,004	0,010	0,672
rs7545345	1	NUCKS1	C	T	0,026	0,004	1.032e-12	0,036	0,011	0,001
rs75681856	1	RABGAP1L	T	C	0,023	0,004	5.171e-09	0,020	0,011	0,064
rs75907879	1	SPEN	T	C	0,024	0,004	1.425e-10	0,009	0,013	0,464
rs77369503	1	RGS4	G	A	0,045	0,007	1.397e-10	-0,035	0,024	0,148

rs903908	1	SKI	C	T	0,016	0,003	2.176e-10	0,002	0,007	0,777
rs11677980	2	LBH	G	A	0,015	0,003	3.043e-08	0,001	0,008	0,897
rs11678946	2	EPHA4	A	C	0,014	0,003	3.143e-08	0,004	0,007	0,577
rs12471768	2	SERTAD2	C	T	0,022	0,003	1.639e-15	-0,002	0,008	0,836
rs1260326	2	GCKR	C	T	0,063	0,003	9.66e-133	0,007	0,007	0,340
rs12710648	2	SMC6	A	G	0,017	0,003	5.703e-12	0,009	0,008	0,225
rs1465529	2	SP110	T	C	0,019	0,003	1.386e-12	0,014	0,008	0,083
rs17050272	2	AC073257.2	G	A	0,024	0,003	3.201e-20	0,010	0,007	0,172
rs17323117	2	NEU2	G	A	0,029	0,005	7.980e-10	-0,009	0,014	0,515
rs17400325	2	PDE11A	C	T	0,054	0,006	9.393e-17	0,065	0,020	0,001
rs2674492	2	CYBRD1	A	G	0,014	0,003	4.835e-08	-0,002	0,007	0,824
rs35135518	2	AC010145.4	T	C	0,029	0,004	1.521e-12	0,013	0,011	0,254
rs35641591	2	PCBP1-AS1	C	T	0,050	0,006	1.575e-14	0,041	0,023	0,076
rs3791679	2	EFEMP1	G	A	0,018	0,003	4.338e-09	-0,028	0,008	0,001
rs4402747	2	NEU2	A	G	0,016	0,003	2.960e-10	0,011	0,007	0,112
rs58387407	2	CACNB4	G	A	0,018	0,003	3.502e-08	-0,013	0,009	0,141
rs62136965	2	RNU6-566P	C	T	0,037	0,006	6.429e-10	0,000	0,019	0,992
rs62182127	2	VIL1	A	G	0,019	0,003	2.712e-14	0,002	0,007	0,819
rs6435156	2	BMPR2	C	T	0,024	0,003	2.045e-17	-0,007	0,008	0,429
rs6437249	2	HDLBP	C	T	0,019	0,003	4.559e-12	-0,021	0,008	0,008
rs6544549	2	KCNG3	T	C	0,024	0,004	1.592e-10	0,015	0,011	0,168
rs6749680	2	ALMS1	A	G	0,015	0,003	9.868e-09	-0,020	0,007	0,006
rs6760135	2	ASXL2	T	C	0,050	0,003	1.194e-63	0,033	0,009	0,000
rs702878	2	AC074391.1	A	G	0,014	0,003	1.815e-08	-0,005	0,007	0,541
rs73954943	2	BCL2L11	A	G	0,031	0,005	1.063e-09	0,012	0,014	0,392
rs7574340	2	SLC8A1	T	C	0,017	0,003	2.971e-10	-0,002	0,008	0,791
rs7578633	2	PAX8	T	C	0,018	0,003	3.379e-12	-0,010	0,007	0,168
rs112893170	3	FEZF2	T	C	0,020	0,003	1.527e-10	-0,031	0,010	0,001
rs11717397	3	UBE2E2	G	A	0,015	0,003	2.572e-09	-0,006	0,007	0,438
rs11928797	3	UBP1	A	C	0,030	0,004	2.428e-14	-0,011	0,012	0,358
rs12491473	3	CCDC12	G	A	0,020	0,003	5.913e-15	-0,016	0,007	0,029
rs13069961	3	KALRN	G	A	0,018	0,003	3.680e-09	-0,013	0,009	0,140
rs13073970	3	EIF5A2	T	G	0,025	0,003	2.016e-15	0,014	0,009	0,112
rs1822825	3	PPARG	G	A	0,014	0,003	2.886e-08	0,011	0,007	0,132
rs2268829	3	DGKG	G	A	0,018	0,003	3.038e-09	-0,017	0,009	0,050
rs2607748	3	CHCHD4	C	T	0,017	0,003	3.289e-11	0,019	0,007	0,010
rs3772102	3	ST3GAL6	G	T	0,020	0,003	5.954e-16	-0,005	0,007	0,521
rs4678497	3	SUSD5	C	T	0,017	0,003	1.959e-10	-0,018	0,007	0,015
rs504603	3	BZW1P1	C	T	0,029	0,005	2.402e-09	0,003	0,014	0,823
rs55717031	3	MRPS22	G	T	0,032	0,003	2.474e-31	0,007	0,008	0,399
rs56062334	3	LINC02068	T	C	0,017	0,003	1.839e-11	0,008	0,008	0,266
rs62263345	3	BBX	A	G	0,028	0,004	1.640e-14	-0,012	0,011	0,284
rs62280667	3	SENP7	C	T	0,028	0,003	2.477e-26	-0,004	0,008	0,589
rs6440008	3	ZBTB38	T	C	0,035	0,003	1.113e-41	-0,020	0,007	0,007
rs66707192	3	HRG	G	A	0,018	0,003	4.415e-10	-0,001	0,008	0,865

rs687339	3	KRT18P35	T	C	0,040	0,003	2.666e-40	0,027	0,009	0,001
rs73238159	3	XRN1	C	T	0,025	0,004	1.059e-11	-0,029	0,010	0,006
rs7625680	3	ATG7	A	G	0,015	0,003	7.693e-09	0,009	0,007	0,220
rs7628689	3	C3orf38	G	A	0,029	0,003	6.825e-17	-0,006	0,010	0,519
rs811332	3	MRAS	C	T	0,019	0,003	2.011e-09	-0,006	0,009	0,554
rs9819762	3	PIK3CA	T	C	0,019	0,003	1.212e-08	0,006	0,009	0,524
rs1055582	4	UBE2K	C	T	0,027	0,003	1.578e-27	0,001	0,007	0,931
rs111443396	4	LINC01091	C	T	0,026	0,004	2.015e-10	0,008	0,011	0,463
rs1229984	4	ADH1B	T	C	0,104	0,009	1.219e-34	-0,023	0,019	0,216
rs13108218	4	HGFAC	G	A	0,017	0,003	5.920e-11	0,010	0,007	0,177
rs17429745	4	RP11-556I14.1	G	T	0,026	0,003	2.045e-21	-0,002	0,008	0,827
rs1902023	4	UGT2B15	A	C	0,025	0,003	1.951e-23	-0,002	0,008	0,850
rs2280099	4	TIGD2	G	A	0,025	0,003	1.923e-14	0,000	0,009	0,984
rs35036084	4	RP11-145G20.1	T	C	0,017	0,003	8.218e-11	0,016	0,008	0,033
rs3804173	4	PRDM5	G	A	0,020	0,003	4.484e-13	-0,004	0,008	0,607
rs4394044	4	SORBS2	T	C	0,014	0,003	4.708e-08	-0,006	0,007	0,395
rs62302688	4	GABRA2	G	A	0,039	0,004	4.057e-20	0,004	0,014	0,771
rs62334147	4	DDX60L	C	T	0,019	0,003	4.261e-09	0,002	0,010	0,800
rs62342064	4	RP11-119H12.3	C	T	0,022	0,004	1.836e-09	0,004	0,012	0,732
rs6532798	4	ADH4	T	C	0,037	0,003	1.364e-41	-0,004	0,008	0,610
rs6827641	4	HHIP	T	C	0,014	0,003	2.623e-08	-0,015	0,007	0,031
rs6853741	4	ARHGAP10	A	G	0,024	0,003	1.676e-16	-0,011	0,008	0,167
rs7667562	4	LARP1B	C	A	0,016	0,003	3.770e-09	0,013	0,008	0,098
rs976002	4	TMPRSS11E	A	G	0,036	0,003	4.672e-35	-0,003	0,009	0,734
rs11242236	5	C5orf66	G	A	0,025	0,003	1.957e-22	0,002	0,007	0,807
rs11954036	5	PDE4D	T	C	0,037	0,003	1.936e-44	0,003	0,008	0,706
rs12108803	5	TBCA	G	T	0,033	0,006	1.015e-08	0,030	0,017	0,072
rs12520263	5	RP11-357F12.1	T	G	0,017	0,003	2.579e-09	-0,003	0,008	0,664
rs13168379	5	CPEB4	A	G	0,031	0,005	6.148e-10	0,022	0,012	0,074
rs13178887	5	MEF2C-AS1	T	C	0,023	0,003	2.712e-19	0,021	0,007	0,004
rs1498603	5	PDE4D	T	G	0,031	0,005	1.170e-09	-0,027	0,015	0,065
rs17714046	5	TRIM41	C	T	0,042	0,006	1.197e-12	-0,030	0,019	0,117
rs2042253	5	MIR5197	T	C	0,023	0,003	1.017e-14	0,013	0,008	0,112
rs2227819	5	F2R	T	C	0,022	0,004	4.322e-08	0,033	0,012	0,006
rs2366398	5	CTD-2151A2.3	T	G	0,018	0,003	1.613e-09	0,010	0,009	0,233
rs258775	5	ARHGAP26	A	C	0,025	0,003	3.394e-14	-0,013	0,009	0,151
rs26822	5	PPIP5K2	G	A	0,017	0,003	9.845e-11	0,009	0,008	0,262
rs28650790	5	C5orf67	T	C	0,018	0,003	4.108e-08	0,007	0,009	0,454
rs329122	5	JADE2	A	G	0,018	0,003	1.312e-12	-0,016	0,007	0,027
rs35668185	5	SLIT3	T	C	0,056	0,003	3.402e-73	0,010	0,009	0,279
rs3734166	5	CDC25C	A	G	0,028	0,003	8.063e-22	0,000	0,008	0,966
rs6180	5	GHR	A	C	0,035	0,003	6.019e-43	-0,008	0,007	0,239
rs6895953	5	RP11-357F12.1	G	A	0,024	0,003	5.167e-21	-0,017	0,007	0,020
rs72758321	5	PLCXD3	G	A	0,047	0,006	3.926e-15	-0,004	0,021	0,843
rs73271090	5	CTB-1I21.1	G	A	0,044	0,003	7.844e-39	-0,011	0,010	0,277

rs7719168	5	15ARL	C	A	0,030	0,004	2.429e-14	0,016	0,012	0,171
rs80170948	5	SREK1IP1	G	T	0,039	0,006	2.221e-09	0,001	0,021	0,959
rs840809	5	CTD-2232E5.2	A	C	0,016	0,003	1.183e-08	0,004	0,008	0,598
rs9292578	5	PRLR	C	A	0,040	0,006	3.195e-10	-0,008	0,018	0,655
rs1042335	6	HLA-DPB1	T	C	0,015	0,003	3.568e-08	0,002	0,008	0,811
rs1130838	6	HLA-C	C	T	0,026	0,003	1.848e-23	-0,019	0,008	0,015
rs113127944	6	CENPW	A	G	0,051	0,008	6.115e-11	-0,012	0,026	0,658
rs1150781	6	C6orf1	G	C	0,026	0,004	2.337e-09	-0,047	0,012	0,000
rs1165196	6	SLC17A1	G	A	0,029	0,003	6.414e-30	0,010	0,007	0,156
rs12110787	6	MAP3K4	A	C	0,022	0,004	1.857e-08	0,013	0,011	0,240
rs12194618	6	ZFAND3	A	G	0,017	0,003	2.686e-11	0,000	0,008	0,993
rs2296198	6	RNF144B	C	T	0,016	0,003	1.937e-08	0,028	0,008	0,001
rs2397112	6	RP11-228O6.2	A	G	0,019	0,003	3.313e-13	-0,007	0,008	0,327
rs28362677	6	BTNL2	T	C	0,032	0,004	1.763e-19	-0,008	0,010	0,391
rs3008051	6	PDE10A	C	T	0,014	0,003	1.946e-08	0,014	0,008	0,058
rs3127579	6	SLC22A2	A	G	0,033	0,004	1.230e-19	-0,005	0,011	0,612
rs3890746	6	L3MBTL3	C	T	0,020	0,003	1.060e-15	0,012	0,007	0,098
rs41285260	6	CENPW	T	G	0,039	0,004	5.123e-18	-0,001	0,014	0,960
rs4709995	6	SDIM1	T	C	0,042	0,003	7.369e-60	0,012	0,007	0,112
rs584955	6	TMEM14C	A	G	0,036	0,006	2.579e-09	-0,043	0,017	0,013
rs670049	6	Y_RNA	A	C	0,019	0,003	6.417e-13	0,012	0,008	0,117
rs6916994	6	GJB7	C	T	0,029	0,003	1.519e-31	0,039	0,007	0,000
rs6924225	6	RUNX2	G	A	0,019	0,003	1.442e-08	-0,016	0,010	0,117
rs73382439	6	E2F3	C	T	0,019	0,003	1.800e-08	0,017	0,009	0,061
rs7740433	6	CNPY3	A	G	0,017	0,003	1.504e-08	0,009	0,009	0,283
rs7758644	6	RP1-155D22.1	A	C	0,019	0,003	1.514e-08	-0,002	0,010	0,825
rs7774230	6	ESR1	T	C	0,026	0,003	5.464e-25	0,009	0,007	0,203
rs790513	6	OPRM1	C	A	0,025	0,003	1.575e-18	-0,007	0,008	0,407
rs9321106	6	PTPRK	A	G	0,018	0,003	2.408e-08	0,007	0,010	0,458
rs9322822	6	LIN28B-AS1	C	T	0,015	0,003	1.349e-08	-0,013	0,008	0,080
rs9364815	6	PDE10A	A	G	0,015	0,003	2.829e-08	-0,001	0,008	0,894
rs9398171	6	FOXO3	T	C	0,050	0,003	9.510e-74	-0,018	0,008	0,022
rs9398891	6	LAMA2	T	C	0,017	0,003	1.108e-10	-0,013	0,008	0,102
rs998584	6	VEGFA	A	C	0,020	0,003	1.211e-15	-0,018	0,007	0,015
rs10246481	7	AC073133.2	G	A	0,015	0,003	1.499e-08	-0,006	0,007	0,397
rs10252510	7	GHRHR	G	A	0,020	0,003	8.179e-14	-0,006	0,008	0,486
rs1050327	7	ZMIZ2	A	G	0,017	0,003	2.907e-11	0,018	0,007	0,012
rs114949263	7	TMEM176B	T	C	0,027	0,004	1.283e-11	0,043	0,012	0,000
rs11556924	7	ZC3HC1	C	T	0,016	0,003	9.798e-10	0,004	0,008	0,577
rs1182174	7	GNA12	A	G	0,021	0,003	4.808e-14	-0,035	0,008	0,000
rs12666306	7	RP11-222O23.1	G	A	0,017	0,003	1.702e-11	0,009	0,007	0,225
rs12699547	7	MAD1L1	C	T	0,021	0,003	2.438e-16	-0,003	0,007	0,662
rs145188037	7	IGFBP3	A	G	0,120	0,011	1.749e-27	-0,019	0,039	0,627
rs17145738	7	TBL2	T	C	0,034	0,004	8.750e-19	0,009	0,011	0,433
rs1986692	7	EXOC4	G	A	0,015	0,003	8.640e-09	-0,001	0,007	0,894

rs2048672	7	LINC-PINT	C	A	0,018	0,003	6.405e-11	0,002	0,008	0,814
rs207212	7	LINC00513	C	T	0,028	0,004	1.659e-11	-0,001	0,014	0,918
rs2228078	7	GHRHR	C	T	0,057	0,010	4.076e-08	-0,032	0,036	0,369
rs2250243	7	ZNF316	C	T	0,024	0,003	1.694e-15	-0,021	0,008	0,011
rs2270628	7	IGFBP3	T	C	0,033	0,003	3.737e-25	0,007	0,009	0,444
rs273956	7	CREB3L2	A	G	0,021	0,003	5.369e-16	-0,011	0,007	0,154
rs2896395	7	SND1	C	T	0,015	0,003	2.446e-08	-0,012	0,008	0,129
rs34312198	7	ZNF3	A	C	0,024	0,004	1.345e-09	-0,004	0,012	0,721
rs35862187	7	AUTS2	A	G	0,031	0,006	3.484e-08	-0,016	0,017	0,348
rs411717	7	COL1A2	T	C	0,015	0,003	2.144e-09	-0,001	0,007	0,926
rs4719393	7	DGKB	T	G	0,027	0,003	1.290e-22	0,015	0,008	0,047
rs74657816	7	HMGN1P19	T	G	0,047	0,005	3.006e-18	-0,012	0,016	0,450
rs7783012	7	FOXP2	G	A	0,016	0,003	1.561e-10	-0,002	0,007	0,815
rs7802508	7	ZFAND2A	A	G	0,021	0,003	9.477e-17	-0,001	0,007	0,900
rs79881512	7	AC073325.1	C	T	0,059	0,011	4.185e-08	0,049	0,034	0,144
rs870796	7	ELK1P1	G	A	0,017	0,003	3.274e-11	0,001	0,007	0,867
rs11782452	8	BNIP3L	G	A	0,015	0,003	1.258e-08	0,003	0,007	0,667
rs12549853	8	PLEC	A	G	0,016	0,003	1.654e-09	-0,010	0,007	0,197
rs1431015	8	RNU2-54P	C	T	0,020	0,003	2.655e-14	0,014	0,007	0,046
rs1495741	8	NAT2	G	A	0,026	0,003	5.034e-18	0,015	0,008	0,070
rs1786342	8	SNX31	T	C	0,017	0,003	1.241e-11	-0,009	0,007	0,210
rs2737205	8	TRPS1	T	C	0,023	0,003	4.711e-20	0,002	0,007	0,796
rs2978062	8	ST3GAL1	G	T	0,019	0,003	3.225e-08	0,010	0,010	0,321
rs445036	8	ZBTB10	T	C	0,019	0,003	5.794e-12	0,004	0,008	0,599
rs56352849	8	KCNB2	A	G	0,016	0,003	1.497e-08	-0,003	0,008	0,715
rs60862542	8	EIF3E	G	A	0,017	0,003	1.648e-08	-0,001	0,009	0,936
rs6473015	8	AC105242.1	C	A	0,019	0,003	2.994e-12	0,022	0,008	0,004
rs716100	8	ZFAT	A	G	0,019	0,003	7.196e-13	-0,009	0,008	0,264
rs76393968	8	MSR1	G	A	0,060	0,010	2.566e-09	-0,008	0,030	0,799
rs9657541	8	CTD-2135J3.4	C	T	0,020	0,003	3.846e-10	0,002	0,009	0,872
rs1055710	9	FAM120AOS	G	A	0,018	0,003	7.149e-12	-0,013	0,007	0,092
rs10757291	9	CDKN2B-AS1	G	A	0,019	0,003	1.487e-14	0,010	0,007	0,178
rs10811787	9	RP11-370B11.4	C	T	0,015	0,003	7.833e-09	0,002	0,007	0,791
rs10869022	9	TRPM3	C	T	0,021	0,003	1.633e-11	0,010	0,009	0,279
rs10908903	9	GADD45G	T	G	0,015	0,003	1.288e-09	0,009	0,007	0,216
rs11557154	9	DCAF12	T	C	0,024	0,004	3.100e-10	-0,004	0,010	0,673
rs13301073	9	MAPKAP1	G	A	0,022	0,003	4.056e-17	0,009	0,008	0,260
rs2378662	9	RP11-158D2.2	G	A	0,017	0,003	4.195e-11	-0,008	0,007	0,270
rs28831479	9	PTCH1	C	A	0,022	0,003	1.196e-14	-0,005	0,008	0,564
rs7034716	9	TGFBR1	C	T	0,015	0,003	2.595e-08	-0,014	0,008	0,093
rs7041137	9	RAD23B	T	C	0,017	0,003	7.298e-10	0,003	0,008	0,720
rs7872812	9	ASTN2	T	C	0,026	0,004	3.162e-13	0,012	0,010	0,258
rs10047326	10	PIP4K2A	A	C	0,017	0,003	1.263e-10	-0,017	0,007	0,020
rs10509746	10	Y_RNA	C	T	0,027	0,003	1.943e-26	0,005	0,007	0,546
rs10821713	10	ANK3	T	C	0,017	0,003	5.068e-11	0,015	0,007	0,037

rs11012712	10		C	T	0,022	0,003	3.752e-12	0,004	0,009	0,676
rs116454156	10	FFAR4	A	G	0,078	0,010	1.615e-14	-0,028	0,034	0,408
rs12244851	10	TCF7L2	T	C	0,015	0,003	1.450e-08	-0,016	0,008	0,053
rs1832007	10	AKR1C4	G	A	0,057	0,003	6.779e-60	0,003	0,010	0,741
rs2274224	10	PLCE1	G	C	0,024	0,003	1.017e-20	0,002	0,007	0,752
rs2801482	10	CAMK1D	G	A	0,050	0,008	1.130e-09	-0,017	0,022	0,451
rs293275	10	PRKG1	C	T	0,014	0,003	1.603e-08	0,006	0,007	0,403
rs3858325	10	GFRA1	T	C	0,019	0,003	1.713e-13	0,016	0,007	0,028
rs4418728	10	CYP26A1	G	T	0,024	0,003	5.991e-21	0,018	0,007	0,012
rs4917962	10	NOLC1	T	G	0,024	0,004	5.216e-10	0,005	0,010	0,611
rs7910087	10	C10orf11	T	C	0,017	0,003	6.229e-12	-0,011	0,007	0,118
rs7921105	10	BEND7	C	T	0,016	0,003	1.962e-10	0,005	0,007	0,483
rs9630085	10	FFAR4	G	A	0,022	0,003	9.144e-13	0,000	0,009	0,994
rs1039481	11	PTPRJ	G	A	0,042	0,003	4.113e-48	-0,005	0,008	0,544
rs10767874	11	DCDC1	A	G	0,015	0,003	3.973e-08	0,004	0,008	0,595
rs10892564	11	ARHGEF12	G	A	0,017	0,003	4.611e-11	0,002	0,007	0,783
rs10893499	11	ST3GAL4	G	A	0,022	0,004	3.842e-09	-0,003	0,010	0,776
rs11024614	11	HPS5	C	T	0,023	0,003	3.409e-18	-0,010	0,007	0,179
rs11029620	11	NUP98	C	T	0,022	0,003	4.620e-13	-0,005	0,009	0,545
rs11031058	11	RPL12P30	T	C	0,022	0,003	5.389e-11	-0,023	0,010	0,020
rs117104648	11	AP5B1	C	T	0,036	0,005	3.240e-12	0,013	0,017	0,417
rs117600498	11	ASCL2	C	T	0,038	0,007	9.548e-09	0,025	0,022	0,244
rs12790261	11	KDM2A	A	C	0,031	0,005	1.211e-11	-0,048	0,016	0,003
rs146345029	11	GIF	A	G	0,034	0,006	1.886e-08	0,024	0,019	0,206
rs174554	11	FADS1	A	G	0,022	0,003	7.202e-17	0,011	0,008	0,155
rs2512525	11	USP35	T	C	0,024	0,003	1.427e-12	-0,024	0,009	0,010
rs3213223	11	IGF2	A	G	0,076	0,003	9.35e-144	0,014	0,009	0,139
rs34452566	11	RP11-587D21.4	T	G	0,018	0,003	1.032e-08	0,010	0,010	0,320
rs35023999	11	ANKK1	C	A	0,015	0,003	1.160e-09	-0,017	0,007	0,020
rs4936759	11	C11orf63	C	T	0,016	0,003	1.949e-10	-0,004	0,007	0,547
rs4980661	11	AP000439.3	A	G	0,014	0,003	8.689e-09	-0,019	0,007	0,010
rs61867536	11	MOB2	T	C	0,018	0,003	1.132e-12	0,005	0,007	0,467
rs61904289	11	EED	T	C	0,016	0,003	1.392e-09	-0,007	0,008	0,373
rs625245	11	MRE11	G	T	0,016	0,003	4.364e-09	0,006	0,008	0,399
rs6485702	11	LRP4	T	C	0,017	0,003	1.415e-10	0,015	0,008	0,046
rs67257872	11	STK33	A	G	0,014	0,003	2.315e-08	-0,002	0,007	0,745
rs7115466	11	H19	A	G	0,015	0,003	2.286e-08	-0,001	0,009	0,948
rs72858776	11	RP11-396O20.2	G	T	0,030	0,005	1.479e-10	-0,014	0,013	0,288
rs7947951	11	ARNTL	G	A	0,020	0,003	1.025e-13	0,015	0,008	0,052
rs10745954	12	RP11-328J6.1	G	A	0,015	0,003	1.690e-09	-0,004	0,007	0,627
rs10777540	12	CRADD	G	T	0,018	0,003	2.236e-12	-0,016	0,007	0,032
rs10841649	12	SLCO1B3	C	T	0,021	0,004	9.254e-09	0,023	0,011	0,030
rs10860237	12	RP11-1016B18.1	A	G	0,030	0,003	2.391e-28	-0,007	0,008	0,395
rs11064536	12	WNK1	T	C	0,020	0,003	2.941e-09	-0,019	0,010	0,054
rs11111274	12	IGF1	G	A	0,080	0,003	7.59e-175	0,004	0,008	0,626

rs11175935	12	LRRK2	G	T	0,020	0,003	5.189e-10	-0,012	0,009	0,190
rs117564283	12	ACVRL1	T	C	0,029	0,005	5.046e-09	-0,016	0,015	0,311
rs12231073	12	RNA5SP358	T	G	0,017	0,003	5.421e-12	-0,017	0,007	0,019
rs12425869	12	SOCS2	A	G	0,018	0,003	3.957e-09	-0,032	0,009	0,000
rs1351394	12	HMGA2	C	T	0,024	0,003	6.924e-21	-0,017	0,007	0,018
rs1800574	12	HNF1A	T	C	0,145	0,007	3.641e-84	0,012	0,024	0,617
rs2230281	12	GALNT4	A	G	0,016	0,003	4.010e-09	0,019	0,008	0,017
rs2460488	12	RP11-110L15.1	G	A	0,026	0,003	1.096e-14	0,007	0,010	0,451
rs247917	12	ARID2	T	C	0,015	0,003	1.322e-09	0,024	0,007	0,001
rs2657879	12	GLS2	A	G	0,020	0,003	9.621e-10	-0,016	0,009	0,099
rs2856321	12	ETV6	G	A	0,026	0,003	1.583e-23	0,003	0,008	0,666
rs3759302	12	KIAA1551	A	T	0,021	0,003	6.949e-11	0,002	0,009	0,789
rs4547160	12	AVPR1A	T	G	0,018	0,003	3.168e-11	0,005	0,007	0,530
rs7314285	12	CUX2	G	T	0,052	0,005	2.043e-25	0,028	0,014	0,041
rs75938105	12	RP11-110L15.2	T	C	0,047	0,007	7.468e-12	0,028	0,023	0,223
rs773116	12	ERBB3	G	A	0,016	0,003	1.265e-10	-0,007	0,007	0,316
rs78607331	12	R3HDM2	C	T	0,037	0,006	6.059e-10	-0,005	0,020	0,801
rs9738365	12	RP11-428G5.4	A	C	0,058	0,003	1.905e-92	0,002	0,008	0,780
rs1170158	13	DGKH	T	G	0,021	0,003	1.041e-10	-0,004	0,009	0,673
rs118081390	13	FNDC3A	G	A	0,028	0,005	1.353e-08	0,011	0,015	0,479
rs1535793	13	LRCH1	A	G	0,024	0,003	7.422e-17	0,036	0,008	0,000
rs6602909	13	GAS6	C	T	0,020	0,003	4.354e-14	-0,006	0,008	0,431
rs71432868	13	SNORA25	C	T	0,028	0,005	3.105e-08	0,013	0,018	0,483
rs7323205	13	LINC00676	C	T	0,015	0,003	9.354e-09	0,002	0,007	0,819
rs9532512	13	LINC00598	A	G	0,043	0,003	8.024e-40	0,005	0,009	0,564
rs9573360	13	LINC00402	A	C	0,014	0,003	1.597e-08	-0,003	0,007	0,672
rs9583151	13	AL354741.1	C	T	0,014	0,003	2.708e-08	0,017	0,007	0,023
rs10136874	14	DLK1	G	T	0,023	0,003	2.350e-20	-0,007	0,008	0,377
rs10145154	14	NRXN3	T	C	0,018	0,003	9.924e-10	0,035	0,009	0,000
rs1061638	14	AHSA1	G	A	0,018	0,003	7.484e-11	0,014	0,008	0,068
rs1115897	14	UNC79	A	C	0,021	0,003	1.540e-14	0,010	0,008	0,206
rs13379043	14	ELMSAN1	T	C	0,025	0,003	5.776e-18	-0,003	0,008	0,672
rs168961	14	ZFP36L1	G	A	0,018	0,003	2.889e-13	0,016	0,007	0,025
rs17106640	14	ACTN1	G	A	0,017	0,003	8.431e-11	-0,014	0,008	0,072
rs175043	14	EIF2B2	G	A	0,018	0,003	1.834e-12	-0,005	0,007	0,536
rs28396553	14	AL162511.1	T	C	0,015	0,003	2.962e-09	0,000	0,007	0,976
rs28929474	14	SERPINA1	C	T	0,063	0,009	1.294e-12	-0,001	0,031	0,973
rs33912345	14	SIX6	C	A	0,023	0,003	2.363e-19	-0,006	0,007	0,435
rs36215895	14	SYNE2	C	T	0,082	0,013	2.121e-10	0,067	0,053	0,206
rs78598185	14	SLC24A4	G	A	0,029	0,004	7.090e-11	0,022	0,013	0,088
rs79936318	14	SYNE2	A	G	0,017	0,003	3.875e-08	-0,009	0,010	0,402
rs8017377	14	NYNRIN	G	A	0,017	0,003	3.353e-11	0,001	0,007	0,913
rs11856160	15	CHD2	A	G	0,021	0,003	2.843e-09	0,017	0,010	0,101
rs12442867	15	RP11-299H22.1	A	C	0,017	0,003	9.329e-11	0,006	0,007	0,427
rs12593755	15	RP11-97O12.3	T	G	0,016	0,003	1.846e-09	0,005	0,007	0,536

rs12912439	15	LINC01197	T	C	0,022	0,003	1.246e-15	0,007	0,008	0,359
rs142354201	15	PGPEP1L	G	A	0,034	0,006	3.677e-09	0,004	0,020	0,843
rs17747633	15	KNL1	G	A	0,015	0,003	3.080e-09	0,002	0,008	0,764
rs2004839	15	RP11-299H22.3	G	A	0,021	0,003	4.336e-10	0,003	0,010	0,729
rs2311313	15	RP11-35O15.1	G	T	0,019	0,003	1.691e-08	-0,031	0,010	0,002
rs2930313	15	CCDC33	G	A	0,028	0,005	4.141e-09	0,010	0,012	0,394
rs4545755	15	CYP19A1	G	A	0,016	0,003	3.865e-10	-0,008	0,007	0,246
rs55707100	15	MAP1A	C	T	0,151	0,008	1.406e-76	0,038	0,025	0,119
rs5742915	15	PML	C	T	0,025	0,003	1.723e-22	0,014	0,008	0,064
rs79076440	15	USP3	A	G	0,019	0,003	1.164e-08	-0,056	0,010	0,000
rs8033075	15	PIAS1	A	G	0,045	0,005	1.061e-17	0,002	0,017	0,914
rs11077337	16	ZNF597	T	G	0,015	0,003	1.454e-09	-0,022	0,007	0,002
rs11149612	16	RP11-505K9.4	C	T	0,027	0,003	1.131e-26	-0,020	0,008	0,007
rs111792934	16	HAS3	C	T	0,022	0,003	1.430e-10	0,006	0,010	0,514
rs116971887	16	SALL1	G	T	0,036	0,006	4.895e-09	-0,037	0,019	0,054
rs12597502	16	CHD9	G	A	0,015	0,003	4.528e-08	-0,002	0,008	0,773
rs12927172	16	IL4R	A	G	0,015	0,003	3.066e-09	0,008	0,007	0,283
rs12935091	16	ZNF19	G	A	0,035	0,006	2.235e-09	-0,006	0,019	0,766
rs12935465	16	XYLT1	T	C	0,016	0,003	5.769e-11	0,004	0,007	0,622
rs143076454	16	LMF1	G	A	0,072	0,009	1.245e-14	0,016	0,038	0,672
rs147491123	16	LINC01572	C	T	0,036	0,007	3.286e-08	0,029	0,022	0,182
rs1532824	16	ATF7IP2	A	C	0,017	0,003	4.521e-09	-0,015	0,008	0,071
rs1548917	16	RP11-461O7.1	T	C	0,015	0,003	2.361e-09	0,004	0,007	0,575
rs1657125	16	MEIOB	T	G	0,032	0,004	2.007e-18	0,008	0,010	0,406
rs17299478	16	NOB1	C	T	0,032	0,003	2.497e-21	-0,004	0,010	0,702
rs2023762	16	SYT17	T	C	0,015	0,003	5.991e-09	0,002	0,008	0,809
rs4786350	16	IFT140	C	G	0,036	0,006	6.048e-09	-0,012	0,019	0,542
rs4788220	16	FAM57B	G	A	0,017	0,003	5.263e-12	0,006	0,007	0,420
rs4985062	16	USP7	T	C	0,015	0,003	2.040e-09	0,010	0,007	0,173
rs4988483	16	SSTR5	C	A	0,172	0,006	1.87e-203	-0,067	0,021	0,001
rs61731445	16		C	T	0,028	0,005	1.958e-08	-0,007	0,016	0,666
rs7204824	16	LMF1	C	T	0,024	0,003	1.168e-16	0,008	0,008	0,306
rs72761177	16	NUBP2	A	G	0,077	0,004	7.205e-70	0,039	0,013	0,002
rs74774288	16	RP11-420N3.3	G	T	0,027	0,003	2.955e-17	0,006	0,010	0,521
rs7498665	16	SH2B1	G	A	0,019	0,003	7.173e-14	0,009	0,007	0,223
rs750952	16	ZNF646	C	T	0,032	0,003	1.105e-34	-0,011	0,008	0,158
rs753108	16	CMIP	A	G	0,025	0,003	1.606e-17	0,010	0,008	0,231
rs80253441	16	IGFALS	T	C	0,131	0,011	8.227e-36	-0,014	0,034	0,677
rs8054054	16	CCDC154	G	A	0,015	0,003	1.026e-09	-0,009	0,008	0,219
rs8054322	16	GSE1	A	G	0,015	0,003	4.723e-09	-0,003	0,008	0,714
rs8059803	16	CMIP	A	G	0,031	0,003	4.415e-29	0,011	0,008	0,172
rs8182173	16	CORO7-PAM16	T	C	0,018	0,003	1.610e-09	0,001	0,009	0,941
rs142377191	17	DCAF7	A	G	0,125	0,009	2.048e-46	0,019	0,033	0,560
rs1801689	17	APOH	C	A	0,089	0,007	1.248e-33	0,086	0,023	0,000
rs199525	17	WNT3	G	T	0,020	0,003	5.613e-11	0,040	0,009	0,000

rs2309401	17	NLRP1	T	G	0,015	0,003	6.785e-09	0,003	0,008	0,740
rs35819807	17	KCNH6	T	C	0,017	0,003	2.387e-09	0,019	0,009	0,022
rs3760237	17	SCN4A	C	T	0,024	0,003	1.441e-21	0,012	0,007	0,106
rs4075483	17	BAIAP2	C	T	0,017	0,003	1.312e-10	0,010	0,008	0,195
rs4789227	17	UNK	T	C	0,015	0,003	4.335e-09	0,005	0,008	0,514
rs56030650	17	GSDMA	C	A	0,022	0,003	2.417e-18	-0,014	0,007	0,058
rs6416868	17	TTC19	A	G	0,019	0,003	3.115e-14	-0,012	0,007	0,104
rs6501601	17	POLR3KP2	G	A	0,015	0,003	4.071e-09	-0,002	0,008	0,849
rs668799	17	COASY	C	T	0,018	0,003	4.019e-10	0,017	0,008	0,030
rs7502910	17	WDR81	A	G	0,016	0,003	1.528e-10	0,017	0,007	0,021
rs76708468	17	1ERN	C	T	0,087	0,006	3.084e-41	-0,011	0,025	0,648
rs77542162	17	ABCA6	G	A	0,054	0,009	2.412e-10	-0,018	0,030	0,557
rs8075153	17	RAI1	C	T	0,021	0,003	1.831e-17	-0,006	0,007	0,444
rs8079923	17	AKAP10	C	T	0,016	0,003	3.664e-08	-0,006	0,009	0,494
rs9892862	17	Y_RNA	G	A	0,022	0,003	9.705e-14	-0,011	0,008	0,213
rs11152071	18	RP11-1151B14.2	C	T	0,020	0,003	8.957e-12	-0,009	0,008	0,278
rs12454712	18	BCL2	T	C	0,018	0,003	4.051e-12	-0,006	0,008	0,410
rs190102446	18	RP11-27G24.1	C	T	0,041	0,007	2.452e-09	0,000	0,020	0,999
rs57551555	18	RP11-176N18.2	T	G	0,019	0,003	5.817e-13	0,008	0,008	0,288
rs585187	18	MRPS5P4	T	G	0,015	0,003	3.712e-09	-0,010	0,007	0,176
rs8084351	18	DCC	G	A	0,015	0,003	8.537e-10	0,003	0,007	0,731
rs8095538	18	-	G	T	0,020	0,003	8.447e-14	0,004	0,008	0,628
rs8097893	18	GALR1	A	G	0,058	0,006	1.723e-20	0,034	0,017	0,045
rs11671304	19	ZC3H4	C	T	0,018	0,003	6.884e-11	-0,016	0,008	0,060
rs12975366	19	LILRB5	T	C	0,020	0,003	2.253e-15	-0,009	0,008	0,269
rs2287922	19	RASIP1	G	A	0,030	0,003	1.833e-33	0,001	0,008	0,948
rs296361	19	SULT2A1	A	G	0,025	0,003	3.421e-13	0,004	0,010	0,698
rs34536443	19	TYK2	G	C	0,045	0,006	9.964e-14	-0,016	0,020	0,426
rs3760954	19	MIR7-3HG	T	C	0,024	0,004	2.909e-10	0,006	0,011	0,581
rs58560372	19	SPINT2	C	T	0,020	0,003	1.331e-08	-0,013	0,010	0,210
rs58658292	19	ZNF536	G	A	0,030	0,006	4.618e-08	-0,010	0,017	0,539
rs62102136	19	LSM14A	C	T	0,016	0,003	6.527e-09	-0,014	0,008	0,093
rs6510033	19	AC005597.1	A	G	0,020	0,003	1.147e-12	-0,002	0,008	0,805
rs6510177	19	ZNF536	T	C	0,023	0,003	1.598e-12	0,005	0,010	0,641
rs6510832	19	KDM4B	G	T	0,033	0,005	2.638e-10	0,063	0,016	0,000
rs67868323	19	ZBTB7A	T	G	0,016	0,003	2.205e-08	0,002	0,009	0,849
rs7254601	19	COX6B1	G	A	0,016	0,003	2.551e-08	-0,005	0,009	0,554
rs7256521	19	ZNF845	G	A	0,015	0,003	9.761e-10	-0,016	0,007	0,036
rs8105174	19	DNMT1	C	T	0,050	0,003	7.971e-54	0,034	0,010	0,001
rs8112883	19	INSR	G	T	0,017	0,003	1.166e-09	-0,008	0,008	0,334
rs8113618	19	QTRT1	T	C	0,031	0,003	1.185e-33	-0,007	0,007	0,326
rs16995311	20	PTPN1	A	C	0,040	0,005	5.559e-18	-0,019	0,015	0,185
rs17265513	20	ZHX3	T	C	0,022	0,003	3.454e-12	0,002	0,010	0,868
rs2207132	20	LINC01728	G	A	0,048	0,007	1.064e-11	0,027	0,023	0,246
rs2424396	20	LINC01726	G	A	0,033	0,004	2.245e-14	0,029	0,013	0,022

rs2738787	20	TNFRSF6B	A	G	0,037	0,005	1.251e-15	0,003	0,014	0,820
rs4809401	20	NPBWR2	T	C	0,023	0,004	6.488e-10	-0,010	0,011	0,360
rs6037508	20	SLC4A11	G	T	0,017	0,003	8.740e-09	0,014	0,009	0,111
rs6046825	20	RALGAPA2	A	C	0,024	0,003	2.108e-17	0,001	0,008	0,904
rs6088579	20	NCOA6	G	A	0,027	0,003	8.509e-17	-0,007	0,010	0,482
rs7267595	20	JAG1	A	C	0,015	0,003	9.437e-10	-0,002	0,007	0,838
rs7508949	20	CRNKL1	C	G	0,025	0,003	1.490e-22	0,019	0,008	0,013
rs75989562	20	APMAP	A	G	0,030	0,005	8.093e-09	0,060	0,018	0,001
rs9978775	21	BRWD1-AS1	G	A	0,019	0,003	2.351e-13	-0,024	0,007	0,001
rs12106594	22	EIF4ENIF1	T	C	0,036	0,006	1.588e-09	-0,011	0,017	0,520
rs2412973	22	HORMAD2	A	C	0,014	0,003	3.334e-08	0,002	0,007	0,759
rs4823324	22	ATXN10	T	C	0,016	0,003	1.938e-10	-0,009	0,007	0,234
rs5755948	22	RBFOX2	A	G	0,028	0,004	4.375e-14	0,009	0,011	0,414
rs6519133	22	JOSD1	T	C	0,029	0,003	7.731e-30	-0,013	0,008	0,085
rs8138950	22	ZNRF3	C	T	0,015	0,003	1.323e-09	-0,011	0,007	0,141
rs9611565	22	TEF	T	C	0,029	0,003	3.825e-23	-0,011	0,009	0,185

Chr, chromosome; EA, effect allele; OA, other allele; SE, standard error.

ESM Table 5. Summary statistics of the single-nucleotide polymorphisms associated with insulin-like growth factor-1 and their associations with ischemic stroke

					IGF-1			Ischemic stroke		
SNP	Chr	Nearby gene	EA	OA	Beta	SE	*p*	Beta	SE	*p*
rs10159299	1	PLXNA2	C	T	0,018	0,003	5.112e-12	0,004	0,008	0,642
rs1046011	1	LEPROT	T	C	0,021	0,003	3.303e-14	0,000	0,010	0,980
rs10779509	1	RP1-272L16.1	C	T	0,014	0,003	2.109e-08	-0,005	0,009	0,596
rs11165778	1	HFM1	A	G	0,016	0,003	1.492e-08	0,012	0,011	0,265
rs112436634	1	PEX14	T	C	0,016	0,003	1.749e-09	0,001	0,009	0,894
rs1127313	1	ADAR	G	A	0,024	0,003	1.394e-21	-0,001	0,009	0,923
rs11577063	1	AXDND1	T	G	0,020	0,003	1.511e-11	-0,002	0,010	0,840
rs12141189	1	HLX	T	C	0,045	0,003	4.135e-53	0,004	0,010	0,723
rs1223763	1	RP11-53A1.3	T	G	0,024	0,003	8.461e-13	0,004	0,010	0,670
rs12723255	1	EIF4G3	C	T	0,017	0,003	3.229e-11	0,004	0,009	0,687
rs12749024	1	PAPPA2	T	C	0,075	0,004	7.72e-100	-0,024	0,014	0,098
rs140604451	1	GSTM2	G	A	0,054	0,008	3.524e-11	0,042	0,030	0,161
rs1430753	1	WLS	A	G	0,021	0,003	7.085e-11	-0,011	0,011	0,330
rs143885630	1	SMG7	G	A	0,030	0,004	1.358e-15	0,009	0,013	0,463
rs165316	1	RPL5P6	G	A	0,073	0,003	9.66e-119	-0,006	0,011	0,596
rs17037452	1	CLCN6	A	G	0,023	0,003	1.658e-11	0,021	0,011	0,060
rs17393144	1	MIR34AHG	A	G	0,016	0,003	9.319e-09	0,002	0,010	0,869
rs1825813	1	C1orf146	A	G	0,023	0,003	1.879e-13	-0,003	0,012	0,837
rs2075995	1	E2F2	C	A	0,014	0,003	9.790e-09	-0,001	0,009	0,951
rs2724373	1	C1orf132	C	T	0,019	0,003	1.436e-12	0,018	0,010	0,068
rs2802330	1	PDIK1L	G	A	0,031	0,003	4.632e-21	-0,037	0,011	0,001
rs2802951	1	RN7SL668P	A	G	0,016	0,003	4.970e-09	-0,002	0,011	0,820
rs2819336	1	PTPRF	T	C	0,027	0,003	1.550e-25	-0,012	0,009	0,191
rs3131646	1	MYCL	G	T	0,016	0,003	1.710e-08	-0,013	0,009	0,137
rs36086195	1	ARHGEF19-AS1	C	T	0,019	0,003	1.359e-13	-0,013	0,009	0,133
rs4306136	1	RP11-103C3.1	A	G	0,017	0,003	1.997e-11	0,001	0,009	0,884
rs569356	1	OPRD1	G	A	0,027	0,004	5.953e-14	0,017	0,015	0,256
rs599839	1	CELSR2	G	A	0,031	0,003	9.629e-26	-0,012	0,011	0,276
rs61780439	1	SLFNL1	G	A	0,021	0,003	5.323e-12	-0,008	0,012	0,522
rs6659176	1	NR0B2	C	G	0,042	0,005	1.354e-19	0,014	0,017	0,412
rs6701954	1	USP48	T	G	0,014	0,003	3.497e-08	0,008	0,009	0,382
rs684818	1	RP4-781K5.7	T	C	0,024	0,003	9.658e-21	-0,015	0,009	0,086
rs708108	1	WNT3A	T	C	0,015	0,003	5.872e-09	0,026	0,010	0,008
rs7517340	1	AKT3	C	T	0,035	0,003	2.123e-26	0,005	0,010	0,601
rs7528548	1	PYGO2	C	T	0,054	0,009	7.954e-10	-0,054	0,026	0,039
rs7539178	1	JAK1	C	A	0,026	0,004	1.365e-12	0,014	0,011	0,223
rs7545345	1	NUCKS1	C	T	0,026	0,004	1.032e-12	0,015	0,014	0,276
rs75681856	1	RABGAP1L	T	C	0,023	0,004	5.171e-09	-0,037	0,013	0,004
rs75907879	1	SPEN	T	C	0,024	0,004	1.425e-10	0,033	0,018	0,071
rs77369503	1	RGS4	G	A	0,045	0,007	1.397e-10	-0,033	0,032	0,289

rs903908	1	SKI	C	T	0,016	0,003	2.176e-10	-0,003	0,009	0,769
rs11677980	2	LBH	G	A	0,015	0,003	3.043e-08	0,018	0,011	0,101
rs11678946	2	EPHA4	A	C	0,014	0,003	3.143e-08	-0,009	0,009	0,310
rs12471768	2	SERTAD2	C	T	0,022	0,003	1.639e-15	0,005	0,010	0,595
rs1260326	2	GCKR	C	T	0,063	0,003	9.66e-133	-0,006	0,009	0,516
rs12710648	2	SMC6	A	G	0,017	0,003	5.703e-12	0,009	0,009	0,315
rs1465529	2	SP110	T	C	0,019	0,003	1.386e-12	0,008	0,010	0,402
rs17050272	2	AC073257.2	G	A	0,024	0,003	3.201e-20	0,010	0,009	0,269
rs17323117	2	NEU2	G	A	0,029	0,005	7.980e-10	0,007	0,018	0,687
rs17400325	2	PDE11A	C	T	0,054	0,006	9.393e-17	0,017	0,026	0,504
rs2674492	2	CYBRD1	A	G	0,014	0,003	4.835e-08	0,013	0,009	0,143
rs35135518	2	AC010145.4	T	C	0,029	0,004	1.521e-12	0,014	0,012	0,266
rs35641591	2	PCBP1-AS1	C	T	0,050	0,006	1.575e-14	-0,017	0,031	0,585
rs3791679	2	EFEMP1	G	A	0,018	0,003	4.338e-09	0,000	0,010	0,993
rs4402747	2	NEU2	A	G	0,016	0,003	2.960e-10	-0,019	0,008	0,021
rs58387407	2	CACNB4	G	A	0,018	0,003	3.502e-08	0,011	0,010	0,266
rs62136965	2	RNU6-566P	C	T	0,037	0,006	6.429e-10	0,012	0,025	0,640
rs62182127	2	VIL1	A	G	0,019	0,003	2.712e-14	-0,014	0,009	0,100
rs6435156	2	BMPR2	C	T	0,024	0,003	2.045e-17	-0,021	0,011	0,045
rs6437249	2	HDLBP	C	T	0,019	0,003	4.559e-12	-0,014	0,010	0,150
rs6544549	2	KCNG3	T	C	0,024	0,004	1.592e-10	0,002	0,013	0,863
rs6749680	2	ALMS1	A	G	0,015	0,003	9.868e-09	-0,002	0,009	0,825
rs6760135	2	ASXL2	T	C	0,050	0,003	1.194e-63	0,005	0,012	0,652
rs702878	2	AC074391.1	A	G	0,014	0,003	1.815e-08	-0,008	0,009	0,357
rs73954943	2	BCL2L11	A	G	0,031	0,005	1.063e-09	0,020	0,015	0,178
rs7574340	2	SLC8A1	T	C	0,017	0,003	2.971e-10	-0,016	0,010	0,113
rs7578633	2	PAX8	T	C	0,018	0,003	3.379e-12	-0,017	0,009	0,053
rs112893170	3	FEZF2	T	C	0,020	0,003	1.527e-10	0,026	0,012	0,030
rs11717397	3	UBE2E2	G	A	0,015	0,003	2.572e-09	-0,007	0,009	0,401
rs11928797	3	UBP1	A	C	0,030	0,004	2.428e-14	0,024	0,015	0,119
rs12491473	3	CCDC12	G	A	0,020	0,003	5.913e-15	-0,021	0,009	0,017
rs13069961	3	KALRN	G	A	0,018	0,003	3.680e-09	-0,011	0,011	0,306
rs13073970	3	EIF5A2	T	G	0,025	0,003	2.016e-15	0,000	0,011	0,968
rs1822825	3	PPARG	G	A	0,014	0,003	2.886e-08	0,000	0,008	0,988
rs2268829	3	DGKG	G	A	0,018	0,003	3.038e-09	0,007	0,010	0,484
rs2607748	3	CHCHD4	C	T	0,017	0,003	3.289e-11	0,009	0,009	0,279
rs3772102	3	ST3GAL6	G	T	0,020	0,003	5.954e-16	-0,020	0,009	0,019
rs4678497	3	SUSD5	C	T	0,017	0,003	1.959e-10	-0,013	0,009	0,119
rs504603	3	BZW1P1	C	T	0,029	0,005	2.402e-09	-0,002	0,016	0,925
rs55717031	3	MRPS22	G	T	0,032	0,003	2.474e-31	0,000	0,010	0,991
rs56062334	3	LINC02068	T	C	0,017	0,003	1.839e-11	0,004	0,009	0,660
rs62263345	3	BBX	A	G	0,028	0,004	1.640e-14	0,019	0,015	0,209
rs62280667	3	SENP7	C	T	0,028	0,003	2.477e-26	0,001	0,009	0,906
rs6440008	3	ZBTB38	T	C	0,035	0,003	1.113e-41	0,001	0,009	0,881
rs66707192	3	HRG	G	A	0,018	0,003	4.415e-10	-0,003	0,009	0,752

rs687339	3	KRT18P35	T	C	0,040	0,003	2.666e-40	-0,001	0,010	0,947
rs73238159	3	XRN1	C	T	0,025	0,004	1.059e-11	0,002	0,013	0,897
rs7625680	3	ATG7	A	G	0,015	0,003	7.693e-09	0,009	0,009	0,296
rs7628689	3	C3orf38	G	A	0,029	0,003	6.825e-17	-0,004	0,012	0,737
rs811332	3	MRAS	C	T	0,019	0,003	2.011e-09	-0,028	0,012	0,017
rs9819762	3	PIK3CA	T	C	0,019	0,003	1.212e-08	0,012	0,011	0,277
rs1055582	4	UBE2K	C	T	0,027	0,003	1.578e-27	-0,010	0,009	0,257
rs111443396	4	LINC01091	C	T	0,026	0,004	2.015e-10	0,027	0,014	0,044
rs1229984	4	ADH1B	T	C	0,104	0,009	1.219e-34	-0,043	0,018	0,016
rs13108218	4	HGFAC	G	A	0,017	0,003	5.920e-11	-0,016	0,009	0,070
rs17429745	4	RP11-556I14.1	G	T	0,026	0,003	2.045e-21	-0,003	0,009	0,751
rs2280099	4	TIGD2	G	A	0,025	0,003	1.923e-14	-0,004	0,012	0,766
rs35036084	4	RP11-145G20.1	T	C	0,017	0,003	8.218e-11	-0,011	0,009	0,218
rs3804173	4	PRDM5	G	A	0,020	0,003	4.484e-13	0,006	0,009	0,495
rs4394044	4	SORBS2	T	C	0,014	0,003	4.708e-08	-0,011	0,009	0,192
rs62302688	4	GABRA2	G	A	0,039	0,004	4.057e-20	-0,019	0,019	0,311
rs62334147	4	DDX60L	C	T	0,019	0,003	4.261e-09	-0,009	0,012	0,484
rs62342064	4	RP11-119H12.3	C	T	0,022	0,004	1.836e-09	0,002	0,017	0,888
rs6532798	4	ADH4	T	C	0,037	0,003	1.364e-41	0,017	0,010	0,084
rs6827641	4	HHIP	T	C	0,014	0,003	2.623e-08	0,011	0,008	0,174
rs6853741	4	ARHGAP10	A	G	0,024	0,003	1.676e-16	-0,021	0,010	0,026
rs7667562	4	LARP1B	C	A	0,016	0,003	3.770e-09	0,005	0,009	0,614
rs11242236	5	C5orf66	G	A	0,025	0,003	1.957e-22	0,014	0,009	0,106
rs11954036	5	PDE4D	T	C	0,037	0,003	1.936e-44	0,010	0,009	0,290
rs12108803	5	TBCA	G	T	0,033	0,006	1.015e-08	-0,001	0,019	0,970
rs12520263	5	RP11-357F12.1	T	G	0,017	0,003	2.579e-09	0,012	0,009	0,180
rs13168379	5	CPEB4	A	G	0,031	0,005	6.148e-10	0,023	0,013	0,075
rs13178887	5	MEF2C-AS1	T	C	0,023	0,003	2.712e-19	0,003	0,009	0,712
rs1498603	5	PDE4D	T	G	0,031	0,005	1.170e-09	-0,004	0,017	0,820
rs2042253	5	MIR5197	T	C	0,023	0,003	1.017e-14	-0,005	0,009	0,607
rs2227819	5	F2R	T	C	0,022	0,004	4.322e-08	0,035	0,016	0,031
rs2366398	5	CTD-2151A2.3	T	G	0,018	0,003	1.613e-09	-0,001	0,010	0,952
rs258775	5	ARHGAP26	A	C	0,025	0,003	3.394e-14	0,007	0,011	0,500
rs26822	5	PPIP5K2	G	A	0,017	0,003	9.845e-11	0,004	0,009	0,677
rs28650790	5	C5orf67	T	C	0,018	0,003	4.108e-08	0,022	0,012	0,077
rs329122	5	JADE2	A	G	0,018	0,003	1.312e-12	0,003	0,008	0,767
rs35668185	5	SLIT3	T	C	0,056	0,003	3.402e-73	0,020	0,010	0,052
rs3734166	5	CDC25C	A	G	0,028	0,003	8.063e-22	0,001	0,009	0,874
rs6180	5	GHR	A	C	0,035	0,003	6.019e-43	-0,010	0,008	0,255
rs6895953	5	RP11-357F12.1	G	A	0,024	0,003	5.167e-21	0,000	0,008	0,982
rs72758321	5	PLCXD3	G	A	0,047	0,006	3.926e-15	0,029	0,029	0,326
rs73271090	5	CTB-1I21.1	G	A	0,044	0,003	7.844e-39	0,025	0,012	0,042
rs7719168	5	15ARL	C	A	0,030	0,004	2.429e-14	0,004	0,015	0,780
rs80170948	5	SREK1IP1	G	T	0,039	0,006	2.221e-09	-0,073	0,028	0,009
rs840809	5	CTD-2232E5.2	A	C	0,016	0,003	1.183e-08	-0,003	0,010	0,775

rs9292578	5	PRLR	C	A	0,040	0,006	3.195e-10	-0,017	0,020	0,395
rs113127944	6	CENPW	A	G	0,051	0,008	6.115e-11	0,029	0,035	0,412
rs1150781	6	C6orf1	G	C	0,026	0,004	2.337e-09	-0,032	0,014	0,021
rs1165196	6	SLC17A1	G	A	0,029	0,003	6.414e-30	-0,011	0,009	0,234
rs12110787	6	MAP3K4	A	C	0,022	0,004	1.857e-08	0,013	0,012	0,258
rs12194618	6	ZFAND3	A	G	0,017	0,003	2.686e-11	0,016	0,010	0,118
rs2296198	6	RNF144B	C	T	0,016	0,003	1.937e-08	-0,001	0,010	0,946
rs2397112	6	RP11-228O6.2	A	G	0,019	0,003	3.313e-13	-0,003	0,009	0,722
rs3008051	6	PDE10A	C	T	0,014	0,003	1.946e-08	0,007	0,009	0,457
rs3127579	6	SLC22A2	A	G	0,033	0,004	1.230e-19	0,014	0,013	0,295
rs3890746	6	L3MBTL3	C	T	0,020	0,003	1.060e-15	-0,010	0,009	0,238
rs41285260	6	CENPW	T	G	0,039	0,004	5.123e-18	0,004	0,018	0,847
rs4709995	6	SDIM1	T	C	0,042	0,003	7.369e-60	-0,007	0,009	0,421
rs584955	6	TMEM14C	A	G	0,036	0,006	2.579e-09	-0,002	0,020	0,921
rs670049	6	Y_RNA	A	C	0,019	0,003	6.417e-13	0,019	0,010	0,055
rs6916994	6	GJB7	C	T	0,029	0,003	1.519e-31	0,016	0,008	0,057
rs6924225	6	RUNX2	G	A	0,019	0,003	1.442e-08	-0,014	0,013	0,266
rs73382439	6	E2F3	C	T	0,019	0,003	1.800e-08	0,005	0,011	0,636
rs7740433	6	CNPY3	A	G	0,017	0,003	1.504e-08	-0,003	0,011	0,761
rs7758644	6	RP1-155D22.1	A	C	0,019	0,003	1.514e-08	-0,006	0,012	0,644
rs7774230	6	ESR1	T	C	0,026	0,003	5.464e-25	-0,002	0,008	0,776
rs790513	6	OPRM1	C	A	0,025	0,003	1.575e-18	-0,011	0,011	0,320
rs9321106	6	PTPRK	A	G	0,018	0,003	2.408e-08	0,005	0,012	0,694
rs9322822	6	LIN28B-AS1	C	T	0,015	0,003	1.349e-08	0,010	0,009	0,284
rs9364815	6	PDE10A	A	G	0,015	0,003	2.829e-08	0,005	0,009	0,582
rs9398171	6	FOXO3	T	C	0,050	0,003	9.510e-74	-0,012	0,009	0,186
rs9398891	6	LAMA2	T	C	0,017	0,003	1.108e-10	-0,004	0,010	0,716
rs998584	6	VEGFA	A	C	0,020	0,003	1.211e-15	0,019	0,009	0,037
rs10246481	7	AC073133.2	G	A	0,015	0,003	1.499e-08	0,000	0,009	0,979
rs10252510	7	GHRHR	G	A	0,020	0,003	8.179e-14	-0,012	0,010	0,240
rs1050327	7	ZMIZ2	A	G	0,017	0,003	2.907e-11	0,022	0,009	0,011
rs114949263	7	TMEM176B	T	C	0,027	0,004	1.283e-11	0,005	0,016	0,743
rs11556924	7	ZC3HC1	C	T	0,016	0,003	9.798e-10	0,006	0,011	0,575
rs1182174	7	GNA12	A	G	0,021	0,003	4.808e-14	0,015	0,009	0,113
rs12666306	7	RP11-222O23.1	G	A	0,017	0,003	1.702e-11	-0,005	0,009	0,549
rs12699547	7	MAD1L1	C	T	0,021	0,003	2.438e-16	-0,017	0,009	0,052
rs17145738	7	TBL2	T	C	0,034	0,004	8.750e-19	-0,016	0,014	0,236
rs1986692	7	EXOC4	G	A	0,015	0,003	8.640e-09	-0,004	0,009	0,616
rs2048672	7	LINC-PINT	C	A	0,018	0,003	6.405e-11	0,007	0,010	0,510
rs2250243	7	ZNF316	C	T	0,024	0,003	1.694e-15	-0,020	0,010	0,041
rs2270628	7	IGFBP3	T	C	0,033	0,003	3.737e-25	-0,001	0,010	0,957
rs273956	7	CREB3L2	A	G	0,021	0,003	5.369e-16	-0,021	0,009	0,018
rs2896395	7	SND1	C	T	0,015	0,003	2.446e-08	0,008	0,009	0,384
rs34312198	7	ZNF3	A	C	0,024	0,004	1.345e-09	0,002	0,016	0,908
rs35862187	7	AUTS2	A	G	0,031	0,006	3.484e-08	0,019	0,022	0,389

rs411717	7	COL1A2	T	C	0,015	0,003	2.144e-09	-0,011	0,009	0,188
rs4719393	7	DGKB	T	G	0,027	0,003	1.290e-22	-0,013	0,009	0,158
rs74657816	7	HMGN1P19	T	G	0,047	0,005	3.006e-18	-0,028	0,018	0,125
rs7783012	7	FOXP2	G	A	0,016	0,003	1.561e-10	-0,010	0,009	0,247
rs7802508	7	ZFAND2A	A	G	0,021	0,003	9.477e-17	0,000	0,009	0,976
rs79881512	7	AC073325.1	C	T	0,059	0,011	4.185e-08	0,027	0,033	0,405
rs870796	7	ELK1P1	G	A	0,017	0,003	3.274e-11	0,010	0,009	0,241
rs11782452	8	BNIP3L	G	A	0,015	0,003	1.258e-08	0,001	0,009	0,884
rs12549853	8	PLEC	A	G	0,016	0,003	1.654e-09	0,006	0,009	0,540
rs1431015	8	RNU2-54P	C	T	0,020	0,003	2.655e-14	-0,017	0,009	0,049
rs1495741	8	NAT2	G	A	0,026	0,003	5.034e-18	0,025	0,010	0,012
rs1786342	8	SNX31	T	C	0,017	0,003	1.241e-11	0,017	0,009	0,052
rs2737205	8	TRPS1	T	C	0,023	0,003	4.711e-20	0,003	0,009	0,773
rs2978062	8	ST3GAL1	G	T	0,019	0,003	3.225e-08	0,021	0,013	0,104
rs445036	8	ZBTB10	T	C	0,019	0,003	5.794e-12	-0,003	0,010	0,775
rs56352849	8	KCNB2	A	G	0,016	0,003	1.497e-08	-0,011	0,010	0,231
rs60862542	8	EIF3E	G	A	0,017	0,003	1.648e-08	0,006	0,010	0,578
rs6473015	8	AC105242.1	C	A	0,019	0,003	2.994e-12	-0,004	0,010	0,687
rs716100	8	ZFAT	A	G	0,019	0,003	7.196e-13	0,010	0,009	0,268
rs76393968	8	MSR1	G	A	0,060	0,010	2.566e-09	-0,022	0,034	0,518
rs9657541	8	CTD-2135J3.4	C	T	0,020	0,003	3.846e-10	0,000	0,012	0,976
rs1055710	9	FAM120AOS	G	A	0,018	0,003	7.149e-12	-0,011	0,009	0,194
rs10757291	9	CDKN2B-AS1	G	A	0,019	0,003	1.487e-14	-0,003	0,009	0,750
rs10811787	9	RP11-370B11.4	C	T	0,015	0,003	7.833e-09	-0,001	0,008	0,890
rs10869022	9	TRPM3	C	T	0,021	0,003	1.633e-11	0,016	0,010	0,113
rs10908903	9	GADD45G	T	G	0,015	0,003	1.288e-09	0,010	0,008	0,226
rs11557154	9	DCAF12	T	C	0,024	0,004	3.100e-10	0,001	0,012	0,960
rs13301073	9	MAPKAP1	G	A	0,022	0,003	4.056e-17	0,012	0,009	0,195
rs2378662	9	RP11-158D2.2	G	A	0,017	0,003	4.195e-11	-0,009	0,009	0,314
rs28831479	9	PTCH1	C	A	0,022	0,003	1.196e-14	0,015	0,010	0,119
rs7034716	9	TGFBR1	C	T	0,015	0,003	2.595e-08	-0,028	0,010	0,007
rs7041137	9	RAD23B	T	C	0,017	0,003	7.298e-10	0,003	0,009	0,788
rs7872812	9	ASTN2	T	C	0,026	0,004	3.162e-13	0,009	0,013	0,472
rs10047326	10	PIP4K2A	A	C	0,017	0,003	1.263e-10	0,001	0,009	0,915
rs10509746	10	Y_RNA	C	T	0,027	0,003	1.943e-26	0,006	0,009	0,511
rs10821713	10	ANK3	T	C	0,017	0,003	5.068e-11	-0,004	0,008	0,632
rs11012712	10		C	T	0,022	0,003	3.752e-12	0,013	0,012	0,268
rs12244851	10	TCF7L2	T	C	0,015	0,003	1.450e-08	0,024	0,010	0,020
rs1832007	10	AKR1C4	G	A	0,057	0,003	6.779e-60	0,019	0,013	0,143
rs2274224	10	PLCE1	G	C	0,024	0,003	1.017e-20	0,024	0,009	0,005
rs2801482	10	CAMK1D	G	A	0,050	0,008	1.130e-09	-0,008	0,024	0,728
rs293275	10	PRKG1	C	T	0,014	0,003	1.603e-08	0,002	0,009	0,813
rs3858325	10	GFRA1	T	C	0,019	0,003	1.713e-13	0,004	0,008	0,638
rs4418728	10	CYP26A1	G	T	0,024	0,003	5.991e-21	0,035	0,008	0,000
rs4917962	10	NOLC1	T	G	0,024	0,004	5.216e-10	0,010	0,012	0,387

rs7910087	10	C10orf11	T	C	0,017	0,003	6.229e-12	-0,004	0,009	0,616
rs7921105	10	BEND7	C	T	0,016	0,003	1.962e-10	0,021	0,009	0,016
rs9630085	10	FFAR4	G	A	0,022	0,003	9.144e-13	0,016	0,010	0,106
rs1039481	11	PTPRJ	G	A	0,042	0,003	4.113e-48	-0,001	0,009	0,898
rs10767874	11	DCDC1	A	G	0,015	0,003	3.973e-08	0,005	0,009	0,618
rs10892564	11	ARHGEF12	G	A	0,017	0,003	4.611e-11	0,007	0,009	0,444
rs10893499	11	ST3GAL4	G	A	0,022	0,004	3.842e-09	0,015	0,012	0,193
rs11024614	11	HPS5	C	T	0,023	0,003	3.409e-18	0,016	0,009	0,060
rs11029620	11	NUP98	C	T	0,022	0,003	4.620e-13	0,002	0,011	0,855
rs11031058	11	RPL12P30	T	C	0,022	0,003	5.389e-11	-0,007	0,012	0,555
rs117104648	11	AP5B1	C	T	0,036	0,005	3.240e-12	0,001	0,022	0,963
rs117600498	11	ASCL2	C	T	0,038	0,007	9.548e-09	0,017	0,027	0,537
rs12790261	11	KDM2A	A	C	0,031	0,005	1.211e-11	-0,014	0,024	0,565
rs146345029	11	GIF	A	G	0,034	0,006	1.886e-08	0,000	0,024	0,987
rs174554	11	FADS1	A	G	0,022	0,003	7.202e-17	0,030	0,009	0,001
rs2512525	11	USP35	T	C	0,024	0,003	1.427e-12	-0,015	0,010	0,158
rs3213223	11	IGF2	A	G	0,076	0,003	9.35e-144	0,011	0,012	0,351
rs34452566	11	RP11-587D21.4	T	G	0,018	0,003	1.032e-08	-0,006	0,013	0,620
rs35023999	11	ANKK1	C	A	0,015	0,003	1.160e-09	-0,001	0,009	0,907
rs4936759	11	C11orf63	C	T	0,016	0,003	1.949e-10	-0,014	0,009	0,114
rs4980661	11	AP000439.3	A	G	0,014	0,003	8.689e-09	0,012	0,009	0,181
rs61867536	11	MOB2	T	C	0,018	0,003	1.132e-12	0,006	0,009	0,555
rs61904289	11	EED	T	C	0,016	0,003	1.392e-09	0,007	0,010	0,508
rs625245	11	MRE11	G	T	0,016	0,003	4.364e-09	0,030	0,009	0,001
rs6485702	11	LRP4	T	C	0,017	0,003	1.415e-10	0,016	0,009	0,084
rs67257872	11	STK33	A	G	0,014	0,003	2.315e-08	-0,007	0,009	0,426
rs7115466	11	H19	A	G	0,015	0,003	2.286e-08	0,017	0,016	0,287
rs72858776	11	RP11-396O20.2	G	T	0,030	0,005	1.479e-10	0,010	0,014	0,492
rs7947951	11	ARNTL	G	A	0,020	0,003	1.025e-13	0,010	0,009	0,259
rs10745954	12	RP11-328J6.1	G	A	0,015	0,003	1.690e-09	-0,014	0,009	0,108
rs10777540	12	CRADD	G	T	0,018	0,003	2.236e-12	-0,013	0,009	0,133
rs10841649	12	SLCO1B3	C	T	0,021	0,004	9.254e-09	0,027	0,013	0,031
rs10860237	12	RP11-1016B18.1	A	G	0,030	0,003	2.391e-28	0,000	0,009	0,971
rs11064536	12	WNK1	T	C	0,020	0,003	2.941e-09	0,014	0,013	0,286
rs11111274	12	IGF1	G	A	0,080	0,003	7.59e-175	-0,008	0,009	0,410
rs11175935	12	LRRK2	G	T	0,020	0,003	5.189e-10	0,003	0,011	0,764
rs117564283	12	ACVRL1	T	C	0,029	0,005	5.046e-09	0,032	0,019	0,102
rs12231073	12	RNA5SP358	T	G	0,017	0,003	5.421e-12	-0,017	0,009	0,053
rs12425869	12	SOCS2	A	G	0,018	0,003	3.957e-09	-0,003	0,010	0,756
rs1351394	12	HMGA2	C	T	0,024	0,003	6.924e-21	0,011	0,009	0,220
rs1800574	12	HNF1A	T	C	0,145	0,007	3.641e-84	0,038	0,032	0,231
rs2230281	12	GALNT4	A	G	0,016	0,003	4.010e-09	0,013	0,010	0,170
rs2460488	12	RP11-110L15.1	G	A	0,026	0,003	1.096e-14	-0,006	0,013	0,658
rs247917	12	ARID2	T	C	0,015	0,003	1.322e-09	0,019	0,008	0,026
rs2657879	12	GLS2	A	G	0,020	0,003	9.621e-10	0,004	0,012	0,770

rs2856321	12	ETV6	G	A	0,026	0,003	1.583e-23	-0,016	0,009	0,085
rs3759302	12	KIAA1551	A	T	0,021	0,003	6.949e-11	-0,003	0,011	0,754
rs4547160	12	AVPR1A	T	G	0,018	0,003	3.168e-11	0,000	0,009	0,981
rs7314285	12	CUX2	G	T	0,052	0,005	2.043e-25	-0,019	0,015	0,184
rs75938105	12	RP11-110L15.2	T	C	0,047	0,007	7.468e-12	-0,030	0,029	0,306
rs773116	12	ERBB3	G	A	0,016	0,003	1.265e-10	0,000	0,009	0,975
rs78607331	12	R3HDM2	C	T	0,037	0,006	6.059e-10	-0,011	0,027	0,687
rs9738365	12	RP11-428G5.4	A	C	0,058	0,003	1.905e-92	-0,007	0,010	0,479
rs1170158	13	DGKH	T	G	0,021	0,003	1.041e-10	-0,003	0,010	0,776
rs118081390	13	FNDC3A	G	A	0,028	0,005	1.353e-08	0,031	0,019	0,109
rs1535793	13	LRCH1	A	G	0,024	0,003	7.422e-17	0,050	0,010	0,000
rs6602909	13	GAS6	C	T	0,020	0,003	4.354e-14	0,032	0,010	0,001
rs7323205	13	LINC00676	C	T	0,015	0,003	9.354e-09	-0,010	0,009	0,264
rs9532512	13	LINC00598	A	G	0,043	0,003	8.024e-40	0,016	0,011	0,156
rs9573360	13	LINC00402	A	C	0,014	0,003	1.597e-08	-0,004	0,009	0,683
rs9583151	13	AL354741.1	C	T	0,014	0,003	2.708e-08	0,008	0,009	0,349
rs10136874	14	DLK1	G	T	0,023	0,003	2.350e-20	-0,011	0,010	0,260
rs10145154	14	NRXN3	T	C	0,018	0,003	9.924e-10	0,016	0,011	0,151
rs1061638	14	AHSA1	G	A	0,018	0,003	7.484e-11	-0,010	0,009	0,281
rs1115897	14	UNC79	A	C	0,021	0,003	1.540e-14	0,011	0,009	0,238
rs13379043	14	ELMSAN1	T	C	0,025	0,003	5.776e-18	-0,003	0,010	0,753
rs168961	14	ZFP36L1	G	A	0,018	0,003	2.889e-13	0,000	0,009	0,998
rs17106640	14	ACTN1	G	A	0,017	0,003	8.431e-11	-0,010	0,009	0,273
rs175043	14	EIF2B2	G	A	0,018	0,003	1.834e-12	0,008	0,009	0,349
rs28396553	14	AL162511.1	T	C	0,015	0,003	2.962e-09	-0,008	0,009	0,383
rs33912345	14	SIX6	C	A	0,023	0,003	2.363e-19	-0,005	0,009	0,565
rs78598185	14	SLC24A4	G	A	0,029	0,004	7.090e-11	0,014	0,016	0,393
rs79936318	14	SYNE2	A	G	0,017	0,003	3.875e-08	0,009	0,015	0,565
rs8017377	14	NYNRIN	G	A	0,017	0,003	3.353e-11	0,007	0,009	0,474
rs11856160	15	CHD2	A	G	0,021	0,003	2.843e-09	-0,013	0,013	0,310
rs12442867	15	RP11-299H22.1	A	C	0,017	0,003	9.329e-11	-0,004	0,009	0,663
rs12593755	15	RP11-97O12.3	T	G	0,016	0,003	1.846e-09	-0,009	0,009	0,323
rs12912439	15	LINC01197	T	C	0,022	0,003	1.246e-15	0,002	0,010	0,809
rs142354201	15	PGPEP1L	G	A	0,034	0,006	3.677e-09	-0,021	0,026	0,434
rs17747633	15	KNL1	G	A	0,015	0,003	3.080e-09	-0,002	0,010	0,848
rs2004839	15	RP11-299H22.3	G	A	0,021	0,003	4.336e-10	0,020	0,012	0,080
rs2311313	15	RP11-35O15.1	G	T	0,019	0,003	1.691e-08	0,010	0,012	0,412
rs2930313	15	CCDC33	G	A	0,028	0,005	4.141e-09	0,031	0,013	0,016
rs4545755	15	CYP19A1	G	A	0,016	0,003	3.865e-10	-0,015	0,009	0,085
rs55707100	15	MAP1A	C	T	0,151	0,008	1.406e-76	-0,049	0,031	0,121
rs5742915	15	PML	C	T	0,025	0,003	1.723e-22	0,009	0,010	0,396
rs79076440	15	USP3	A	G	0,019	0,003	1.164e-08	-0,009	0,012	0,461
rs8033075	15	PIAS1	A	G	0,045	0,005	1.061e-17	-0,017	0,022	0,446
rs11077337	16	ZNF597	T	G	0,015	0,003	1.454e-09	-0,004	0,009	0,626
rs11149612	16	RP11-505K9.4	C	T	0,027	0,003	1.131e-26	-0,004	0,009	0,687

rs111792934	16	HAS3	C	T	0,022	0,003	1.430e-10	0,019	0,011	0,100
rs116971887	16	SALL1	G	T	0,036	0,006	4.895e-09	-0,032	0,026	0,210
rs12597502	16	CHD9	G	A	0,015	0,003	4.528e-08	-0,015	0,010	0,112
rs12927172	16	IL4R	A	G	0,015	0,003	3.066e-09	-0,002	0,009	0,862
rs12935091	16	ZNF19	G	A	0,035	0,006	2.235e-09	0,017	0,025	0,515
rs12935465	16	XYLT1	T	C	0,016	0,003	5.769e-11	0,000	0,010	0,980
rs147491123	16	LINC01572	C	T	0,036	0,007	3.286e-08	0,062	0,029	0,033
rs1532824	16	ATF7IP2	A	C	0,017	0,003	4.521e-09	-0,003	0,010	0,757
rs1548917	16	RP11-461O7.1	T	C	0,015	0,003	2.361e-09	-0,005	0,009	0,552
rs1657125	16	MEIOB	T	G	0,032	0,004	2.007e-18	0,000	0,011	0,978
rs17299478	16	NOB1	C	T	0,032	0,003	2.497e-21	-0,017	0,012	0,148
rs2023762	16	SYT17	T	C	0,015	0,003	5.991e-09	-0,011	0,009	0,225
rs4786350	16	IFT140	C	G	0,036	0,006	6.048e-09	0,006	0,025	0,824
rs4788220	16	FAM57B	G	A	0,017	0,003	5.263e-12	-0,014	0,009	0,106
rs4985062	16	USP7	T	C	0,015	0,003	2.040e-09	0,001	0,009	0,918
rs61731445	16		C	T	0,028	0,005	1.958e-08	0,000	0,021	0,986
rs7204824	16	LMF1	C	T	0,024	0,003	1.168e-16	0,010	0,010	0,280
rs72761177	16	NUBP2	A	G	0,077	0,004	7.205e-70	-0,004	0,015	0,774
rs74774288	16	RP11-420N3.3	G	T	0,027	0,003	2.955e-17	0,002	0,013	0,847
rs7498665	16	SH2B1	G	A	0,019	0,003	7.173e-14	0,007	0,009	0,461
rs750952	16	ZNF646	C	T	0,032	0,003	1.105e-34	-0,003	0,009	0,707
rs753108	16	CMIP	A	G	0,025	0,003	1.606e-17	0,012	0,010	0,256
rs80253441	16	IGFALS	T	C	0,131	0,011	8.227e-36	0,049	0,036	0,175
rs8054054	16	CCDC154	G	A	0,015	0,003	1.026e-09	0,011	0,009	0,226
rs8054322	16	GSE1	A	G	0,015	0,003	4.723e-09	0,007	0,009	0,443
rs8059803	16	CMIP	A	G	0,031	0,003	4.415e-29	0,022	0,010	0,035
rs8182173	16	CORO7-PAM16	T	C	0,018	0,003	1.610e-09	-0,001	0,010	0,924
rs1801689	17	APOH	C	A	0,089	0,007	1.248e-33	0,063	0,038	0,094
rs199525	17	WNT3	G	T	0,020	0,003	5.613e-11	0,020	0,012	0,102
rs2309401	17	NLRP1	T	G	0,015	0,003	6.785e-09	0,011	0,010	0,250
rs35819807	17	KCNH6	T	C	0,017	0,003	2.387e-09	-0,001	0,011	0,923
rs3760237	17	SCN4A	C	T	0,024	0,003	1.441e-21	-0,001	0,009	0,921
rs4075483	17	BAIAP2	C	T	0,017	0,003	1.312e-10	0,005	0,009	0,620
rs4789227	17	UNK	T	C	0,015	0,003	4.335e-09	-0,012	0,009	0,186
rs56030650	17	GSDMA	C	A	0,022	0,003	2.417e-18	0,000	0,009	0,960
rs6416868	17	TTC19	A	G	0,019	0,003	3.115e-14	0,008	0,009	0,340
rs6501601	17	POLR3KP2	G	A	0,015	0,003	4.071e-09	0,004	0,009	0,665
rs668799	17	COASY	C	T	0,018	0,003	4.019e-10	-0,006	0,009	0,493
rs7502910	17	WDR81	A	G	0,016	0,003	1.528e-10	0,011	0,009	0,204
rs77542162	17	ABCA6	G	A	0,054	0,009	2.412e-10	0,043	0,038	0,259
rs8075153	17	RAI1	C	T	0,021	0,003	1.831e-17	0,012	0,009	0,173
rs8079923	17	AKAP10	C	T	0,016	0,003	3.664e-08	-0,013	0,011	0,222
rs9892862	17	Y_RNA	G	A	0,022	0,003	9.705e-14	-0,017	0,010	0,093
rs11152071	18	RP11-1151B14.2	C	T	0,020	0,003	8.957e-12	-0,002	0,010	0,832
rs12454712	18	BCL2	T	C	0,018	0,003	4.051e-12	-0,012	0,009	0,196

rs190102446	18	RP11-27G24.1	C	T	0,041	0,007	2.452e-09	-0,008	0,026	0,757
rs57551555	18	RP11-176N18.2	T	G	0,019	0,003	5.817e-13	-0,012	0,010	0,234
rs585187	18	MRPS5P4	T	G	0,015	0,003	3.712e-09	0,004	0,009	0,674
rs8084351	18	DCC	G	A	0,015	0,003	8.537e-10	-0,007	0,009	0,447
rs8095538	18	-	G	T	0,020	0,003	8.447e-14	0,000	0,010	0,999
rs8097893	18	GALR1	A	G	0,058	0,006	1.723e-20	0,013	0,019	0,501
rs11671304	19	ZC3H4	C	T	0,018	0,003	6.884e-11	-0,015	0,011	0,157
rs12975366	19	LILRB5	T	C	0,020	0,003	2.253e-15	0,018	0,011	0,110
rs2287922	19	RASIP1	G	A	0,030	0,003	1.833e-33	-0,023	0,009	0,017
rs296361	19	SULT2A1	A	G	0,025	0,003	3.421e-13	0,003	0,013	0,810
rs34536443	19	TYK2	G	C	0,045	0,006	9.964e-14	0,045	0,029	0,121
rs3760954	19	MIR7-3HG	T	C	0,024	0,004	2.909e-10	-0,001	0,013	0,939
rs58560372	19	SPINT2	C	T	0,020	0,003	1.331e-08	0,002	0,012	0,889
rs58658292	19	ZNF536	G	A	0,030	0,006	4.618e-08	0,005	0,020	0,808
rs62102136	19	LSM14A	C	T	0,016	0,003	6.527e-09	0,001	0,011	0,946
rs6510033	19	AC005597.1	A	G	0,020	0,003	1.147e-12	-0,001	0,009	0,902
rs6510177	19	ZNF536	T	C	0,023	0,003	1.598e-12	-0,010	0,014	0,466
rs6510832	19	KDM4B	G	T	0,033	0,005	2.638e-10	-0,001	0,020	0,950
rs67868323	19	ZBTB7A	T	G	0,016	0,003	2.205e-08	0,014	0,011	0,213
rs7254601	19	COX6B1	G	A	0,016	0,003	2.551e-08	-0,007	0,011	0,489
rs7256521	19	ZNF845	G	A	0,015	0,003	9.761e-10	-0,007	0,009	0,423
rs8105174	19	DNMT1	C	T	0,050	0,003	7.971e-54	-0,004	0,012	0,704
rs8112883	19	INSR	G	T	0,017	0,003	1.166e-09	-0,003	0,010	0,752
rs8113618	19	QTRT1	T	C	0,031	0,003	1.185e-33	0,030	0,009	0,000
rs16995311	20	PTPN1	A	C	0,040	0,005	5.559e-18	0,016	0,019	0,392
rs17265513	20	ZHX3	T	C	0,022	0,003	3.454e-12	-0,001	0,013	0,958
rs2424396	20	LINC01726	G	A	0,033	0,004	2.245e-14	0,005	0,015	0,728
rs2738787	20	TNFRSF6B	A	G	0,037	0,005	1.251e-15	-0,020	0,018	0,271
rs4809401	20	NPBWR2	T	C	0,023	0,004	6.488e-10	-0,008	0,016	0,593
rs6037508	20	SLC4A11	G	T	0,017	0,003	8.740e-09	0,001	0,010	0,910
rs6046825	20	RALGAPA2	A	C	0,024	0,003	2.108e-17	-0,007	0,009	0,467
rs6088579	20	NCOA6	G	A	0,027	0,003	8.509e-17	0,003	0,011	0,784
rs7267595	20	JAG1	A	C	0,015	0,003	9.437e-10	-0,004	0,009	0,610
rs7508949	20	CRNKL1	C	G	0,025	0,003	1.490e-22	-0,004	0,009	0,645
rs75989562	20	APMAP	A	G	0,030	0,005	8.093e-09	0,032	0,025	0,196
rs9978775	21	BRWD1-AS1	G	A	0,019	0,003	2.351e-13	-0,007	0,008	0,398
rs12106594	22	EIF4ENIF1	T	C	0,036	0,006	1.588e-09	-0,014	0,020	0,472
rs2412973	22	HORMAD2	A	C	0,014	0,003	3.334e-08	-0,010	0,009	0,276
rs4823324	22	ATXN10	T	C	0,016	0,003	1.938e-10	-0,007	0,009	0,434
rs5755948	22	RBFOX2	A	G	0,028	0,004	4.375e-14	-0,006	0,012	0,630
rs6519133	22	JOSD1	T	C	0,029	0,003	7.731e-30	-0,006	0,009	0,487
rs8138950	22	ZNRF3	C	T	0,015	0,003	1.323e-09	-0,003	0,009	0,722
rs9611565	22	TEF	T	C	0,029	0,003	3.825e-23	-0,004	0,010	0,691

Chr, chromosome; EA, effect allele; OA, other allele; SE, standard error.

ESM Table 6. Associations between insulin-like growth factor-1 levels and cardiometabolic diseases in multivariable Mendelian randomization analysis adjusted for fasting insulin levels

	**Unadjusted**[a]		**Adjusted**[a]	
**Outcome**	**OR (95% CI)**	***p* value**	**OR (95% CI)**	***p* value**
Type 2 diabetes	1.16 (1.07-1.25)	3.19×10^{-4}	1.12 (1.03-1.22)	0.007
Coronary artery disease	1.10 (1.01-1.19)	0.032	1.10 (1.01-1.19)	0.033
Heart failure	1.03 (0.97-1.09)	0.313	1.02 (0.96-1.09)	0.468
Atrial fibrillation	1.01 (0.95-1.08)	0.699	1.01 (0.94-1.08)	0.784
Ischemic stroke	1.04 (0.98-1.11)	0.232	1.05 (0.98-1.12)	0.147

[a]The number of single-nucleotide polymorphisms included in the analysis ranged from 293 to 299. The number of single-nucleotide polymorphisms do not sum up to the number included in the primary analyses of IGF-1 levels owing to missing single-nucleotide polymorphisms in the insulin dataset.

ESM Table 7. Associations between insulin-like growth factor-1 levels and cardiometabolic diseases in multivariable Mendelian randomization analysis adjusted for insulin resistance

	**Unadjusted**[a]		**Adjusted**[a]	
**Outcome**	**OR (95% CI)**	***p* value**	**OR (95% CI)**	***p* value**
Type 2 diabetes	1.16 (1.07-1.25)	3.19×10^{-4}	1.12 (1.03-1.22)	0.007
Coronary artery disease	1.10 (1.01-1.19)	0.032	1.10 (1.01-1.20)	0.027
Heart failure	1.03 (0.97-1.09)	0.313	1.02 (0.96-1.09)	0.468
Atrial fibrillation	1.01 (0.95-1.08)	0.699	1.01 (0.94-1.08)	0.871
Ischemic stroke	1.04 (0.98-1.11)	0.240	1.04 (0.98-1.11)	0.205

[a]The number of single-nucleotide polymorphisms included in the analysis ranged from 292 to 299. The number of single-nucleotide polymorphisms do not sum up to the number included in the primary analyses of IGF-1 levels owing to missing single-nucleotide polymorphisms in the insulin dataset.

ESM Table 8. Associations between insulin-like growth factor-1 levels and cardiometabolic diseases in multivariable Mendelian randomization analysis adjusted for height

	Unadjusted[a]		Adjusted[a]	
**Outcome**	**OR (95% CI)**	***p* value**	**OR (95% CI)**	***p* value**
Type 2 diabetes	1.16 (1.07-1.26)	2.16×10^{-4}	1.18 (1.09-1.28)	6.52×10^{-5}
Coronary artery disease	1.10 (1.01-1.19)	0.028	1.13 (1.04-1.22)	0.005
Heart failure	1.02 (0.96-1.08)	0.466	1.02 (0.96-1.08)	0.602
Atrial fibrillation	1.01 (0.94-1.07)	0.888	0.96 (0.90-1.02)	0.195
Ischemic stroke	1.04 (0.97-1.10)	0.258	1.05 (0.98-1.12)	0.163

[a]The number of single-nucleotide polymorphisms included in the analysis was 295 to 298. The number of single-nucleotide polymorphisms do not sum up to the number included in the primary analyses of IGF-1 levels owing to missing single-nucleotide polymorphisms in the height dataset.
